# Study protocol: randomised controlled hybrid type 2 trial evaluating the scale-up of two arts interventions for postnatal depression and Parkinson’s disease

**DOI:** 10.1136/bmjopen-2021-055691

**Published:** 2022-02-01

**Authors:** Tayana Soukup, Rachel E Davis, Maria Baldellou Lopez, Andy Healey, Carolina Estevao, Daisy Fancourt, Paola Dazzan, Carmine Pariante, Hannah Dye, Tim Osborn, Rebecca Bind, Kristi Sawyer, Lavinia Rebecchini, Katie Hazelgrove, Alexandra Burton, Manonmani Manoharan, Rosie Perkins, Aleksandra Podlewska, Ray Chaudhuri, Fleur Derbyshire-Fox, Alison Hartley, Anthony Woods, Nikki Crane, Ioannis Bakolis, Nick Sevdalis

**Affiliations:** 1 Centre Implementation Science, King's College London, London, UK; 2 Department of Psychological Medicine, King’s College London, London, UK; 3 Department of Behavioural Science and Health, University College London, London, UK; 4 Breathe Arts Health Research, London, UK; 5 South London and Maudsley NHS Foundation Trust, London, UK; 6 Faculty of Medicine, Imperial College London, London, UK; 7 Centre for Performance Science, Royal College of Music, London, UK; 8 Department of Basic and Clinical Neuroscience, King’s College London, London, UK; 9 English National Ballet, London, UK; 10 Department of Biostatistics and Health Informatics, King’s College London, London, UK

**Keywords:** maternal medicine, Parkinson-s disease, health economics, health services administration & management

## Abstract

**Introduction:**

Research on the benefits of ‘arts’ interventions to improve individuals’ physical, social and psychological well-being is growing, but evidence on implementation and scale-up into health and social care systems is lacking. This protocol reports the SHAPER-Implement programme (Scale-up of Health-Arts Programmes Effectiveness-Implementation Research), aimed at studying the impact, implementation and scale-up of: Melodies for Mums (M4M), a singing intervention for postnatal depression; and Dance for Parkinson’s (PD-Ballet) a dance intervention for Parkinson’s disease. We examine how they could be embedded in clinical pathways to ensure their longer-term sustainability.

**Methods and analysis:**

A randomised two-arm effectiveness-implementation hybrid type 2 trial design will be used across M4M/PD-Ballet. We will assess the implementation in both study arms (intervention vs control), and the cost-effectiveness of implementation. The design and measures, informed by literature and previous research by the study team, were refined through stakeholder engagement. Participants (400 in M4M; 160 in PD-Ballet) will be recruited to the intervention or control group (2:1 ratio). Further implementation data will be collected from stakeholders involved in referring to, delivering or supporting M4M/PD-Ballet (N=25–30 for each intervention).

A mixed-methods approach (surveys and semi-structured interviews) will be employed. ‘Acceptability’ (measured by the ‘Acceptability Intervention Measure’) is the primary implementation endpoint for M4M/PD-Ballet. Relationships between clinical and implementation outcomes, implementation strategies (eg, training) and outcomes will be explored using generalised linear mixed models. Qualitative data will assess factors affecting the acceptability, feasibility and appropriateness of M4M/PD-Ballet, implementation strategies and longer-term sustainability. Costs associated with implementation and future scale-up will be estimated.

**Ethics and dissemination:**

SHAPER-PND (the M4M trial) and SHAPER-PD (the PD trial) are approved by the West London and GTAC (20/PR/0813) and the HRA and Health and Care Research Wales (REC Reference: 20/WA/0261) Research Ethics Committees. Study findings will be disseminated through scientific peer-reviewed journals and scientific conferences.

**Trial registration numbers:**

Both trials are registered with NIH US National Library of Medicine, ClinicalTrials.gov. The trial registration numbers, URLs of registry records, and dates of registration are: (1) PD-Ballet: URL: NCT04719468 (https://eur03.safelinks.protection.outlook.com/?url=https%3A%2F%2Fwww.clinicaltrials.gov%2Fct2%2Fshow%2FNCT04719468%3Fterm%3DNCT04719468%26draw%3D2%26rank%3D1&amp;data=04%7C01%7Crachel.davis%40kcl.ac.uk%7C11a7c5142782437919f808d903111449%7C8370cf1416f34c16b83c724071654356%7C0%7C0%7C6375441942616) (date of registration: 22 Jan 2021). (2) Melodies for Mums: NCT04834622 (https://clinicaltrials.gov/ct2/show/NCT04834622?term=shaper-pnd&draw=2&rank=1) (date of registration: 8 Apr 2021).

**Strengths and limitations of this study**

- Scale-up of Health-Arts Programmes Effectiveness-Implementation Research-Implement is the largest known study of its kind, comprising multidisciplinary implementation and evaluation teams, with consistent stakeholder engagement embedded throughout.
- The study allows large-scale psychometric validation of newly developed implementation measures.
- Provides an example of how large-scale hybrid studies can be conducted within community settings using a synergistic methodology with broad applicability.
- Dance for Parkinson’s and Melodies for Mums will be trialled in specific geographic areas in England, further assessment of the interventions across England will be required to assess the wider benefit.

## Introduction

The use of arts interventions (ie, ‘creative methods of expression such as drama, music and visual arts’^1^) to improve health and social care outcomes and reduce service utilisation costs is an internationally growing area of research.^1–4^ In 2017, the UK’s All-Party Parliamentary Group on Arts, Health and Wellbeing published a report on the benefits of arts interventions, alongside ten stakeholder-led recommendations (from patients, health and social care professionals, artists, academics, charities, policy-makers and parliamentarians) on facilitating the implementation and scale-up of ‘arts’ into health and social care systems nationally.^1^ Two years on, the WHO’s scoping review of the global academic literature (2000–2019)^2^ identified over 900 publications, including 200 reviews, systematic reviews, meta-analyses and meta-syntheses covering over 3000 studies, and 700 additional studies. Taken the evidence collectively, arts interventions are an effective method to help treat a plethora of physical, social and psychological problems across the lifespan.^1–4^

Despite the promising evidence, progress on successfully embedding art interventions in health and social care systems has been slow. Presently, many ‘arts’ are delivered in small geographic or healthcare pockets, operating at the fringe of the care sector rather than receiving mainstream funding.^1 5^ While lack of sustainable funding, weak partnerships with commissioners and unclear referral pathways partly account for this^1 5^ research is required to establish cost-effective, scalable solutions so that the full benefits to the wider population can be reached.

The current protocol reports the design and evaluation of the ‘Scale-up of Health-Arts Programmes Effectiveness-Implementation Research’ (SHAPER-Implement)—part of a larger programme (referred to as ‘SHAPER’^6^ aimed at investigating ways to implement and deliver arts interventions at scale. We focus on two different health conditions: postnatal depression (PND) and Parkinson’s disease (PD). These represent a significant fiscal and public health burden and pose considerable affliction on the individuals affected (and, where applicable, their carers).^7–14^ While pharmacological treatments can be effective for controlling/alleviating symptoms,^7 9^ they are fraught with challenges: for PND, it is poor uptake and adherence,^15–18^ while for PD, it is overemphasis on treating the motor symptoms at the expense of non-motor functioning.^19–21^

Melodies for Mums (M4M) (for mums with PD) and Dance for Parkinson’s (PD-Ballet) (for individuals with PD) are two approaches to symptom management, that have been piloted with promising results.^22–30^ Both have already been implemented across certain locations in London, UK, but are not being delivered at scale, thus only reach a fraction of eligible individuals.

We plan to scale-up M4M/PD-Ballet and examine how we can embed them into clinical pathways so that a greater number of individuals can benefit. Our ambition is to be as inclusive as possible, reaching out to individuals who may not be undergoing treatment for their condition (as well as those that are), and ultimately for Clinical Commissioning Groups (eg, the ‘payers’ in the National Health Service, UK) to commission the interventions so they can be delivered in a sustainable way beyond the end of our research.

The entire SHAPER programme centres on tripartite objectives. Due to the complexity of the study design, this protocol reports the implementation effectiveness evaluation (ie, the SHAPER-Implement programme) of M4M/PD-Ballet (objectives 2 and 3, described below). The clinical effectiveness evaluation (objective 1) is reported in parallel protocols.^31 32^

### Objective 1

To assess the *clinical effectiveness* of M4M/PD-Ballet—described in detail in the clinical protocols.^31 32^

### Objective 2

To examine the *implementation effectiveness* of M4M/PD-Ballet, including uptake, adoption, perceived acceptability, appropriateness, feasibility, fidelity, unintended consequences and sustainability, and the impact of established implementation strategies (eg, training in the delivery of M4M/PD-Ballet) on implementation effectiveness.

### Objective 3

To assess *implementation costs and cost effectiveness*, including costs associated with implementing M4M/PD-Ballet into existing care pathways, health service, partner organisations and commissioning, costs to service users attending M4M/PD-Ballet vs the benefit in terms of quality-adjusted years of life lived, and the impact of M4M/PD-Ballet when delivered at scale, on the wider utilisation of healthcare and other services.

## Methods and analysis

### Design

SHAPER-Implement is a two-arm effectiveness-implementation hybrid type 2^33^ trial of M4M/PD-Ballet. Randomisation will be single-blinded (assessments performed by a blinded rater) and in a 2:1 ratio. M4M participants will receive the singing programme (intervention) or be encouraged to attend non-music classes in the community or online (control). PD-Ballet participants will receive a dance programme (dance-based training and a post-session Tea-and-Biscuit social time) or follow the standard treatment per the local pathway (control) and attend the post-training ‘Tea-and-Biscuit’ gatherings via an online platform.

Table 1 details how M4M/PD-Ballet meet the criteria proposed by Curran *et al*^33^ for a hybrid type 2 trial. Contextual constraints meant it was not feasible to randomise the implementation side of the trial (ie, allocate participants to different implementation strategies): but we will examine the effectiveness of the implementation strategies used to deploy M4M/PD-Ballet.

**Table 1:** Overview of the Curran *et al*^33^ criteria for hybrid trial type 2 design and how they apply to M4M and PD-Ballet hybrid evaluations

Study characteristics for hybrid trial type 2, as per Curran *et al* criteria^33^		Trialled arts interventions	
		Melodies for Mums	PD-Ballet
Research aims	**Determine feasibility and potential utility of an implementation intervention/strategy** Determine effectiveness of a clinical intervention*	**Determine feasibility and potential utility of M4M to facilitate future implementation and scale up** Determine clinical effectiveness of M4M	**Determine feasibility and potential utility of PD-Ballet to facilitate future implementation and scale up** Determine clinical effectiveness of PD-Ballet
Research questions	**Does the implementation method show promise (either alone or in comparison with another method) in facilitating implementation of a clinical treatment?** Will a clinical treatment work in these settings/ for these patients?*	**Will the implementation method show promise (alone) in facilitating M4M in people with PND?** What is the clinical impact of M4M?	**Will the implementation method show promise (alone) in facilitating PD-Ballet in people with PD?** What is the clinical impact of PD-Ballet?
Unit of randomisation	**Provider, clinical unit, facility or system, as per type although may be non-randomised, for example, case study** Patient or clinical unit, as per type 1	**Providers, as per type 3 (non-randomised**) Patients, as per type 1 (randomised)	**Providers, as per type 3 (non-randomised**) Patients, as per type 1 (randomised)
Comparison conditions	**Provider, clinical unit, facility, system: implementation as usual, or competing implementation strategy although may be non-randomised, for example, case study** Placebo, treatment as usual, or competing treatment, as per type 1	**Facility, as per hybrid type 3** Treatment as usual, as per hybrid type 1	**Facility, as per hybrid type 3** Treatment as usual, as per hybrid type 1
Sampling frames	**Providers/clinics/facility/systems: consider ‘optimal’ cases** Patient: limited restrictions, but some inclusion/exclusion criteria	**Providers/facility: artists, psychiatrists/neurologists, referrers, commissioners, support staff; community centres** Patient: inclusion/exclusion criteria used	**Providers/facility: artists, psychiatrists/neurologists, referrers, commissioners, support staff; dance centres** Patient: inclusion/exclusion criteria used
Evaluation methods	**Mixed method; quantitative, qualitative; formative and summative** Quantitative, summative	**Quantitative surveys (AIM,^60^ IAM,^60^ FIM,^60^ NoMaD,^64^ costing proforma) and interviews**^62^ Quantitative surveys (EPDS^65^; EQ-5D 3L^63^)	**Quantitative surveys (AIM,^60^ IAM,^60^ FIM,^60^ NoMaD,^64^ costing proforma) and interviews**^62^ Quantitative surveys (†EPDS^65^; EQ-5D 3L^63^)
Measures	**Adoption of clinical treatment and fidelity to it, as well as related factors** Patient symptoms and functioning, possibly cost effectiveness	**Adoption of M4M, fidelity of its delivery and receipt, acceptability, appropriateness, feasibility, sustainability, reach, unintended consequences, contextual factors, implementation strategies, implementation costs** Patient symptoms and functioning, cost effectiveness	**Adoption of PD-Ballet, fidelity of its delivery and receipt, acceptability, appropriateness, feasibility, sustainability, reach, unintended consequences, contextual factors, implementation strategies, implementation costs** Patient symptoms and functioning, cost effectiveness

Bold type: information relating to the implementation effectiveness evaluation. Normal type: information relating to the clinical effectiveness evaluation, discussed in more detail in a separate protocols^31 32^

*Curran *et al*: ‘one of these aims/research questions might take precedence, for example in a case where the test of an implementation intervention/strategy is exploratory’.^33^

AIM, Acceptability of Intervention Measure; EPDS, Edinburgh Postnatal Depression Scale; FIM, Feasibility of Intervention Measure; IAM, Intervention Appropriateness Measure; M4M, Melodies for Mums; NMSS, Non-Motor Symptoms Scale; NoMaD, Implementation measure based on Normalization Process Theory; PD-Ballet, Dance for Parkinson’s; PND, postnatal depression.

### Setting

SHAPER-Implement is a multisite, multidisciplinary, community-based study in London, UK. Funded by the Wellcome Trust, it is a collaboration between the Centre for Implementation Science, King’s College London; King’s Health Partners; the Department for Behavioural Science and Health and the Institute of Mental Health, University College London, and two award-winning arts organisations: Breathe Arts Health Research and English National Ballet (ENB). King’s holds a long-established commitment to embedding arts, health and well-being in education and research^34 35^ and was the research partner for landmark publications in the area.^1^

### Health conditions

*PND* is a serious and the most common perinatal mental health condition, affecting 10%–20% of women in pregnancy and after birth.^11^ If left untreated the impact on women and their families can be devastating^11 36 37^ with symptoms including fatigue, anhedonia, insomnia and irritability and thoughts of suicide.^7 8 37 38^ Therapy (eg, cognitive-behavioural therapy) or medication can be prescribed to help treat PND but progress can be slow, may involve long wait times for treatment (for therapy) or prolonged use of medication^38–40^—thus other options for symptom management need to be explored.

*PD* is one of the world’s fastest growing chronic neurodegenerative disorders, with those aged over 70 being particularly vulnerable.^14 41 42^ It affects over 145 000 people in the UK alone^42^ with prevalence rates expected to rise by around 18% between 2018 and 2025 (to over 168 000), and doubling by 2065.^10 42^ Symptoms relate to motor (eg, tremor, bradykinesia, freezing of gait) and non-motor (eg, sleep disturbance, drooling, cognitive decline)^9 10^ functioning. Currently, there is no cure for PD—and the disease has a progressive course.^9 10^ Pharmacological treatments can alleviate symptoms and improve quality of life but are largely aimed at addressing motor functioning, leaving the non-motor symptoms often unrecognised or under-treated.^10 43 44^ Alternative non-pharmacotherapies are necessary to slow down disease progression.^10 43 44^

### Interventions

Drawing from Curran *et al*’s ‘hybrid’ framework^33^ (see table 1) through an intensive 4-month stakeholder engagement with artists, researchers, clinicians and commissioners, we considered five elements critical to assessing the relevance of the interventions:

- *Suitability*: M4M/PD-Ballet address a need within the health sector—there are growing patient populations with the conditions and lack of effective current service provision.
- *Quality and face validity*: M4M/PD-Ballet are ‘high-quality’ interventions with carefully designed and tailored activities, developed in partnership between centres of academic excellence and renowned arts organisations.
- *Inclusivity*: M4M/PD-Ballet are ‘all-inclusive’. They have good uptake, not only with those already engaged in the arts. They also appear to ‘reach-out’ to individuals that are disengaged in other forms of treatment for PND/PD (eg, pharmacological approaches).
- *Effectiveness*: there is evidence to show M4M/PD-Ballet improves symptoms and quality of life and can achieve better adherence than pharmacological approaches.^23–26 28–30^
- *Scalability:* M4M/PD-Ballet are not overly complex, thus have the potential to be scaled-up, embedded in clinical pathways and commissioned by the health sector.^6^

*M4M*^6 31^ is delivered in partnership with Breathe Arts Health Research (https://breatheahr.org/melodies-for-mums/). A breathe-trained artist (the workshop leader) and support assistant deliver ten weekly singing sessions to a group of mums with PND and their babies. Classes start with welcome songs, followed by music activities, ranging from short vocal exercises and simple lullabies to longer songs that attendees learn gradually over the weeks. Songs can be relaxing, with mothers encouraged to hug/stroke their babies as they sing, or energetic, with mothers standing and moving with their babies. Instruments (eg, guitars and ukuleles) are used by the workshop leader, accompanied by simpler instruments for mothers/babies to use. Mothers are encouraged to write their own songs, developing lyrics together relating to their babies or experiences of motherhood.^6 31^ Recordings of the singing sessions are made available to attendees at the end.

M4M has been subject to a three-arm randomised controlled trial (RCT) of 134 mothers and a preliminary process evaluation. Significantly faster improvements in symptoms for mothers with moderate-severe PND (measured by the Edinburgh Postnatal Depression Scale (EPDS)), than mothers in usual care^24^ were observed. Levels of depression consistently declined—by week 6, 65% of mothers no longer had an EPDS ≥13 (ie, indicating no more than mild depression); increasing to 73% by week 10.^24^ Increase in the frequency of mothers singing to their babies outside the classes, perceived mother–infant closeness and a greater decrease in cortisol levels when compared with social play were also reported.^23–25^ The process evaluation showed that M4M reached the correct target demographic, was delivered with a high level of fidelity and programme satisfaction was high—88% of mothers agreed the classes were well tailored, and 100% would recommend M4M to another mother.^26 29 30^ Several challenges, however, were highlighted by mothers, workshop leaders and the project coordinator (eg, timing sessions with babies’ routines)—while this did not hinder the continuation of M4M, it nonetheless suggests that implementation of M4M could be improved.^26^

*PD-Ballet*^6 32^ is based on the ENB’s pre-existing ‘Dance for Parkinson’ (DfP) programme (referred to as PD-Ballet for the SHAPER research) and delivered in partnership with the ENB (https://www.ballet.org.uk/project/dance-for-parkinsons/). Led by an ENB trained DfP Associate Artist and Associate Musician who deliver 12 weekly sessions to individuals with PD (carers/relatives can also attend), sessions comprise live music, dance and vocal exercises. Each session comprises 75 min of activity, followed by social time and refreshments (up to 1 hour) so that participants can get to know fellow attendees and form social networks. Content, inspired by the ENB’s classical and contemporary works, provides a framework for participants to explore narrative, themes, concepts and music to promote freedom of expression. A performance sequence is developed at the end of PD-Ballet, combining all elements of the programme. Dance material is adapted to be inclusive (catering for differing levels of mobility) so that everyone can participate fully. For PD-Ballet content will be further developed for the three distinct stages of motor advancement (mild/moderate/severe)—something which has not been done before within the existing DfP programme. Prior to the programme, individuals attend an introductory session with their assigned group and are given the opportunity to attend a ballet performance and a behind the scenes event.^6 32^

Initial testing of PD-Ballet in London, and its regional affiliated hub partners, has proven to be replicable, resulting in it being trialled and established in four other locations in the UK (Oxford, Cardiff, Ipswich and Liverpool). An independent evaluation led by the University of Roehampton (on scaling-up the programme, experiences and benefits of participation across 4 years and the effects on the body, daily activities and social participation) reported high levels of perceived value from an emotional, social and artistic perspective.^28^ Additionally, PD-Ballet can decrease social isolation and improve quality of life,^22^ with participants highly motivated and viewing PD-Ballet as an important part of their lives.^27^ Equally, while recent systematic reviews and studies on the effects of performing acts modalities (including dance and ballet) reported promising benefits on Parkinson’s symptoms^45 46^ very few RCTs have investigated the benefits of ‘dance’ in both motor and non-motor symptoms, pointing to the need for this research.

SHAPER-Implement will build on previous research—conducting a full RCT (for PD-Ballet), a follow-up RCT scaled to a larger number of mothers (for M4M), and a process evaluation and examination of the implementation and potential cost-effectiveness of delivery at scale (for both) (see table 2 for further details on M4M/PD-Ballet).

**Table 2:** Overview of the structure and delivery of Melodies for Mums and PD-Ballet interventions

Programme structure and delivery	Melodies for Mums	PD-Ballet
Arts partner	Breathe Arts Health Research	English National Ballet
Targeted health condition	Postnatal depression	Parkinson’s disease
Setting	Community children’s centres OR online (depending on COVID-19 guidelines)	Community dance studios OR online (depending on COVID-19 guidelines)
Delivery structure	A weekly 1-hour group session over 10 weeks	A weekly 2 hours 15 min 12-group session over 12 weeks
N of intervention cycles	10 cycles, with a morning and afternoon cycle held across 5 separate10 weekly periods	3 cycles (stratified by motor advancement—mild/moderate/severe)
N of participants per cohort	12–15	30–35
Total N of participants in the intervention	270 mums (plus their respective babies)	107 individuals with Parkinson’s*
Follow-ups (as related to the delivery of sessions)	No follow-up	No follow-up
Artists per session	2 (1 artist, 1 assistant)	4 (2 associate dance artists/2 associate musicians), plus 1 *artist-in-training*
Total N of participants in the control	N=130 mums	N=53

All those involved in M4M and PD-Ballet will receive training in a standardised format beforehand the delivery of the programmes.

*The three cohorts differ in the severity of Parkinson’s symptoms: mild, moderate and severe

†The 2 days training is required for each stage of motor advancement so the trainer would need to attend three 2 day training sessions if they running programmes for all three groups of motor advancement (mild/moderate/severe).

N, number; PD-Ballet, Dance for Parkinson’s.

### Implementation and adaption: COVID-19 pandemic

As a result of the first national lockdown in the UK on 23 March 2020 and the guidelines that followed, M4M/PD-Ballet were adapted to be delivered online and preliminary research into the feasibility of using an online platform is underway. While the plan moving forward (ie, when the trials begin) will be to deliver PD-Ballet and M4M face-face, this will be continually reviewed based on the government guidelines on COVID-19 at the time, and the switch to remote delivery of the programmes will be made if necessary.

### Theoretical underpinning

The Medical Research Council’s (MRC) framework for evaluating complex interventions^47 48^ informed the study design for intervention development, implementation and evaluation processes.

The ‘Implementation Science Research Development’ tool,^49^ allowed us to operationalise the MRC guidance into the overall design of SHAPER-Implement, along with the research team’s knowledge and experience of conducting hybrid trials.^50^ We also tailored intervention delivery using the COM-B model^51^ by considering factors that could affect individuals’ capability, opportunity and motivation to engage with M4M/PD-Ballet.

The Consolidated Framework for Implementation Research (CFIR)^52 53^ will be used to assess factors affecting the implementation M4M/PD-Ballet. The ‘Expert Recommendations for Implementing Change’ (ERIC) project’s compendium of implementation strategies^54 55^ will guide the identification of implementation strategies that could overcome some of the factors affecting implementation.

The Reach Effectiveness Adoption Implementation Maintenance (RE-AIM) framework,^56 57^ which comprises domains on ‘implementation’ and ‘effectiveness’ and is suited to address challenges of blended effectiveness-implementation designs, guided the selection of implementation measures alongside Proctor *et al*^43^ taxonomy of implementation outcomes.

### Participants

For M4M, 400 mothers (with their babies) will be randomised 2:1 to the intervention (N=270) versus control (N=130). Mothers over 18 years of age with symptoms of PND (defined as a diagnosis of major depressive disorder according to the Structured Clinical Interview for DSM-IV Disorders and a score of ≥10 on the EPDS) and a baby 0–9 months old, will be eligible. Mothers with an EPDS score of <10 will be excluded and signposted to other support services within the community (eg, mother–baby groups) but invited to re-screen for future cycles of the intervention (in case their symptoms change).

For PD-Ballet, 160 individuals will be recruited: 2:1 to the intervention (N=107) versus control (N=53). Participants will be 18 years old or over with a diagnosis of idiopathic PD according to the UK PD Brain Bank criteria and Hoehn Yarhr stages I–V. Individuals with a diagnosis of other Parkinsonism or an indication of dementia through a score of ≤21 on the Montreal Cognitive Assessment will be excluded.

Individuals (for both M4M/PD-Ballet) will also be excluded if they are unable to: understand English; give informed consent; access and attend the singing/dance sessions online (due to COVID-19) through an internet-connected device (eg, mobile phone, computer), or face-to-face (government guidelines permitting).

Individuals who decline participation or withdraw from M4M/PD-Ballet will be asked (providing they are willing) to provide their reason(s), so that barriers to uptake (from ‘decliners’) or continued participation (from ‘withdrawers’) can be identified and addressed.

SHAPER-Implement will also collect data on stakeholders’ attitudes and experiences of M4M/PD-Ballet (N=25–30, respectively) so that challenges relating to intervention delivery can be reviewed and overcome.

A description of power and sample size calculations for M4M/PD-Ballet can be found in the corresponding clinical protocols.^31 32^

### Patient and public involvement

We are engaging with members of the public and patients throughout SHAPER-Implement and at every stage of the research process. We have formed advisory groups comprising individuals who have already completed the PD-Ballet and M4M programme. To date, these individuals have provided feedback on (but not limited to) our study design, data collection measures, recruitment strategies and methods for disseminating findings.

### Wider stakeholder engagement

In addition to patients/the public, engagement with other stakeholders is also critical to the success and scalability of M4M/PD-Ballet. We will hold regular meetings with: ‘deliverers’—artists/staff involved in delivering M4M/PD-Ballet; ‘referrers’—those who refer individuals to M4M/PD-Ballet (eg, general practitioners or neurologists (PD-Ballet); psychiatrists, health visitors and midwives (M4M) and; ‘supporters’—additional staff who support the running of M4M/PD-Ballet. To date, examples of involvement include contributing to the overall study design; critically reviewing methods of assessment for relevance and clarity; and exploring and facilitating referral pathways for recruitment.

### Developing theory of change

We will conduct stakeholder workshops to develop theory of change (ToC) and logic models. The ToC element will focus on summarising M4M/PD-Ballet at a strategic level, capturing possible pathways for the change process to achieve our outcomes. The logic models will focus on the programme specifics, assessing the change process at the level of implementing M4M/PD-Ballet. We will advance the traditional logic model by drawing from the recently developed implementation research logic model.^58^ We will examine inputs (eg, time), activities (eg, recruitment), outputs (eg, publications), outcomes (eg, symptoms), implementation determinants (identified in the CFIR^52 53^), implementation strategies, (drawing from the ERIC project^54 55^) and mechanisms of action resulting from the implementation outcomes (informed by the implementation outcome taxonomy^59^ and RE-AIM).^56 57^

### Recruitment

Recruitment for M4M will be through: (1) signposting via health and social care professionals, including midwives and health visitors; (2) healthcare referrals, including general practitioners, clinical psychologists, psychiatrists and self-referral; (3) weighing clinics and other community and clinical centres for postnatal mothers, and; (4) social media groups and online forums aimed at new mothers (eg, of the consent form, refer to online supplemental file 1)

10.1136/bmjopen-2021-055691.supp1 Supplementary data

Recruitment for PD-Ballet will be through: (1) the Movement Disorders Outpatient Clinic at King’s College Hospital; (2) London South Parkinson’s Excellence Network (PEN) using the London South PEN patient section of the website; (3) London South and National Clinical Research Network websites and the EUROPAR website https://parkinsons-london.co.uk/europar/; (4) the Study Hub website (Parkinson’s UK) and; (5) the ENB.

Stakeholders will be recruited through Breathe’s (for M4M) and ENB’s (for PD-Ballet) network of artists, staff and others involved in M4M/PD-Ballet.

The start and end dates for the trials are September 2021–December 2023 (for M4M) and February 2022–December 2022 (for PD-Ballet). Follow-up data will be collected at 36 weeks so the full data collection for the trials will end in May 2024 (for M4M) and May 2023 (for PD-Ballet). It should be noted that more than one cycle of each programme will run for each trial, and that these cycles may overlap (ie, occur simultaneously) to varying degrees for M4M/PD-Ballet—hence, the slightly different durations of each of the trials.

### Measures

Measures will be standardised across M4M/PD-Ballet, piloted and further refined (where applicable) through a codesign process with stakeholders to ensure their suitability.

Data on the acceptability^60^ of M4M/PD-Ballet (primary implementation outcome measure) and training in the delivery of M4M/PD-Ballet (SHAPER-Implement’s established implementation strategy), and data on the feasibility^60^ and appropriateness^60^ will be collected at three timepoints.

Implementation costs will be assessed using a previously developed costing proforma^61 62^ on the time and financial resource associated with implementing M4M/PD-Ballet within existing care pathways. To explore commissioning costs, feedback from provider organisations will be sought (those responsible for providing the interventions) to establish a range of plausible unit prices that the National Health Service (NHS)/partner organisations would be required to pay. To establish the impact of M4M/PD-Ballet on the wider utilisation of services (ie, other health and service costs), service utilisation questionnaires on the frequency of contact with other NHS/non-NHS services over a 3-month retrospective period will be completed. Reported service use will be costed using published unit cost estimates for healthcare and other services. The EQ5D 3L^63^ will be used to evaluate quality of life outcomes and quality-adjusted life year outcomes in the short-term.

To explore sustainment of M4M/PD-Ballet, the ‘Normalisation Measure Development’ questionnaire^64^ will be administered postintervention.

Enrolment onto M4M/PD-Ballet will assess uptake and participation data and attrition rates will be used to assess adherence.

Sociodemographic data on participants and stakeholders will be collected (see table 3 for further details on measures).

**Table 3:** Data collection plan for the implementation effectiveness evaluation of Melodies for Mums and PD-Ballet interventions

Outcome	Definition	Form of measurement	Timepoint(s)	Stakeholder group
**Implementation effectiveness**				
Acceptability	Extent to which M4M/PD-Ballet are perceived to be agreeable and acceptable for management of PND and PD, respectively. Extent to which training in the delivery of M4M/PD-Ballet (ie, SHAPER-Implement’s established implementation strategy) is considered acceptable to arts leads/artists.	AIM survey Interview	During intervention, postintervention and 3–6 months postintervention follow-up	Participants and wider stakeholder groups
Appropriateness	Extent to which M4M/PD-Ballet are perceived to be fit and relevant for management of PND and PD, respectively.	IAM survey Interview	During intervention, postintervention and 3–6 months postintervention follow-up	Participants and wider stakeholder groups
Feasibility	Extent to which M4M/PD-Ballet can be successfully used or carried out to help manage and improve PND and PD-related effects.	FIM survey Interview	During intervention, postintervention and 3–6 months postintervention follow-up	Participants and wider stakeholder groups
Fidelity of delivery	Extent to which M4M/PD-Ballet sessions were delivered according to protocol.	Interview	Postintervention	Deliverers
Fidelity of receipt	Extent to which M4M/PD-Ballet are received as intended.	Interview	Postintervention	Participants
Adoption	Intention to adopt and use the knowledge and skills learnt in M4M/PD-Ballet in everyday management of PND and PD.	Interview	Postintervention	Participants and wider stakeholder groups
Sustainability	Facilitators and barriers to sustained use of M4M/PD-Ballet.	Interview and the NoMad survey	Postintervention and follow-up	Participants and wider stakeholder groups
Uptake	The proportion and representativeness of individuals willing to participate in M4M/PD-Ballet.	Trial records	Baseline	Provided by trial manager
Adherence	The number of individuals attending each M4M/PD-Ballet session and the number of dropouts	Trial records	Throughout intervention period	Provided by trial manager
Unintended consequences	Positive or negative consequences that are not anticipated at the time of implementation of M4M/PD-Ballet.	Interview	Postintervention	Participants and wider stakeholder groups
Implementation strategies*	Strategies used to deliver and implement M4M/PD-Ballet.	Interview	Postintervention	Wider stakeholder groups
**Implementation costs and cost effectiveness**				
Implementation costs	Costs associated with prospective implementation of M4M/PD-Ballet.	Costing proforma	Postintervention	Participants and wider stakeholder groups
Cost-benefit analysis	Costs associated with M4M/PD-Ballet vs the benefits in terms of the outcomes.	EQ-5D 3L	During and postintervention	Participants
**Clinical effectiveness†**				
	The impact of M4M/PD-Ballet on the clinical outcomes of interest.	EPDS survey NMSS survey	Baseline, postintervention and 3–6 months postintervention follow-up	Participants

*As part of SHAPER-Implement we will be assessing the acceptability and benefit of ‘training arts leads/artists’ in the delivery of M4M/PD-Ballet (ie, our established implementation strategy) but we will also be examining other implementation strategies that may be of use in the wider-scale implementation of M4M/PD-Ballet.

†For further information on the clinical effectiveness evaluations, please refer to the corresponding clinical protocols for M4M^31^ and PD-Ballet.^32^

AIM, Acceptability of Intervention Measure; EPDS, Edinburgh Postnatal Depression Scale (Melodies for Mums); FIM, Feasibility of Intervention Measure; IAM, Intervention Appropriateness Measure; M4M, Melodies for Mums; NMSS, Non-Motor Symptoms Scale (Dance for Parkinson’s); NoMAD, Implementation measure based on Normalization Process Theory; PD, Parkinson’s disease; PD-Ballet, Dance for Parkinson’s; PND, postnatal depression; SHAPER, Scale-up of Health-Arts Programmes Effectiveness-Implementation Research.

### Qualitative measures

Semi-structured interviews partly informed by the study team’s methods of assessment for previous hybrid trials^50 62^ will complement the quantitative findings. Interviews will occur post-intervention and examine:

- Implementation strategies used to deliver M4M/PD-Ballet alongside strategies that may be important to consider by the local centres wishing to implement M4M/PD-Ballet after completion of SHAPER-Implement.
- Factors affecting the perceived acceptability, appropriateness and feasibility of M4M/PD-Ballet.
- Fidelity of delivery—whether M4M/PD-Ballet is delivered in accordance with protocol (eg, number and length of sessions, format and content covered).
- Fidelity of receipt—how the content of M4M/PD-Ballet is received by participants, including their experiences of engagement, comprehension of the content delivered, and application of acquired knowledge and skills to daily life.
- Factors affecting the sustained use of the knowledge and skills acquired from M4M/PD-Ballet for the long-term management of PND/PD (from participants) and the implementation of M4M/PD-Ballet into local services after the completion of SHAPER-Implement (from stakeholders).
- Unintended consequences (positive and negative) of M4M/PD-Ballet
- Stakeholders’ willingness for continued involvement in M4M/PD-Ballet (ie, intention to adopt) and participants’ intentions to use the knowledge and skills gained from M4M/PD-Ballet and recommend M4M/PD-Ballet to others.

### Data analysis

#### Quantitative implementation outcomes

Parametric and non-parametric tests will be employed to compare survey responses between the two arms of the trial for M4M/PD-Ballet and the established implementation strategy (ie, training arts leads/artists). Linear, logistic and Poisson regression models (depending on the distribution of the outcome) will explore the relationship between implementation (Acceptability of Intervention Measure (AIM), Intervention Appropriateness Measure (IAM) and Feasibility of Intervention Measure (FIM)^60^ and clinical trial primary (EPDS^65^ for M4M and MDS-MNS^66^ for PD-Ballet) and secondary outcome. Potential moderators (eg, age of the age of the baby for M4M) of the effect of implementation (AIM, IAM and FIM) on clinical trial primary (EPDS^65^ for M4M and MDS-MNS^66^ for PD-Ballet) and secondary outcome will be explored with the inclusion of an interaction term. Mediation analysis using structural equation models under the causal framework will be employed to understand the potential pathways in which implementation outcomes and the implementation strategy of ‘training’, impact on the effectiveness of M4M/PD-Ballet.^67 68^ All analyses will be conducted in STATA V.16.0.

#### Psychometric assessment of the reliability, validity and factorial structure of the implementation survey scales

The implementation outcomes scales are relatively new, hence require psychometric assessment. Cronbach’s coefficient alpha will evaluate the reliability (internal consistency) of the IAM, FIM and AIM scale-items (values range from 0 to 1 with internal consistency deemed acceptable when Cronbach’s alpha^69^ is ≥0.70^70^). A confirmatory factor analysis model will be fitted, using the weighted least square estimator with a mean-and-variance-adjusted χ^2^ method to handle ordered categorical items.^71^ To evaluate overall model fit, the comparative fit index (CFI),^72^ the Tucker Lewis index^73^ and the root mean square error of approximation (RMSEA)^74^ will be calculated. A CFI and TLI value of >0.90 indicates adequate fit to the data.^75^ A value of RMSEA <0.05 indicates close fit, values between 0.05 and 0.08 suggest adequate model fit, and values >0.10 suggest poor model fit.^75^ Psychometric analysis will be conducted using STATA V.16 and Mplus V.7.4.^76^

#### Health economic outcomes

Using a combination of empirical and modelling methods, we will bring together evidence on clinical endpoints, quality-of-life, implementation and intervention costs, and epidemiological data on prevalence and service utilisation to gauge the cost-effectiveness of delivering M4M/PD-Ballet at scale. We will build in evidence-based assumptions regarding the longer-term health and service resource impacts beyond the observable trial period. The cost-effectiveness of scalability under differing scenarios will be assessed with reference to existing thresholds, inclusive of those currently used by the National Institute for Health and Care Excellence for assessing programme cost-effectiveness. Uncertainty in estimates will be explored through probabilistic and one-way sensitivity analysis. Cost-effectiveness will be evaluated from an NHS perspective, with a focus on exploring the incidence of incremental resource impacts associated with utilisation of NHS funded resources.

#### Qualitative implementation outcomes

A systematic classification process of coding themes/patterns in the data will be used with the main themes predetermined deductively by the Proctor *et al*^59^ implementation outcomes framework and RE-AIM.^77^ Subthemes will be shaped inductively with the data until saturation is achieved. CFIR^78 79^ will be used to analyse barriers/facilitators (anticipated and actual) to the implementation and sustainment of M4M/PD-Ballet. NVivo V.12 will be used for data analysis.

## Ethics and dissemination

M4M and PD-Ballet have been reviewed and approved by the West London and GTAC Research Ethics Committee (Reference: 20/PR/0813) and the HRA and Health and Care Research Wales Research Ethics Committee (Reference: 20/WA/0261). Informed consent will be sought from all research participants. The results will be disseminated in academic journals and conferences as well as other channels (including to patients and the public in the centres and healthcare settings where the programmes are being delivered and through social media).

## Supplementary Material

Reviewer comments

Author's manuscript

## References

[1] All-Party Parliamentary Group on Arts. Health and wellbeing inquiry report. creative health: the arts for health and wellbeing. All-Party parliamentary group on arts, 2017.

[2] Fancourt D, Finn S. What is the evidence on the role of the arts in improving health and well-being? A scoping review. who regional office for Europe, 2019. 32091683

[3] Stuckey HL, Nobel J. The connection between art, healing, and public health: a review of current literature. Am J Public Health 2010;100:254–63. 10.2105/AJPH.2008.156497 20019311 PMC2804629

[4] Boyce M, Bungay H, Munn-Giddings C, et al. The impact of the arts in healthcare on patients and service users: a critical review. Health Soc Care Community 2018;26:458–73. 10.1111/hsc.12502 28940775

[5] Eccles MP, Mittman BS. Welcome to implementation science. Implementation Sci 2006;1. 10.1186/1748-5908-1-1

[6] Estevao C, Fancourt D, Dazzan P, et al. Scaling-up Health-Arts programmes: the largest study in the world bringing arts-based mental health interventions into a national health service. BJPsych Bull 2021;45:32–9. 10.1192/bjb.2020.122 33355526 PMC8058867

[7] Boath E, Henshaw C. The treatment of postnatal depression: a comprehensive literature review. J Reprod Infant Psychol 2001;19:215–48. 10.1080/02646830120073224

[8] Morrell CJ, Warner R, Slade P, et al. Psychological interventions for postnatal depression: cluster randomised trial and economic evaluation. The ponder trial. Health Technol Assess 2009;13:1–153. 10.3310/hta13300 19555590

[9] National Institute for Health Care Excellence. Parkinson’s disease in adults: National Institute for Health and Care Excellence, 2017.

[10] UK Parkinson’s. UK Parkinson’s audit - Transforming care. UK Parkinson’s, 2017.

[11] Bauer A, Parsonage M, Knapp M. The costs of perinatal mental health problems. centre for mental health and London school of economics, 2014.

[12] Parkinson’s UK. The cost of Parkinson’s: the financial impact of living with the condition. Parkinson’s UK, 2017.

[13] Soundy A, Stubbs B, Roskell C. The experience of Parkinson's disease: a systematic review and meta-ethnography. ScientificWorldJournal 2014;2014:1–19. 10.1155/2014/613592 PMC4265687 25525623

[14] Yang W, Hamilton JL, Kopil C, et al. Current and projected future economic burden of Parkinson’s disease in the U.S. NPJ Parkinsons Dis 2020;6:15. 10.1038/s41531-020-0117-1 32665974 PMC7347582

[15] Bledsoe SE, Grote NK. Treating depression during pregnancy and the postpartum: a preliminary meta-analysis. Res Soc Work Pract 2006;16:109–20. 10.1177/1049731505282202

[16] Cooper PJ, Murray L, Wilson A, et al. Controlled trial of the short- and long-term effect of psychological treatment of post-partum depression. I. impact on maternal mood. Br J Psychiatry 2003;182:412–9. 10.1192/bjp.182.5.412 12724244

[17] Milgrom J, Negri LM, Gemmill AW, et al. A randomized controlled trial of psychological interventions for postnatal depression. Br J Clin Psychol 2005;44:529–42. 10.1348/014466505X34200 16368032

[18] Cooper PJ, Murray L, Halligan SL. Treatment of postpartum depression. Winnicott Research Unit, University of Reading, UK: Centre of Excellence for Early Childhood Development, 2010.

[19] Booth FW, Roberts CK, Laye MJ. Lack of exercise is a major cause of chronic diseases. Compr Physiol 2012;2:1143–211. 10.1002/cphy.c110025 23798298 PMC4241367

[20] Chaudhuri KR, Titova N, Department of Neurology, Neurosurgery and Medical Genetics, Pirogov Russian National Research Medical University, Moscow, Russian Federation. Societal burden and persisting unmet needs of Parkinson’s disease. Eur Neurol Rev 2019;14:28–35. 10.17925/ENR.2019.14.1.28

[21] Martinez-Martin P, Rodriguez-Blazquez C, Kurtis MM, et al. The impact of non-motor symptoms on health-related quality of life of patients with Parkinson's disease. Mov Disord 2011;26:399–406. 10.1002/mds.23462 21264941

[22] Bognar S, DeFaria AM, O’Dwyer C, et al. More than just dancing: experiences of people with Parkinson’s disease in a therapeutic dance program. Disabil Rehabil 2017;39:1073–8. 10.1080/09638288.2016.1175037 27216230

[23] Fancourt D, Perkins R. Associations between singing to babies and symptoms of postnatal depression, wellbeing, self-esteem and mother-infant bond. Public Health 2017;145:149–52. 10.1016/j.puhe.2017.01.016 28359384

[24] Fancourt D, Perkins R. Effect of singing interventions on symptoms of postnatal depression: three-arm randomised controlled trial. Br J Psychiatry 2018;212:119–21. 10.1192/bjp.2017.29 29436333

[25] Fancourt D, Perkins R. The effects of mother–infant singing on emotional closeness, affect, anxiety, and stress hormones. Music Sci 2018;1:205920431774574. 10.1177/2059204317745746

[26] Fancourt D, Perkins R. Creative interventions for symptoms of postnatal depression: a process evaluation of implementation. Arts Health 2019;11:38–53. 10.1080/17533015.2017.1413398 31038038

[27] Houston S, McGill A. A mixed-methods study into ballet for people living with Parkinson's. Arts Health 2013;5:103–19. 10.1080/17533015.2012.745580 23805165 PMC3687249

[28] Cultural Commissioning Programme Case Study. English National Ballet Dance for Parkinson’s: Harnessing the power of dance to improve Health & Wellbeing, 2015.

[29] Fancourt D, Perkins R. Does attending community music interventions lead to changes in wider musical behaviours? the effect of mother–infant singing classes on musical behaviours amongst mothers with symptoms of postnatal depression. Psychol Music 2019;47:132–43. 10.1177/0305735617742197

[30] Perkins R, Yorke S, Fancourt D. How group singing facilitates recovery from the symptoms of postnatal depression: a comparative qualitative study. BMC Psychol 2018;6:41. 10.1186/s40359-018-0253-0 30119704 PMC6098577

[31] Estevao C, Bind R, Fancourt D, et al. SHAPER-PND trial: clinical effectiveness protocol of a community singing intervention for postnatal depression. BMJ Open 2021;11:e052133. 10.1136/bmjopen-2021-052133 PMC8601068 34789494

[32] Podlewska A, Davis R, Soukup T. Design of the PD-Ballet study: a randomised controlled single-blind hybrid type 2 clinical trial evaluating the effects of ballet dancing on motor and non-motor symptoms in Parkinson’s disease. 10.1186/s12906-023-04296-y PMC10792796 38233784

[33] Curran GM, Bauer M, Mittman B, et al. Effectiveness-implementation hybrid designs: combining elements of clinical effectiveness and implementation research to enhance public health impact. Med Care 2012;50:217–26. 10.1097/MLR.0b013e3182408812 22310560 PMC3731143

[34] King’s Cultural Community. Arts, health & wellbeing: King’s College London. Available: https://www.kcl.ac.uk/cultural/arts-health-wellbeing

[35] King’s Cultural Community. Arts-enhanced health education. Available: https://www.kcl.ac.uk/cultural/arts-health-wellbeing/education

[36] Grigoriadis S, Wilton AS, Kurdyak PA, et al. Perinatal suicide in Ontario, Canada: a 15-year population-based study. CMAJ 2017;189:1085–92. 10.1503/cmaj.170088 PMC5573543 28847780

[37] Slomian J, Honvo G, Emonts P, et al. Consequences of maternal postpartum depression: a systematic review of maternal and infant outcomes. Womens Health 2019;15:1745506519844044. 10.1177/1745506519844044 PMC6492376 31035856

[38] Pearlstein T, Howard M, Salisbury A, et al. Postpartum depression. Am J Obstet Gynecol 2009;200:357–64. 10.1016/j.ajog.2008.11.033 19318144 PMC3918890

[39] Alison C, Scott S. Pharmacologic and hormonal treatments for postpartum depression. Psychiatr Ann 2005;35:568–76.

[40] Becki H. NHS England’s Access and Waiting Times Programme Perinatal Mental Health, 2015 p. Available: http://www.swscn.org.uk/wp/wp-content/uploads/2015/12/-07-31-presentation-NHSE-perinatal-programme.pdf

[41] Yang G, Schmiel L, Zhou M. Economic Burden and Future Impact of Parkinson’s Disease Final Report. The Lewin Group, 2019.

[42] Parkinson’s UK. The incidence and prevalence of Parkinson’s in the UK. Parkinson’s UK, 2018.

[43] Kelberman MA, Vazey EM. New pharmacological approaches to treating non-motor symptoms of Parkinson's disease. Curr Pharmacol Rep 2016;2:253–61. 10.1007/s40495-016-0071-0 28534003 PMC5438167

[44] Zahoor I, Shafi A, Haq E. Pharmacological Treatment of Parkinson’s Disease. Parkinson’s Disease: Pathogenesis and Clinical Aspects. Brisbane Codon Publications, 2018.

[45] Barnish MS, Barran SM. A systematic review of active group-based dance, singing, music therapy and theatrical interventions for quality of life, functional communication, speech, motor function and cognitive status in people with Parkinson's disease. BMC Neurol 2020;20:371. 10.1186/s12883-020-01938-3 33038925 PMC7547481

[46] McGill A, Houston S, Lee RYW. Effects of a ballet-based dance intervention on gait variability and balance confidence of people with Parkinson’s. Arts Health 2019;11:133–46. 10.1080/17533015.2018.1443947 31038440

[47] Craig P, Dieppe P, Macintyre S, et al. Developing and evaluating complex interventions: the new medical Research Council guidance. BMJ 2008;337:a1655. 10.1136/bmj.a1655 18824488 PMC2769032

[48] Craig P, Dieppe P, Macintyre S, et al. Developing and evaluating complex interventions: the new medical Research Council guidance. Int J Nurs Stud 2013;50:587–92. 10.1016/j.ijnurstu.2012.09.010 23159157

[49] Hull L, Goulding L, Khadjesari Z, et al. Designing high-quality implementation research: development, application, feasibility and preliminary evaluation of the implementation science research development (ImpRes) tool and guide. Implement Sci 2019;14:80. 10.1186/s13012-019-0897-z 31412887 PMC6693182

[50] Soukup T, Hull L, Smith EL, et al. Effectiveness-implementation hybrid type 2 trial evaluating two psychoeducational programmes for severe hypoglycaemia in type 1 diabetes: implementation study protocol. BMJ Open 2019;9:303–70. 10.1136/bmjopen-2019-030370 PMC6886982 31727650

[51] Michie S, van Stralen MM, West R. The behaviour change wheel: a new method for characterising and designing behaviour change interventions. Implement Sci 2011;6:42. 10.1186/1748-5908-6-42 21513547 PMC3096582

[52] Consolidated framework for implementation research, 2021. Available: https://cfirguide.org/

[53] Damschroder LJ, Aron DC, Keith RE, et al. Fostering implementation of health services research findings into practice: a consolidated framework for advancing implementation science. Implement Sci 2009;4:50. 10.1186/1748-5908-4-50 19664226 PMC2736161

[54] Powell BJ, Waltz TJ, Chinman MJ, et al. A refined compilation of implementation strategies: results from the expert recommendations for implementing change (ERIC) project. Implement Sci 2015;10:21. 10.1186/s13012-015-0209-1 25889199 PMC4328074

[55] Waltz TJ, Powell BJ, Matthieu MM, et al. Use of concept mapping to characterize relationships among implementation strategies and assess their feasibility and importance: results from the expert recommendations for implementing change (ERIC) study. Implement Sci 2015;10:109. 10.1186/s13012-015-0295-0 26249843 PMC4527340

[56] RE-AIM, 2021. Available: https://www.re-aim.org/

[57] Glasgow RE, Vogt TM, Boles SM. Evaluating the public health impact of health promotion interventions: the RE-AIM framework. Am J Public Health 1999;89:1322–7. 10.2105/ajph.89.9.1322 10474547 PMC1508772

[58] Smith JD, Li DH, Rafferty MR. The implementation research logic model: a method for planning, executing, reporting, and synthesizing implementation projects. Implement Sci 2020;15:84. 10.1186/s13012-020-01041-8 32988389 PMC7523057

[59] Proctor E, Silmere H, Raghavan R, et al. Outcomes for implementation research: conceptual distinctions, measurement challenges, and research agenda. Adm Policy Ment Health 2011;38:65–76. 10.1007/s10488-010-0319-7 20957426 PMC3068522

[60] Weiner BJ, Lewis CC, Stanick C, et al. Psychometric assessment of three newly developed implementation outcome measures. Implement Sci 2017;12:108. 10.1186/s13012-017-0635-3 28851459 PMC5576104

[61] Soukup THL, Smith EL, et al, PWD Group. The role, value and challenges of stakeholder involvement in implementation research. BMJ Q&S.

[62] Soukup T, Hull L, Healey A. Interview topic guides exploring implementation outcomes across stakeholder groups (version 2) Zenodo 2020.

[63] Herdman M, Gudex C, Lloyd A, et al. Development and preliminary testing of the new five-level version of EQ-5D (EQ-5D-5L). Qual Life Res 2011;20:1727–36. 10.1007/s11136-011-9903-x 21479777 PMC3220807

[64] et alFinch TG, May M;, Mair CR;. NoMad: implementation measure based on normalization process theory, 2015. Available: http://www.normalizationprocess.org

[65] Cox JL, Holden JM, Sagovsky R. Detection of postnatal depression. development of the 10-item Edinburgh postnatal depression scale. Br J Psychiatry 1987;150:782–6. 10.1192/bjp.150.6.782 3651732

[66] Martinez-Martin P, Chaudhuri KR, Rojo-Abuin JM, et al. Assessing the non-motor symptoms of Parkinson’s disease: MDS-UPDRS and NMS Scale. Eur J Neurol 2015;22:37–43. 10.1111/ene.12165 23607783

[67] De Stavola BL, Daniel RM, Ploubidis GB, et al. Mediation analysis with intermediate confounding: structural equation modeling viewed through the causal inference lens. Am J Epidemiol 2015;181:64–80. 10.1093/aje/kwu239 25504026 PMC4383385

[68] RK. Principles and practice of structural equation modeling. 3rd ed. New York: Guilford press, 2011.

[69] Cronbach LJ. Coefficient alpha and the internal structure of tests. Psychometrika 1951;16:297–334. 10.1007/BF02310555

[70] DeVellis R. Scale development: theory and applications: SAGE publications, 2016.

[71] Muthén B, du Toit S, Spisic D. Robust inference using weighted least squares and quadratic estimating equations in latent variable modeling with categorical and continuous outcomes. Psychometrika 1997;75:40–5.

[72] Bentler PM. Comparative fit indexes in structural models. Psychol Bull 1990;107:238–46. 10.1037/0033-2909.107.2.238 2320703

[73] Tucker LR, Lewis C. A reliability coefficient for maximum likelihood factor analysis. Psychometrika 1973;38:1–10. 10.1007/BF02291170

[74] Steiger JH. Statistically-Based tests for the number of common factors, 1980.

[75] Lt H, Bentler PM. Cutoff criteria for fit indexes in covariance structure analysis: conventional criteria versus new alternatives. Struct Equ Modeling 1999;6:1–55.

[76] Muthén LK, Muthén BO. Mplus User’s Guide. 5th ed. Los Angeles, CA: Muthén & Muthén: 1998–2007.

[77] Harden SM, Smith ML, Ory MG, et al. Re-aim in clinical, community, and corporate settings: perspectives, strategies, and recommendations to enhance public health impact. Front Public Health 2018;6:71. 10.3389/fpubh.2018.00071 29623270 PMC5874302

[78] Damschroder LJ, Hagedorn HJ. A guiding framework and approach for implementation research in substance use disorders treatment. Psychol Addict Behav 2011;25:194–205. 10.1037/a0022284 21443291

[79] Kirk MA, Kelley C, Yankey N, et al. A systematic review of the use of the consolidated framework for implementation research. Implement Sci 2016;11:72. 10.1186/s13012-016-0437-z 27189233 PMC4869309

