# Supplementary material for: Study protocol: randomised controlled hybrid type 2 trial evaluating the scale-up of two arts interventions for postnatal depression and Parkinson’s disease
Source: BMJ Open. 2022 Feb 1;12(2):e055691. doi: 10.1136/bmjopen-2021-055691 (PMC8808453; doi:10.1136/bmjopen-2021-055691)
Supplement: Supplementary data [file bmjopen-2021-055691supp001.pdf]

IRAS number: 278445

Participant ICF Version 1.8 20-July-21

KING'S College LONDON

NHS South London and Maudsley NHS Foundation Trust

CONSENT FORM FOR PARTICIPANTS IN RESEARCH STUDIES

Please complete this form after you have read the Information Sheet and/or listened to an explanation about the research.

<u>Title of Study:</u> SHAPER-PND: Community singing interventions for postnatal depression: a hybrid type II effectiveness-implementation trial

Thank you for considering taking part in this research. One of the researchers from the research team will explain the project to you before you agree to take part. If you have any questions arising from the Information Sheet or explanation already given to you, please ask the researcher before you decide whether you should join in. You will be given a copy of this Consent Form to keep and refer to
at any time.

Your information may be subject to review by responsible individuals from the research team for screening, monitoring and audit purposes.

Confidentiality will be maintained and you will not be identified in any research outputs.

	Yes	No
**I confirm that I understand that by ticking/initialling a 'Yes' box, I am consenting to being involved in this element of the study. I confirm that I understand that by ticking/initialling a 'No' box, I DO NOT consent to being involved in this element of the study.**	☐	☐
1. I confirm that I have read and understood the information sheet dated [30-Jul-2021, version 2.1] for the above study. I have had the opportunity to consider the information and asked questions which have been answered to my satisfaction.	☐	☐
2. I consent voluntarily for myself and for my baby to be a participant in this study and understand that I can withdraw from the study at any time, without having to give a reason.	☐	☐
3. I consent to the processing of my personal information for the purposes explained to me in the Information Sheet. I understand that such information will be handled confidentially, in accordance with the terms of the General Data Protection Regulation.	☐	☐
4. I agree that the research team may use my data for future research within and outside the EU and understand that any such use of identifiable data would be reviewed and approved by a research ethics committee. In such cases, as with this project, data will not be identifiable in any report.	☐	☐
5. I agree to be contacted in the future by King's College London researchers who would like to invite me to participate in follow up studies to this project, or in future studies of a similar nature.	☐	☐

Soukup T, *et al. BMJ Open* 2022; 12:e055691. doi: 10.1136/bmjopen-2021-055691

Supplemental material BMJ Publishing Group Limited (BMJ) disclaims all liability and responsibility arising from any reliance placed on this supplemental material which has been supplied by the author(s) BMJ Open

6. I agree to provide details of my GP and understand that if any safeguarding issues arise, my GP might be contacted to discuss the best way to support me and my baby. ☐ ☐

7. I agree to record short videos being taken of play interaction in a comfortable setting between my baby and me. This recording aims to look at how me and my baby interact with each other and the information obtained can be used for our research. ☐ ☐

8. I agree to my views being shared in focus groups and subsequent interviews to be recorded (in audio and/or video format) for data collection. The researchers may use a transcription service to transcribe the recordings into a different format. I understand that my identity will stay anonymous and I will not be identifiable in the published data or materials. (optional) ☐ ☐

9. I agree to provide biological samples (saliva) throughout the study in accordance to the study protocol. (optional) ☐ ☐

10. I agree to provide biological samples (hair) throughout the study in accordance to the study protocol. (optional) ☐ ☐

11. My biological samples may be used for future studies and my data will remain anonymous. (optional) ☐ ☐

12. I agree to provide biological samples (saliva only) of my baby in accordance to the study protocol. (optional) ☐ ☐

13. The biological samples of my baby may be used for future studies and the data will remain anonymous. (optional) ☐ ☐

14. I agree to be contacted by implementation science researchers to provide my views on the study. I understand that my views may be video and audio recorded and published but I will not be identifiable in any of the research outputs. (optional) ☐ ☐

15. I do not wish to participate in the study, but I agree to be contacted by implementation science researchers for a brief interview, to explain why. (optional) ☐ ☐

Note that you must consent to points 1-7 in order to be eligible for the study. Points 8-14 are not mandatory but are still an integral part of the study. If you do not wish to participate in the study but consent to being contacted to tell us why (optional), just tick point 15.

Participant:

____________________	____________________	____________________
Your Name	Date	Signature

Researcher:

____________________	____________________	____________________
Name of Researcher	Date	Signature

Soukup T, *et al. BMJ Open* 2022; 12:e055691. doi: 10.1136/bmjopen-2021-055691
