# Supplementary material for: Study protocol: randomised controlled hybrid type 2 trial evaluating the scale-up of two arts interventions for postnatal depression and Parkinson’s disease
Source: BMJ Open. 2022 Feb 1;12(2):e055691. doi: 10.1136/bmjopen-2021-055691 (PMC8808453; doi:10.1136/bmjopen-2021-055691)
Supplement: Author's manuscript [file bmjopen-2021-055691.draft_revisions.pdf]

BMJ Open is committed to open peer review. As part of this commitment we make the peer review history of every article we publish publicly available.

When an article is published we post the peer reviewers' comments and the authors' responses online. We also post the versions of the paper that were used during peer review. These are the versions that the peer review comments apply to.

The versions of the paper that follow are the versions that were submitted during the peer review process. They are not the versions of record or the final published versions. They should not be cited or distributed as the published version of this manuscript.

BMJ Open is an open access journal and the full, final, typeset and author-corrected version of record of the manuscript is available on our site with no access controls, subscription charges or pay-per-view fees (http://bmjopen.bmj.com).

If you have any questions on BMJ Open's open peer review process please email info.bmjopen@bmj.com

BMJ Open

Study protocol: Randomised controlled hybrid type 2 trial evaluating the scale-up of two arts interventions for Postnatal depression and Parkinson's disease

Journal:	*BMJ Open*
Manuscript ID	bmjopen-2021-055691
Article Type:	Protocol
Date Submitted by the Author:	21-Jul-2021

Complete List of Authors:	Soukup, Tayana ; King's College London, Centre Implementation Science; King's College London<br>Davis, Rachel; Institute of Psychiatry Psychology and Neuroscience Division of Psychology and Systems Sciences<br>Baldellou Lopez, Maria<br>Healey, Andy; King's College London, IOPPN<br>Estevao, Carolina; King's College London, nstitute of Psychiatry, Psychology & Neuroscience<br>Fancourt, Daisy; University College London, Department of Behavioural Science and Health<br>Dazzan, Paola; King's College London<br>Pariante, Carmine; King's College London, Psychological Medicine<br>Dye, Hannah; The Clarence Centre<br>Osborn, Tim; The Clarence Centre<br>Bind, Rebecca; King's College London, Department of Psychological Medicine<br>Sawyer, Kristi; King's College London, Department of Psychological Medicine<br>Rebecchini, Lavinia; King's College London, Department of Psychological Medicine<br>Hazelgrove, Katie; King's College London, Department of Psychological Medicine<br>Burton, Alexandra; University College London, Division of Psychiatry<br>Manoharan, Manonmani; South London and Maudsley NHS Foundation Trust, lewisham Perinatal team<br>Perkins, Rosie; Royal College of Music, Centre for Performance Science; Imperial College London, Faculty of Medicine<br>Podlewska, Aleksandra<br>Chaudhuri, Ray; King\'s College London<br>Derbyshire-Fox, Fleur<br>Hartley, Alison<br>Woods, Anthony; King's College London, Department of Psychological Medicine<br>Crane, Nikki; King's College London<br>Bakolis, Ioannis; King's College London,<br>Sevdalis, Nick; King's College London,

Keywords:	Maternal medicine < OBSTETRICS, Parkinson-s disease < NEUROLOGY,

	HEALTH ECONOMICS, HEALTH SERVICES ADMINISTRATION & MANAGEMENT

SCHOLARONE™ Manuscripts

I, the Submitting Author has the right to grant and does grant on behalf of all authors of the Work (as defined in the below author licence), an exclusive licence and/or a non-exclusive licence for contributions from authors who are: i) UK Crown employees; ii) where BMJ has agreed a CC-BY licence shall apply, and/or iii) in accordance with the terms applicable for US Federal Government officers or employees acting as part of their official duties; on a worldwide, perpetual, irrevocable, royalty-free basis to BMJ Publishing Group Ltd ("BMJ") its licensees and where the relevant Journal is co-owned by BMJ to the co-owners of the Journal, to publish the Work in this journal and any other BMJ products and to exploit all rights, as set out in our licence.

The Submitting Author accepts and understands that any supply made under these terms is made by BMJ to the Submitting Author unless you are acting as an employee on behalf of your employer or a postgraduate student of an affiliated institution which is paying any applicable article publishing charge ("APC") for Open Access articles. Where the Submitting Author wishes to make the Work available on an Open Access basis (and intends to pay the relevant APC), the terms of reuse of such Open Access shall be governed by a Creative Commons licence – details of these licences and which Creative Commons licence will apply to this Work are set out in our licence referred to above.

Other than as permitted in any relevant BMJ Author's Self Archiving Policies, I confirm this Work has not been accepted for publication elsewhere, is not being considered for publication elsewhere and does not duplicate material already published. I confirm all authors consent to publication of this Work and authorise the granting of this licence.

For peer review only

Study protocol: Randomised controlled hybrid type 2 trial evaluating the scale-up of two arts interventions for Postnatal depression and Parkinson's disease

Tayana Soukup,[1]✉* Rachel Davis,[1]✉* Maria Baldellou Lopez,[1] Andy Healey,[1] Carolina Estevao[3], Daisy Fancourt[5], Paola Dazzan[3], Carmine Pariante[3], Hannah Dye[4], Tim Osborn[4], Rebecca Bind[3], Kristi Sawyer[3], Lavinia Rebecchini[3], Katie Hazelgrove[3], Alexandra Burton[5], Manonmani Manoharan[6], Rosie Perkins[7,8], Aleksandra Podlewska[9], Ray Chaudhuri[9], Fleur Derbyshire-Fox[10], Alison Hartley[10], Anthony Woods[3], Nikki Crane[3], Ioannis Bakolis,[1,2]** Nick Sevdalis[1]**.

*These two authors have contributed equally and share first authorship.

** These two authors have contributed equally and share senior authorship.

Author affiliations

[1] Centre for Implementation Science, King's College London

[2] Department of Biostatistics and Health Informatics, King's College London

[3] Department of Psychological Medicine, King's College London

[4] Breathe Arts Health Research, London.

[5] Department of Behavioural Science and Health, University College London.

[6] South London and Maudsley NHS Foundation Trust, London.

[7] Centre for Performance Science, Royal College of Music, London.

[8] Faculty of Medicine, Imperial College London

[9] Department of Basic and Clinical Neuroscience, King's College London

[10] English National Ballet, London

Full address for correspondence

✉Dr Rachel Davis, Centre for Implementation Science, Health Service & Population Research Department, Institute of Psychiatry, Psychology and Neuroscience King's College London, 16 De Crespigny Park, London SE5 8AF. Email: rachel.davis@kcl.ac.uk

For peer review only - http://bmjopen.bmj.com/site/about/guidelines.xhtml

Abstract

Introduction Research on the benefits of 'arts' interventions to improve individuals' physical, social and psychological wellbeing is growing, but evidence on implementation and scale-up into health and social care systems is lacking. This protocol reports the SHAPER-Implement programme (Scale-up of Health-Arts Programmes Effectiveness-Implementation Research), aimed at studying the impact, implementation and scale-up of: Melodies for Mums (M4M), a singing intervention for postnatal depression; and PD-Ballet a dance intervention for Parkinson's disease. We examine how they could be embedded in clinical pathways to ensure their longer-term sustainability.

Methods and analysis A randomized two-arm effectiveness-implementation hybrid type 2 trial design will be used across M4M/PD-Ballet. We will assess the implementation in both study arms (intervention versus control), and the cost-effectiveness of implementation. The design and measures, informed by literature and previous research by the study team, were refined through stakeholder engagement. Participants (400 in M4M; 160 in PD-Ballet) will be recruited to the intervention or control group (2:1 ratio). Further implementation data will be collected from stakeholders involved in referring to, delivering, or supporting M4M/PD-Ballet (N = 25-30 for each intervention).

A mixed-methods approach (surveys and semi-structured interviews) will be employed. 'Acceptability' (measured by the 'Acceptability Intervention Measure') is the primary implementation endpoint for M4M/PD-Ballet. Relationships between clinical and implementation outcomes, implementation strategies (e.g. training) and outcomes will be explored using generalised linear mixed models. Qualitative data will assess factors affecting the acceptability, feasibility, and appropriateness of M4M/PD-Ballet, implementation strategies and longer-term sustainability. Costs associated with implementation and future scale-up will be estimated.

Ethics and dissemination SHAPER-PND (the M4M trial) and SHAPER-PD (the PD trial) are approved by the West London and GTAC (20/PR/0813) and the HRA and Health and Care Research Wales (REC Reference: 20/WA/0261) Research Ethics Committees. Study findings will be disseminated through scientific peer-reviewed journals and scientific conferences.

Trial registration numbers: Both trials are registered with NIH US National Library of Medicine, ClinicalTrials.gov. The trial registration numbers, URLs of registry records, and dates of registration are:

For peer review only - http://bmjopen.bmj.com/site/about/guidelines.xhtml

For peer review only

- PD-Ballet: URLNCT04719468 https://eur03.safelinks.protection.outlook.com/?url=https%3A%2F%2Fwww.clinicaltrials.gov%2Fct2%2Fshow%2FNCT04719468%3Fterm%3DNCT04719468%26draw%3D2%26rank%3D1&data=04%7C01%7Crachel.davis%40kcl.ac.uk%7C11a7c5142782437919f808d903111449%7C8370cf1416f34c16b83c724071654356%7C0%7C0%7C637544194261659488%7CUnknown%7CTWFpbGZsb3d8eyJWIjoiMC4wLjAwMDAiLCJQIjoiV2luMzIiLCJBTiI6Ik1haWwiLCJXVCI6Mn0%3D%7C1000&sdata=jNjPRC3e9oY6oGCeNQ0zE%2FusMlZqUd2r4bFLTvHRDO0%3D&reserved=0 (date of registration – January 22st 2021)

- Melodies for Mums: NCT04834622 https://clinicaltrials.gov/ct2/show/NCT04834622?term=shaper-pnd&draw=2&rank=1 (date of registration – April 8th 2021)

Key words: implementation-effectiveness; hybrid type 2 trial; Parkinson's disease; Postnatal depression; implementation science; scaling up; sustainability; arts interventions.

Word Count: 4000 words.

Strengths and limitations of the study

- Largest known study of its kind, comprising multidisciplinary implementation and evaluation teams, with consistent stakeholder engagement embedded throughout
- Large scale psychometric validation of newly developed implementation measures
- Novel exploration of potential associations between quantitative implementation data and clinical measures
- Provides an example of how large-scale hybrid studies can be conducted within community settings and a synergistic methodology with broad applicability
- While every attempt will be made to be as inclusive as possible with our sample of trial participants, further research on the wider generalisability of our findings, would be of benefit.

Introduction

The use of arts interventions (i.e. 'creative methods of expression such as drama, music and visual arts'[1]) to improve health and social care outcomes and reduce service utilisation costs is an

For peer review only - http://bmjopen.bmj.com/site/about/guidelines.xhtml

For peer review only

internationally growing area of research[1-4]. In 2017, the United Kingdom's All-Party Parliamentary Group on Arts, Health and Wellbeing (APPGAHW) published a report on the benefits of arts interventions, alongside ten stakeholder-led recommendations (from patients, health and social care professionals, artists, academics, charities, policy-makers and parliamentarians) on facilitating the implementation and scale-up of 'arts' into health and social care systems nationally[1]. Two years on, the World Health Organisation's scoping review of the global academic literature (2000-2019)[2] identified over 900 publications, including 200 reviews, systematic reviews, meta-analyses and meta-syntheses covering over 3000 studies, and 700 additional studies. Taken the evidence collectively, arts interventions are a clinically effective method to help treat a plethora of physical, social and psychological problems across the lifespan[1-4].

Despite the promising evidence, progress on successfully embedding art interventions in health and social care systems has been slow. Presently, many 'arts' are delivered in small geographic or healthcare pockets, operating at the fringe of the care sector rather than receiving mainstream funding[1, 5]. While lack of sustainable funding, strong partnerships with commissioners and clear referral pathways partly account for this[1, 5] research is required to establish cost-effective, scalable solutions so that the full benefits to the wider population can be reached.

The current protocol reports the design and evaluation of the 'Scale-up of Health-Arts Programmes Effectiveness-Implementation Research' (SHAPER-Implement) – part of a larger programme (referred to as 'SHAPER'[6]) aimed at investigating ways to implement and deliver arts interventions at scale. We focus on two different health conditions: postnatal depression (PND) and Parkinson's disease (PD). These represent a significant fiscal and public health burden and pose considerable affliction on the individuals affected (and, where applicable, their carers)[7-14]. While pharmacological treatments can be effective for controlling/alleviating symptoms[7,9], they are fraught with challenges: for PND, it is poor uptake and adherence[15-18], while for PD, it is overemphasis on treating the motor symptoms at the expense of non-motor functioning[19-21].

Melodies for Mums (M4M) (for mums with PD) and PD-Ballet (for individuals with PD) are two alternative (or conjunctive) approaches to symptom management, that have been piloted with promising results[22-30]. Both have already been implemented across certain locations in London, UK, but are not being delivered at scale, thus only reach a fraction of eligible individuals.

We plan to scale-up M4M/PD-Ballet and examine how we can embed them into clinical pathways so that a greater number of individuals can benefit. Our ambition is to be as inclusive as possible,

For peer review only - http://bmjopen.bmj.com/site/about/guidelines.xhtml

reaching out to individuals who may not be undergoing treatment for their condition (as well as those that are), and ultimately for Clinical Commissioning Groups (e.g. the "payers" in the National Health Service, UK) to commission the interventions so they can be delivered in a sustainable way beyond the end of our research.

The entire SHAPER programme centres on tripartite objectives. Due to the complexity of the study design, this protocol reports the implementation effectiveness evaluation (i.e. the SHAPER-Implement programme) of M4M/PD-Ballet (objectives 2 and 3, described below). The clinical effectiveness evaluation (objective 1) is reported in parallel protocols[31, 32].

Objective 1: to assess the *clinical effectiveness* of M4M/PD-Ballet – described in detail in the clinical protocols[31, 32]

Objective 2: to examine the *implementation effectiveness* of M4M/PD-Ballet, including uptake, adoption, perceived acceptability, appropriateness, feasibility, fidelity, unintended consequences, and sustainability, and the impact of established implementation strategies (e.g., training in the delivery of M4M/PD-Ballet) on implementation effectiveness;

Objective 3: to assess *implementation costs and cost effectiveness*, including costs associated with implementing M4M/PD-Ballet into existing care pathways, health service, partner organisations and commissioning, costs to service users attending M4M/PD-Ballet versus the benefit in terms of quality-adjusted years of life lived (QALYs), and the impact of M4M/PD-Ballet when delivered at scale, on the wider utilisation of health care and other services.

Methods and analysis

Design

SHAPER-Implement is a two-arm effectiveness-implementation hybrid type 2[33] trial of M4M/PD-Ballet. Randomisation will be single-blinded (assessments performed by a blinded rater) and in a 2:1 ratio. M4M participants will receive the singing programme (intervention) or be encouraged to attend non-music classes in the community or online (control). PD-Ballet participants will receive a dance programme (dance-based training and a post-session Tea-and-Biscuit social time) or follow the standard treatment per the local pathway (control) and attend the post-training 'Tea-and-Biscuit' gatherings via an online platform.

For peer review only - http://bmjopen.bmj.com/site/about/guidelines.xhtml

Table 1 details how M4M/PD-Ballet meet the criteria proposed by Curran et al[33] for a hybrid type 2 trial. Contextual constraints meant it was not feasible to randomise the implementation side of the trial (i.e. allocate participants to different implementation strategies): but we will examine the effectiveness of the implementation strategies used to deploy M4M/PD-Ballet.

Setting

SHAPER-Implement is a multisite, multidisciplinary, community-based study in London, UK. Funded by the Wellcome Trust, it is a collaboration between the Centre for Implementation Science, King's College London; King's Health Partners; the Department for Behavioural Science and Health and the Institute of Mental Health, University College London, and two award-winning arts organisations: Breathe Arts Health Research and English National Ballet (ENB). King's holds a long-established commitment to embedding arts, health and wellbeing in education and research[34, 35] and was the research partner for landmark publications in the area[1].

The health conditions

PND is a serious and the most common perinatal mental health condition, affecting 10-20% of women in pregnancy and after birth[11]. If left untreated the impact on women and their families can be devastating[11, 36, 37] with symptoms including fatigue, anhedonia, insomnia and irritability and thoughts of suicide[7, 8, 37,38]. Therapy (e.g. cognitive-behavioural therapy) or medication can be prescribed to help treat PND but progress can be slow, may involve long wait times for treatment (for therapy) or prolonged use of medication[39-41]- thus other options for symptom management need to be explored.

PD is one of the world's fastest growing chronic neurodegenerative disorders, with those aged over 70 being particularly vulnerable[14, 42, 43]. It affects over 145,000 people in the UK alone[43] with prevalence rates expected to rise by around 18% between 2018 and 2025 (to over 168,000), and doubling by 2065[10, 43]. Symptoms relate to motor (e.g. tremor, bradykinesia, freezing of gait) and non-motor (e.g. sleep disturbance, drooling, cognitive decline)[9, 10] functioning. Currently, there is

For peer review only - http://bmjopen.bmj.com/site/about/guidelines.xhtml

no cure for PD – and the disease has a progressive course[9, 10]. Pharmacological treatments can alleviate symptoms and improve quality of life but are largely aimed at addressing motor functioning, leaving the non-motor symptoms often unrecognised or under-treated[10, 44, 45]. Alternative non-pharmacotherapies are necessary to slow down disease progression[10, 44, 45].

The interventions

Drawing from Curran et als' 'hybrid' framework[33] through an intensive four-month stakeholder engagement with artists, researchers, clinicians, and commissioners, we considered five elements critical to assessing the relevance of the interventions:

- **Suitability** – M4M/PD-Ballet address a need within the health sector – there are growing patient populations with the conditions and lack of effective current service provision.
- **Quality and face validity** – M4M/PD-Ballet are 'high-quality' interventions with carefully designed and tailored activities, developed in partnership between centres of academic excellence and renowned arts organisations.
- **Inclusivity** – M4M/PD-Ballet are 'all-inclusive'. They have good uptake, not only with those already engaged in the arts. They also appear to 'reach-out' to individuals that are disengaged in other forms of treatment for PND/PD (e.g., pharmacological approaches).
- **Effectiveness** – there is evidence to show M4M/PD-Ballet improves symptoms and quality of life and can achieve better adherence than pharmacological approaches[23-26,28-30].
- **Scalability** – M4M/PD-Ballet are not overly complex, thus have the potential to be scaled-up, embedded in clinical pathways and commissioned by the health sector[6].

M4M[6,31] is delivered in partnership with Breathe Arts Health Research (https://breatheahr.org/melodies-for-mums/). A Breathe-trained artist (the workshop leader) and support assistant deliver ten weekly singing sessions to a group of mums with PND and their babies. Classes start with welcome songs, followed by music activities, ranging from short vocal exercises and simple lullabies to longer songs that attendees learn gradually over the weeks. Songs can be relaxing, with mothers encouraged to hug/stroke their babies as they sing, or energetic, with mothers standing and moving with their babies. Instruments (e.g. guitars and ukuleles) are used by the workshop leader, accompanied by simpler instruments for mothers/babies to use. Mothers are encouraged to write their own songs, developing lyrics together relating to their babies or

For peer review only - http://bmjopen.bmj.com/site/about/guidelines.xhtml

experiences of motherhood[6, 31]. Recordings of the singing sessions are made available to attendees at the end.

M4M has been subject to a three-arm randomised controlled trial (RCT) of 134 mothers and a preliminary process evaluation. Significantly faster improvements in symptoms for mothers with moderate-severe PND (measured by the Edinburgh Postnatal Depression Scale (EPDS)), than mothers in usual care[24] were observed. Levels of depression consistently declined - by week 6, 65% of mothers no longer had an EPDS ≥ 13, (i.e., indicating no more than mild depression); increasing to 73% by week 10[24]. Increase in the frequency of mothers singing to their babies outside the classes, perceived mother-infant closeness and a greater decrease in cortisol levels when compared to social play were also reported[23-25]. The process evaluation showed that M4M reached the correct target demographic, was delivered with a high level of fidelity and programme satisfaction was high - 88% of mothers agreed the classes were well tailored, and 100% would recommend M4M to another mother[26,29,30]. Several challenges, however, were highlighted by mothers, workshop leaders and the project coordinator (e.g., timing sessions with babies' routines) – whilst this did not hinder the continuation of M4M, it nonetheless suggests that implementation of M4M could be improved[26].

PD-Ballet[6,32] is based on the ENB's pre-existing 'Dance for Parkinson' (DfP) programme (referred to as PD-Ballet for the SHAPER research) and delivered in partnership with the ENB (https://www.ballet.org.uk/project/dance-for-parkinsons/). Led by an ENB trained DfP Associate Artist and Associate Musician who deliver twelve weekly sessions to individuals with PD (carers/relatives can also attend), sessions comprise live music, dance and vocal exercises. Each session comprises seventy-five minutes of activity, followed by social time and refreshments (up to one hour) so that participants can get to know fellow attendees and form social networks. Content, inspired by the ENB's classical and contemporary works, provides a framework for participants to explore narrative, themes, concepts, and music to promote freedom of expression. A performance sequence is developed at the end of PD-Ballet, combining all elements of the programme. Dance material is adapted to be inclusive (catering for differing levels of mobility) so that everyone can participate fully. For PD-Ballet content will be further developed for the three distinct stages of motor advancement (mild/moderate/severe) – something which has not been done before within the existing DfP programme. Prior to the programme, individuals attend an introductory session with their assigned group and are given the opportunity to attend a ballet performance and a behind the-scenes event[6, 32].

For peer review only - http://bmjopen.bmj.com/site/about/guidelines.xhtml

Initial testing of PD-Ballet in London, and its regional affiliated hub partners, has proven to be replicable, resulting in it being trialled and established in four other locations in the UK (Oxford, Cardiff, Ipswich and Liverpool). An independent evaluation led by the University of Roehampton (on scaling-up the programme, experiences and benefits of participation across four years and the effects on the body, daily activities, and social participation) reported high levels of perceived value from an emotional, social and artistic perspective[28]. Additionally, PD-Ballet can decrease social isolation and improve quality of life[22], with participants highly motivated and viewing PD-Ballet as an important part of their lives[27]. However, very few RCTs have investigated the benefits of 'dance' in both motor and non-motor symptoms. There has been no published research on ballet as an intervention for PD, pointing to the need for this research.

SHAPER-Implement will build on previous research - conducting a full RCT (for PD-Ballet), a follow-up RCT scaled to a larger number of mothers (for M4M), and a process evaluation and examination of the implementation and potential cost-effectiveness of delivery at scale (for both) (see Table 2 for further details on M4M/PD-Ballet).

Implementation and adaption - COVID-19 pandemic

As a result of the first national lockdown in the United Kingdom on 23rd March 2020 and the guidelines that followed, M4M/PD-Ballet were adapted to be delivered online and preliminary research into the feasibility of using an online platform is underway.

Theoretical underpinning

The Medical Research Council's (MRC) framework for evaluating complex interventions[46, 47] informed the study design for intervention development, implementation and evaluation processes.

The 'Implementation Science Research Development' (ImpRes) tool[48,] allowed us to operationalise the MRC guidance into the overall design of SHAPER-Implement, along with the research team's knowledge and experience of conducting hybrid trials[49]. We also tailored intervention delivery using the COM-B model[50] by considering factors that could affect individuals' capability, opportunity and motivation to engage with M4M/PD-Ballet.

For peer review only - http://bmjopen.bmj.com/site/about/guidelines.xhtml

The Consolidated Framework for Implementation Research (CFIR)[51, 52] will be used to assess factors affecting the implementation M4M/PD-Ballet. The 'Expert Recommendations for Implementing Change' (ERIC) project's compendium of implementation strategies[53, 54] will guide the identification of implementation strategies that could overcome some of the factors affecting implementation.

The Reach Effectiveness Adoption Implementation Maintenance (RE-AIM) framework[55, 56], which comprises domains on 'implementation' and 'effectiveness' and is suited to address challenges of blended effectiveness-implementation designs, guided the selection of implementation measures alongside Proctor et al[44] taxonomy of implementation outcomes.

Participants

For M4M, 400 mothers (with their babies) will be randomised 2:1 to the intervention (N=270) versus control (N=130). Mothers over 18 years of age with symptoms of PND (defined as a diagnosis of major depressive disorder according to the Structured Clinical Interview for DSM-IV Disorders and a score of ≥ 10 on the EPDS) and a baby 0-9 months old, will be eligible. Mothers with an EPDS score of < 10 will be excluded and signposted to other support services within the community (e.g. mother-baby groups) but invited to re-screen for future cycles of the intervention (in case their symptoms change).

For PD-Ballet, 160 individuals will be recruited: 2:1 to the intervention (N=107) versus control (N=53). Participants will be 18 years old or over with a diagnosis of idiopathic PD according to the UK PD Brain Bank criteria and Hoehn Yarhr stages I-V. Individuals with a diagnosis of other parkinsonism or an indication of dementia through a score of ≤21 on the Montreal Cognitive Assessment (MoCA) will be excluded.

Individuals (for both M4M/PD-Ballet) will also be excluded if they are unable to: understand English; give informed consent; access and attend the singing/dance sessions online (due to COVID-19) through an internet-connected device (e.g. mobile phone, computer), or face-to-face (government guidelines permitting).

For peer review only - http://bmjopen.bmj.com/site/about/guidelines.xhtml

Individuals who decline participation or withdraw from M4M/PD-Ballet will be asked (providing they are willing) to provide their reason(s), so that barriers to uptake (from ‘decliners’) or continued participation (from ‘withdrawers’) can be identified and addressed.

SHAPER-Implement will also collect data on stakeholders’ attitudes and experiences of M4M/PD-Ballet (N=25-30, respectively) so that challenges relating to intervention delivery can be reviewed and overcome.

A description of power and sample size calculations for M4M/PD-Ballet can be found in the corresponding clinical protocols[31, 32].

Patient and public involvement

We are engaging with members of the public and patients throughout SHAPER-Implement and at every stage of the research process. We have formed advisory groups comprising individuals who have already completed the PD-Ballet and M4M programme. To date, these individuals have provided feedback on (but not limited to) our study design, data collection measures, recruitment strategies and methods for disseminating findings.

Wider stakeholder engagement

In addition to patients/the public, engagement with other stakeholders is also critical to the success and scalability of M4M/PD-Ballet. We will hold regular meetings with: ‘deliverers’ – artists/staff involved in delivering M4M/PD-Ballet; ‘referrers’ – those who refer individuals to M4M/PD-Ballet, (e.g. general practitioners or neurologists (PD-Ballet); psychiatrists, health visitors and midwives (M4M) and; ‘supporters’ – additional staff who support the running of M4M/PD-Ballet. To-date, examples of involvement include contributing to the overall study design; critically reviewing methods of assessment for relevance and clarity; and exploring and facilitating referral pathways for recruitment.

Developing theory of change

For peer review only - http://bmjopen.bmj.com/site/about/guidelines.xhtml

We will conduct stakeholder workshops to develop theory of change (ToC) and logic models. The ToC element will focus on summarising M4M/PD-Ballet at a strategic level, capturing possible pathways for the change process to achieve our outcomes. The logic models will focus on the programme specifics, assessing the change process at the level of implementing M4M/PD-Ballet. We will advance the traditional logic model by drawing from the recently developed Implementation Research Logic Model[57]. We will examine inputs (e.g. time), activities (e.g. recruitment), outputs (e.g. publications), outcomes (e.g. symptoms), implementation determinants (identified in the CFIR[51, 52]), implementation strategies, (drawing from the ERIC project[53, 54]) and mechanisms of action resulting from the implementation outcomes (informed by the implementation outcome taxonomy[58] and RE-AIM)[55, 56].

Recruitment

Recruitment for M4M will be through: 1) signposting via health and social care professionals, including midwives and health visitors; 2) healthcare referrals, including General Practitioners, clinical psychologists, psychiatrists and self-referral; 3) weighing clinics and other community and clinical centres for postnatal mothers, and; 4) social media groups and online forums aimed at new mothers.

Recruitment for PD-Ballet will be through: 1) the Movement Disorders Outpatient Clinic at King's College Hospital; 2) London South Parkinson's Excellence Network (PEN) using the London South PEN patient section of the website; 3) London South and National Clinical Research Network (CRN) websites and the EUROPAR website https://parkinsons-london.co.uk/europar/; 4) the Study Hub website (Parkinson's UK) and; 5) the ENB.

Stakeholders will be recruited through Breathe's (for M4M) and ENB's (for PD-Ballet) network of artists, staff and others involved in M4M/PD-Ballet.

Measures

Measures will be standardised across M4M/PD-Ballet, piloted and further refined (where applicable) through a co-design process with stakeholders to ensure their suitability.

For peer review only - http://bmjopen.bmj.com/site/about/guidelines.xhtml

Data on the acceptability[59] of M4M/PD-Ballet (primary implementation outcome measure) and training in the delivery of M4M/PD-Ballet (SHAPER-Implement's established implementation strategy), and data on the feasibility[59] and appropriateness[59] will be collected at 3 timepoints.

Implementation costs will be assessed using a previously developed costing proforma[60,61] on the time and financial resource associated with implementing M4M/PD-Ballet within existing care pathways. To explore commissioning costs, feedback from provider organisations will be sought (those responsible for providing the interventions) to establish a range of plausible unit prices that the NHS/partner organisations would be required to pay. To establish the impact of M4M/PD-Ballet on the wider utilisation of services (i.e. other health and service costs), service utilisation questionnaires on the frequency of contact with other NHS/non-NHS services over a 3-month retrospective period will be completed. Reported service use will be costed using published unit cost estimates for health care and other services. The EQ5D 3L[62] will be used to evaluate quality of life outcomes and quality-adjusted life year outcomes in the short-term.

To explore sustainment of M4M/PD-Ballet, the 'Normalisation Measure Development' questionnaire (NOMAD)[63] will be administered post-intervention.

Enrolment onto M4M/PD-Ballet will assess uptake and participation data and attrition rates will be used to assess adherence.

Sociodemographic data on participants and stakeholders will be collected (see Table 3 for further details on measures).

Qualitative measures

Semi-structured interviews partly informed by the study team's methods of assessment for previous hybrid trials[49,61] will complement the quantitative findings. Interviews will occur post-intervention and examine:

- Implementation strategies used to deliver M4M/PD-Ballet alongside strategies that may be important to consider by the local centres wishing to implement M4M/PD-Ballet after completion of SHAPER-Implement.
- Factors affecting the perceived acceptability, appropriateness, and feasibility of M4M/PD-Ballet.

For peer review only - http://bmjopen.bmj.com/site/about/guidelines.xhtml

- Fidelity of delivery – whether M4M/PD-Ballet is delivered in accordance with protocol (e.g., number and length of sessions, format and content covered).
- Fidelity of receipt - how the content of M4M/PD-Ballet is received by participants, including their experiences of engagement, comprehension of the content delivered, and application of acquired knowledge and skills to daily life.
- Factors affecting the sustained use of the knowledge and skills acquired from M4M/PD-Ballet for the long-term management of PND/PD (from participants) and the implementation of M4M/PD-Ballet into local services after the completion of SHAPER-Implement (from stakeholders).
- Unintended consequences (positive and negative) of M4M/PD-Ballet
- Stakeholders' willingness for continued involvement in M4M/PD-Ballet (i.e. intention to adopt) and participants' intentions to use the knowledge and skills gained from M4M/PD-Ballet and recommend M4M/PD-Ballet to others.

Data Analysis

Quantitative implementation outcomes

Parametric and nonparametric tests will be employed to compare survey responses between the two arms of the trial for M4M/PD-Ballet and the established implementation strategy (i.e. training arts leads/artists). Linear, logistic and Poisson regression models (depending on the distribution of the outcome) will explore the relationship between implementation (AIM, IAM and FIM[59]) and clinical trial primary (EPDS[64] for M4M and MDS-MNS[65] for PD-Ballet) and secondary outcome. Mediation analysis using Structural Equation Models under the causal framework will be employed to understand the potential pathways in which implementation outcomes and the implementation strategy of 'training', impact on the effectiveness of M4M/PD-Ballet[66,67]. All analyses will be conducted in STATA 16.0.

Psychometric assessment of the reliability, validity, and factorial structure of the implementation survey scales

The implementation outcomes scales are relatively new, hence require psychometric assessment. Cronbach's coefficient alpha will evaluate the reliability (internal consistency) of the IAM, FIM

For peer review only - http://bmjopen.bmj.com/site/about/guidelines.xhtml

and AIM scale-items (values range from 0-1 with internal consistency deemed acceptable when Cronbach's alpha[68] is ≥ 0.70[69]). A confirmatory factor analysis (CFA) model will be fitted, using the weighted least square estimator with a mean-and-variance-adjusted chi-squared method to handle ordered categorical items[70]. To evaluate overall model fit, the comparative fit index (CFI)[71], the Tucker Lewis index (TLI)[72] and the root mean square error of approximation (RMSEA)[73] will be calculated. A CFI and TFI value of > 0.90 indicates adequate fit to the data[74]. A value of RMSEA <0.05 indicates close fit, values between 0.05-0.08 suggest adequate model fit, and values >0.10 suggest poor model fit[74]. Psychometric analysis will be conducted using STATA 16 and Mplus 7.4[75].

Health economic outcomes

Using a combination of empirical and modelling methods, we will bring together evidence on clinical endpoints, quality-of life, implementation and intervention costs, and epidemiological data on prevalence and service utilisation to gauge the cost-effectiveness of delivering M4M/PD-Ballet at scale. We will build in evidence-based assumptions regarding the longer-term health and service resource impacts beyond the observable trial period. The cost-effectiveness of scalability under differing scenarios will be assessed with reference to existing thresholds, inclusive of those currently used by the National Institute for Health and Care Excellence (NICE) for assessing programme cost-effectiveness. Uncertainty in estimates will be explored through probabilistic and one-way sensitivity analysis. Cost-effectiveness will be evaluated from an NHS perspective, with a focus on exploring the incidence of incremental resource impacts associated with utilisation of NHS funded resources.

Qualitative implementation outcomes

A systematic classification process of coding themes/patterns in the data will be used with the main themes predetermined deductively by the Proctor et al[58] implementation outcomes framework and RE-AIM[76]. Sub-themes will be shaped inductively with the data until saturation is achieved. CFIR[77,78] will be used to analyse barriers/facilitators (anticipated and actual) to the implementation and sustainment of M4M/PD-Ballet. NVivo 12 will be used for data analysis.

For peer review only - http://bmjopen.bmj.com/site/about/guidelines.xhtml

Ethics and dissemination

M4M and PD-Ballet have been reviewed and approved by the West London and GTAC Research Ethics Committee (Reference: 20/PR/0813) and the HRA and Health and Care Research Wales Research Ethics Committee (Reference: 20/WA/0261). Informed consent will be sought from all research participants. The results will be reported in academic journals and conferences as well as other channels (e.g. workshops at centres involved, social media).

Conclusion

To the best of our knowledge, SHAPER-Implement is the largest study of its kind to directly address the APPGAHW recommendations[1] on examining how to translate arts interventions in a scalable way. In bringing two hybrid trials together, SHAPER-Implement will help bridge the gap between small-scale arts interventions and their large-scale implementation into clinical pathways in the NHS. We hope to achieve long-term commissioning and develop an evidence-based implementation blueprint to guide the implementation and scale-up of M4M/PD-Ballet after the trials' completion. As such, SHAPER-Implement could be transformative not just for the arts and academic organisations involved in this research, but also for the arts and health sectors more broadly.

For peer review only

List of abbreviations

AIM = Acceptability Intervention Measure

APPGAHW = All-Party Parliamentary Group on Arts, Health and Wellbeing

CFA = Confirmatory item Factor Analysis

CFI = Comparative Fit Index

CFIR = Consolidated Framework for Implementation Research

CRN = Clinical Research Network

PD-BALLET = Dance for Parkinson's

DSM-IV = Diagnostic and Statistical Manual of Mental Disorders, Fourth Edition

ENB = English National Ballet

EPDS = Edinburgh Postnatal Depression Scale

ERIC = Expert Recommendations for Implementing Change

FIM = Feasibility Intervention Measure

GP = General Practitioner

IAM = Intervention Appropriateness Measure

ImpRes = Implementation Science Research Development

M4M = Melodies for Mums

MoCA = Montreal Cognitive Assessment

MRC = Medical Research Council

NHS = National Health Service

NICE = National Institute for Health and Care Excellence

For peer review only - http://bmjopen.bmj.com/site/about/guidelines.xhtml

NMSS = Non-Motor Symptoms Scale

NoMAD = Implementation measure based on Normalization Process Theory

PD = Parkinson's Diseases

PEN = Parkinson's Excellence Network

PND = Postnatal Depression

QALYs = Quality-adjusted years of life lived

RE-AIM = Reach Effectiveness Adoption Implementation Maintenance

RMSEA = Root mean square error of approximation

SCID = Structured Clinical Interview for DSM-IV

SHAPER = Scale-up of Health-Arts Programmes Effectiveness-implementation Research

TLI = Tucker Lewis index

ToC = Theory of change

Declarations

Ethics approval and consent to participate

SHAPER-PND (the trial on the M4M program for individuals with PND) has been reviewed and given favourable opinion by the West London and GTAC Research Ethics Committee (REC Reference: 20/PR/0813). SHAPER-PD (the trial on the PD-BALLET program for individuals with PD) has been reviewed and given favourable opinion by the HRA and Health and Care Research Wales Research Ethics Committee (REC Reference: 20/WA/0261). Informed consent will be sought from all research participants for this study. The study will be conducted in accordance with the Good Clinical Practice and recommendations for physicians involved in research on human subjects adopted by the 18th World Medical Assembly, Helsinki 1964 and later revisions.

Consent for publication

Not applicable.

Availability of data and material

For peer review only - http://bmjopen.bmj.com/site/about/guidelines.xhtml

Not applicable, this manuscript does not contain any data.

Competing interests

NS is the Director of London Safety & Training Solution ltd, which offers training in patient safety, implementation solutions and human factors to healthcare organisations and the pharmaceutical industry. TS received funding from Cancer Alliances and Health Education England for training multidisciplinary teams in assessment and quality improvement methods in the United Kingdom. TS received consultancy fees from Roche Diagnostics in relation to implementation of innovations for multidisciplinary teams and their meetings in the United States of America. DF is a non-executive board director for Breathe Arts Health Research. But she receives no financial compensation for her involvement. CP has received research funding from Johnson & Johnson for research on depression and inflammation, and by a Wellcome Trust strategy award to the Neuroimmunology of Mood Disorders and Alzheimer's Disease (NIMA) Consortium (104025), which is also funded by Janssen, GlaxoSmithKline, Lundbeck and Pfizer. The work presented in this paper is unrelated to this funding. The remaining authors have no conflicts of interest to declare.

Funding

These trials included in SHAPER-Implement are part of the SHAPER programme, a Scaling-up Health-Arts Programme to scale up arts interventions. The programme is funded by the Wellcome Trust (award reference 219425/Z/19/Z).

IB, AH, and NS are funded by and RD is supported by the National Institute for Health Research (NIHR) via the 'Applied Research Collaboration South London' (NIHR ARC South London) at King's College Hospital National Health Service (NHS) Foundation Trust, London, UK. AH and NS are members of King's Improvement Science, which offers co-funding to the NIHR ARC South London and is funded by King's Health Partners (Guy's and St Thomas' NHS Foundation Trust, King's College Hospital NHS Foundation Trust, King's College London and South London and Maudsley NHS Foundation Trust), and Guy's and St Thomas' Foundation. IB (is further funded) and CP are supported by the NIHR Biomedical Research Centre at South London and Maudsley NHS Foundation Trust and CP is a NIHR Senior Investigator. MLB is further funded by Diabetes UK. RP is supported by HEartS, a project funded by the UK's Arts and Humanities Research Council to investigate the health, economic and social impact of the arts (Grant ref. AH/P005888/1).

For peer review only - http://bmjopen.bmj.com/site/about/guidelines.xhtml

The funding agreements ensured the authors’ independence in designing the study, interpreting the data, writing, and publishing the report. The views expressed are those of the authors and not necessarily reflect those of the NHS, the Wellcome Trust, the NIHR, the charities or the Department of Health and Social Care.

Authors' contributions

RD, TS, MBL, AH, CE, DF, PD, CP, HD, TO, RB, KS, LR, KH, AB, MM, RP, AP, RC, FDF, AH, AW, NC, IB, NS (i.e. all authors provided input on the design of the study and its conceptualisation) RD, TS, NS, AH, CE, IB, MLB were responsible for its drafting and edits. All authors provided feedback on all aspects of the content of the manuscript and its methodology. All authors read and approved the final version of the manuscript.

Acknowledgements We would like to thank the representatives of the people with Parkinson’s disease and postnatal depression, as well as the artists and health care professionals involved, for their time and commitment to the design of this study protocol.

For peer review only

References

1. All-Party Parliamentary Group on Arts. Health and wellbeing inquiry report. Creative health: the arts for health and wellbeing. All-Party Parliamentary Group on Arts; 2017.
2. Fancourt D, Finn S. What is the evidence on the role of the arts in improving health and well-being? A scoping review. WHO Regional Office for Europe; 2019.
3. Stuckey HL, Nobel J. The connection between art, healing, and public health: a review of current literature. *Am J Public Health.* 2010;100(2):254-63.
4. Boyce M, Bungay H, Munn-Giddings C, Wilson C. The impact of the arts in healthcare on patients and service users: A critical review. *Health Soc Care Community.* 2018;26(4):458-73.
5. Eccles MP, Mittman BS. Welcome to Implementation Science. *Implement Sci.* 2006;1(1).
6. Estevao C, Fancourt D, Dazzan P, Chaudhuri K, Sevdalis N, Woods A; et al. Scaling-up Health-Arts Programmes: the largest study in the world bringing mental health and arts into a national health service. *J B Psych Bulletin.* [in press].
7. Boath E, Henshaw C. The treatment of postnatal depression: A comprehensive literature review. *J Reprod Infant Psychol.* 2001;19(3):215-48.
8. Morrell CJ, Warner R, Slade P, Dixon S, Walters S, Paley G, et al. Psychological interventions for postnatal depression: cluster randomised trial and economic evaluation. The PoNDER trial. *Health Tech Asses* 2009;13(30):1-153.
9. National Institute for Health Care Excellence. Parkinson's disease in adults: National Institute for Health and Care Excellence; 2017.
10. UK Parkinson's. UK Parkinson's audit - Transforming care. UK Parkinson's; 2017.
11. Bauer A, Parsonage M, Knapp M, Iemmi V, Adelaja B. The costs of perinatal mental health problems. Centre for Mental Health and London School of Economics; 2014.
12. Parkinson's UK. The cost of Parkinson's: the financial impact of living with the condition. Parkinson's UK; 2017.
13. Soundy A, Stubbs B, Roskell C. The Experience of Parkinson's Disease: A Systematic Review and Meta-Ethnography. *Sci World J.* 2014;2014:613592.
14. Yang W, Hamilton JL, Kopil C, Beck JC, Tanner CM, Albin RL, et al. Current and projected future economic burden of Parkinson's disease in the U.S. *Parkinson's Dis.* 2020;6(1):15.
15. Bledsoe SE, Grote NK. Treating Depression During Pregnancy and the Postpartum: A Preliminary Meta-Analysis. *Res Soc Work Pract.* 2006;16(2):109-20.
16. Cooper PJ, Murray L, Wilson A, Romaniuk H. Controlled trial of the short- and long-term effect of psychological treatment of post-partum depression. I. Impact on maternal mood. *Br J Psychiatry.* 2003;182:412-9.
17. Milgrom J, Negri LM, Gemmill AW, McNeil M, Martin PR. A randomized controlled trial of psychological interventions for postnatal depression. *Br J Clin Psychol.* 2005;44(Pt 4):529-42.
18. Cooper PJ, Murray, L., & Halligan, S. L. Treatment of postpartum depression. Winnicott Research Unit, University of Reading, UK: Centre of Excellence for Early Childhood Development; 2010.

For peer review only - http://bmjopen.bmj.com/site/about/guidelines.xhtml

19. Booth FW, Roberts CK, Laye MJ. Lack of exercise is a major cause of chronic diseases. *Compr Physiol.* 2012;2(2):1143-211.
20. Chaudhuri KR, Titova N. Societal burden and persisting unmet needs of Parkinson's disease. *Eur Neurol Rev.* 2019;14(1):28-35.
21. Martinez-Martin P, Rodriguez-Blazquez C, Kurtis MM, Chaudhuri KR. The impact of non-motor symptoms on health-related quality of life of patients with Parkinson's disease. *Mov Disord.* 2011;26(3):399-406.
22. Bognar S, DeFaria AM, O'Dwyer C, Pankiw E, Simic Bogler J, Teixeira S, et al. More than just dancing: experiences of people with Parkinson's disease in a therapeutic dance program. *Disabill Rehabil.* 2017;39(11):1073-8.
23. Fancourt D, Perkins R. Associations between singing to babies and symptoms of postnatal depression, wellbeing, self-esteem and mother-infant bond. *Public Health.* 2017;145:149-52.
24. Fancourt D, Perkins R. Effect of singing interventions on symptoms of postnatal depression: three-arm randomised controlled trial. *Br J Psychiatry.* 2018;212(2):119-21.
25. Fancourt D, Perkins R. The effects of mother–infant singing on emotional closeness, affect, anxiety, and stress hormones. *Music Sci.* 2018;1.
26. Fancourt D, Perkins R. Creative interventions for symptoms of postnatal depression: A process evaluation of implementation. *Arts Health.* 2019;11(1):38-53.
27. Houston S, McGill A. A mixed-methods study into ballet for people living with Parkinson's. *Arts & health.* 2013;5(2):103-19.
28. Cultural Commissioning Programme Case Study. English National Ballet Dance for Parkinson's: Harnessing the power of dance to improve Health & Wellbeing. 2015.
29. Fancourt D, Perkins R. Does attending community music interventions lead to changes in wider musical behaviours? The effect of mother–infant singing classes on musical behaviours amongst mothers with symptoms of postnatal depression. *Psychol Music.* 2017;47(1):132-43.
30. Perkins R, Yorke S, Fancourt D. How group singing facilitates recovery from the symptoms of postnatal depression: a comparative qualitative study. *BMC Psychol.* 2018;6(1):41.
31. Estevao C, Bind R, Fancourt D, Burton A, Sawyer K, Hazelgrove K, et al. The SHAPER-PND trial: clinical effectiveness protocol of a community singing intervention for postnatal depression *BMJ Open* [in press].
32. PD protocol. [in press].
33. Curran GM, Bauer M, Mittman B, Pyne JM, Stetler C. Effectiveness-implementation hybrid designs: combining elements of clinical effectiveness and implementation research to enhance public health impact. *Med Care.* 2012;50(3):217-26.
34. King's Cultural Community. Arts, health & wellbeing: King's College London [Available from: https://www.kcl.ac.uk/cultural/arts-health-wellbeing].
35. King's Cultural Community. Arts-enhanced health education [Available from: https://www.kcl.ac.uk/cultural/arts-health-wellbeing/education].
36. Grigoriadis S, Wilton AS, Kurdyak PA, Rhodes AE, VonderPorten EH, Levitt A, et al. Perinatal suicide in Ontario, Canada: a 15-year population-based study. *Cmaj.* 2017;189(34):1085-92.
37. Slomian J, Honvo G, Emonts P, Reginster J-Y, Bruyère O. Consequences of maternal postpartum depression: A systematic review of maternal and infant outcomes. *Journal of Women's health* 2019;15.
38. Pearlstein T, Howard M, Salisbury A, Zlotnick C. Postpartum depression. *Am J Obstet Gynecol.* 2009;200(4):357-64.
39. Alison C, Scott S. Pharmacologic and Hormonal Treatments for Postpartum Depression. *Psychiatr Ann.* 2005;35(5):568-76.
40. Pearlstein T, Howard M, Salisbury M, Zlotnick C. Postpartum depression. *Am J Obstet Gynecol.* 2009;200(4):357-64.

For peer review only - http://bmjopen.bmj.com/site/about/guidelines.xhtml

41. Becki H. NHS England's Access and Waiting Times Programme Perinatal Mental Health. 2015. p. http://www.swscn.org.uk/wp/wp-content/uploads/2015/12/-07-31-presentation-NHSE-perinatal-programme.pdf.
42. Yang G, Schmiel L, Zhou M, Cintina I, Hogan P. Economic Burden and Future Impact of Parkinson's Disease
Final Report. The Lewin Group; 2019.
43. Parkinson's UK. The incidence and prevalence of Parkinson's in the UK. Parkinson's UK; 2018.
44. Kelberman MA, Vazey EM. New Pharmacological Approaches to Treating Non-Motor Symptoms of Parkinson's Disease. *Curr Pharmacol Rep.* 2016;2(6):253-61.
45. Zahoor I, Shafi A, Haq E. Pharmacological Treatment of Parkinson's Disease. Parkinson's Disease: Pathogenesis and Clinical Aspects. Brisbane Codon Publications; 2018.
46. Craig P, Dieppe P, Macintyre S, Michie S, Nazareth I, Petticrew M. Developing and evaluating complex interventions: the new Medical Research Council guidance. *BMJ.* 2008;337:a1655.
47. Craig P, Dieppe P, Macintyre S, Michie S, Nazareth I, Petticrew M. Developing and evaluating complex interventions: the new Medical Research Council guidance. *Int J Nurs Stud.* 2013;50(5):587-92.
48. Hull L, Goulding L, Khadjesari Z, Davis R, Healey A, Bakolis I, et al. Designing high-quality implementation research: development, application, feasibility and preliminary evaluation of the implementation science research development (ImpRes) tool and guide. *Implement Sci.* 2019;14(1):80.
49. Soukup T, Hull L, Smith EL, Healey A, Bakolis I, Amiel SA, et al. Effectiveness-implementation hybrid type 2 trial evaluating two psychoeducational programmes for severe hypoglycaemia in type 1 diabetes: implementation study protocol. *BMJ Open.* 2019;9(11):303-70.
50. Michie S, van Stralen MM, West R. The behaviour change wheel: a new method for characterising and designing behaviour change interventions. *Implement Sci.* 2011;6:42-.
51. Consolidated Framework for Implementation Research. 2021 [Available from: https://cfirguide.org/].
52. Damschroder LJ, Aron DC, Keith RE, Kirsh SR, Alexander JA, Lowery JC. Fostering implementation of health services research findings into practice: a consolidated framework for advancing implementation science. *Implement Sci.* 2009;4(1):50.
53. Powell BJ, Waltz TJ, Chinman MJ, Damschroder LJ, Smith JL, Matthieu MM, et al. A refined compilation of implementation strategies: results from the Expert Recommendations for Implementing Change (ERIC) project. *Implement Sci.* 2015;10(1):21.
54. Waltz TJ, Powell BJ, Matthieu MM, Damschroder LJ, Chinman MJ, Smith JL, et al. Use of concept mapping to characterize relationships among implementation strategies and assess their feasibility and importance: results from the Expert Recommendations for Implementing Change (ERIC) study. *Implement Sci.* 2015;10(1):109.
55. RE-AIM 2021 [Available from: https://www.re-aim.org/].
56. Glasgow RE, Vogt TM, Boles SM. Evaluating the public health impact of health promotion interventions: the RE-AIM framework. *Am J Public Health.* 1999;89(9):1322-7.
57. Smith JD, Li DH, Rafferty MR. The Implementation Research Logic Model: a method for planning, executing, reporting, and synthesizing implementation projects. *Implement Sci.* 2020;15(1):84.
58. Proctor E, Silmere H, Raghavan R, Hovmand P, Aarons G, Bunger A, et al. Outcomes for implementation research: conceptual distinctions, measurement challenges, and research agenda. *Adm Policy Ment Health.* 2011;38(2):65-76.

For peer review only - http://bmjopen.bmj.com/site/about/guidelines.xhtml

59. Weiner BJ, Lewis CC, Stanick C, Powell BJ, Dorsey CN, Clary AS, et al. Psychometric assessment of three newly developed implementation outcome measures. *Implement Sci.* 2017;12(1):108.
60. Soukup T HL, Smith EL, Rogers H, Healey A, Ioannis B, Amiel S, Sevdalis N, PWD Group. The role, value and challenges of stakeholder involvement in implementation research. *BMJ Q&S.* [in press].
61. Soukup T, Hull L, Healey A, Bakolis I, Smith E, Amiel S, et al. Interview topic guides exploring implementation outcomes across stakeholder groups (Version 2) Zenodo; 2020 [Available from: http://doi.org/10.5281/zenodo.3865707].
62. Herdman M, Gudex C, Lloyd A, Janssen M, Kind P, Parkin D, et al. Development and preliminary testing of the new five-level version of EQ-5D (EQ-5D-5L). *Qual Life Res.* 2011;20(10):1727-36.
63. Finch TG, M; May, CR; Mair, FS; Murray, E; Treweek, S; Steen, IN; McColl, EM; Dickinson, C; Rapley, T. NoMad: Implementation measure based on Normalization Process Theory. 2015 [Available from: http://www.normalizationprocess.org].
64. Cox JL, Holden JM, Sagovsky R. Detection of Postnatal Depression: Development of the 10-item Edinburgh Postnatal Depression Scale. *Br J Psychiatry.* 1987;150(6):782-6.
65. Martinez-Martin P, Chaudhuri KR, Rojo-Abuin JM, Rodriguez-Blazquez C, Alvarez-Sanchez M, Arakaki T, et al. Assessing the non-motor symptoms of Parkinson's disease: MDS-UPDRS and NMS Scale. *Eur J Neurol.* 2015;22(1):37-43.
66. De Stavola BL, Daniel RM, Ploubidis GB, Micali N. Mediation Analysis With Intermediate Confounding: Structural Equation Modeling Viewed Through the Causal Inference Lens. *American Journal of Epidemiology.* 2015;181(1):64-80.
67. R. K. Principles and practice of structural equation modeling. 3rd ed. New York: Guilford press; 2011.
68. Cronbach LJ. Coefficient alpha and the internal structure of tests. *Psychometrika.* 1951;16(3):297-334.
69. DeVellis R. Scale development: Theory and applications: Sage publications; 2016.
70. Muthén B, du Toit S, Spisic D. Robust inference using weighted least squares and quadratic estimating equations in latent variable modeling with categorical and continuous outcomes. *Psychometrika.* 1997;75(1):40-5.
71. Bentler PM. Comparative fit indexes in structural models. *Psychol Bull.* 1990;107(2):238-46.
72. Tucker LR, Lewis C. A reliability coefficient for maximum likelihood factor analysis. *Psychometrika.* 1973;38(1):1-10.
73. Steiger JH. Statistically-Based Tests for the Number of Common Factors. 1980.
74. Hu Lt, Bentler PM. Cutoff criteria for fit indexes in covariance structure analysis: Conventional criteria versus new alternatives. *Struct Equ Modeling.* 1999;6(1):1-55.
75. Muthén LK, Muthén BO. Mplus User's Guide. 5th ed. Los Angeles, CA: Muthén & Muthén; 1998-2007.
76. Harden S, Smith M, Ory M, Smith-Ray R, Estabrooks P, Glasgow R. Re-aim in clinical, community, and corporate settings: perspectives, strategies, and recommendations to enhance public health impact. *Front Public Health.* 2018.
77. Damschroder LJ, Hagedorn HJ. A guiding framework and approach for implementation research in substance use disorders treatment. *Psychol Addict Behav.* 2011;25(2):194-205.
78. Kirk MA, Kelley C, Yankey N, Birken SA, Abadie B, Damschroder L. A systematic review of the use of the Consolidated Framework for Implementation Research. *Implement Sci.* 2016;11(1):72.

For peer review only - http://bmjopen.bmj.com/site/about/guidelines.xhtml

For peer review only

For peer review only - http://bmjopen.bmj.com/site/about/guidelines.xhtml

Table 1. Overview of the Curran et al[34] criteria for hybrid trial type 2 design and how they apply to M4M and PD-Ballet hybrid evaluations

Study Characteristics for Hybrid Trial Type 2, as per Curran et al[34] criteria		Trialled Arts Therapies	
		**Melodies for Mums**	**PD-Ballet**
Research aims	**Determine feasibility and potential utility of an implementation intervention/strategy**<br><br>*Determine effectiveness of a clinical intervention	**Determine feasibility and potential utility of M4M to facilitate future implementation and scale up**<br><br>Determine clinical effectiveness of M4M	**Determine feasibility and potential utility of PD-Ballet to facilitate future implementation and scale up**<br><br>Determine clinical effectiveness of PD-Ballet
Research questions	**Does the implementation method show promise (either alone or in comparison with another method) in facilitating implementation of a clinical treatment?**<br><br>*Will a clinical treatment work in these settings/ for these patients?	**Will the implementation method show promise (alone) in facilitating M4M in people with PND?**<br><br>What is the clinical impact of M4M?	**Will the implementation method show promise (alone) in facilitating PD-Ballet in people with PD?**<br><br>What is the clinical impact of PD-Ballet?

For peer review only - http://bmjopen.bmj.com/site/about/guidelines.xhtml

Unit of randomization	**Provider, clinical unit, facility or system, as per type although may be nonrandomized, e.g., case study**<br><br>Patient or clinical unit, as per type 1	**Providers, as per type 3 (non-randomised)**<br><br>Patients, as per type 1 (randomised)	**Providers, as per type 3 (non-randomised)**<br><br>Patients, as per type 1 (randomised)
Comparison conditions	**Provider, clinical unit, facility, system: implementation as usual, or competing implementation strategy although may be nonrandomized, e.g., case study**<br>Placebo, treatment as usual, or competing treatment, as per type 1	**Facility, as per hybrid type 3**<br><br>Treatment as usual, as per hybrid type 1	**Facility, as per hybrid type 3**<br><br>Treatment as usual, as per hybrid type 1
Sampling frames	**Providers/clinics/facility/systems: consider "optimal" cases**<br><br>Patient: limited restrictions, but some inclusion/exclusion criteria	**Providers/facility: artists, psychiatrists/neurologists, referrers, commissioners, support staff; community centres**<br><br>Patient: inclusion/exclusion criteria used	**Providers/facility: artists, psychiatrists/neurologists, referrers, commissioners, support staff; dance centres**<br><br>Patient: inclusion/exclusion criteria used

For peer review only - http://bmjopen.bmj.com/site/about/guidelines.xhtml

Evaluation methods	**Mixed method; quantitative, qualitative; formative and summative**<br><br>Quantitative, summative	**Quantitative surveys (§AIM,[61] ǀIAM[61], ¶FIM,[61] **NoMaD,[65] costing proforma[62, 63]) and interviews[50, 63]**<br><br>Quantitative surveys (†EPDS[66]; EQ-5D 3L; [64])	**Quantitative surveys (§AIM,[61] ǀIAM[61], ¶FIM,[61] **NoMaD,[65] costing proforma[62, 63]) and interviews[50, 63]**<br><br>Quantitative surveys (†EPDS[66]; EQ-5D 3L; [64])
Measures	**Adoption of clinical treatment and fidelity to it, as well as related factors**<br><br>Patient symptoms and functioning, possibly cost effectiveness	**Adoption of M4M, fidelity of its delivery and receipt, acceptability, appropriateness, feasibility, sustainability, reach, unintended consequences, contextual factors, implementation strategies, implementation costs[61-64]**<br><br>Patient symptoms and functioning, cost effectiveness	**Adoption of PD-Ballet, fidelity of its delivery and receipt, acceptability, appropriateness, feasibility, sustainability, reach, unintended consequences, contextual factors, implementation strategies, implementation costs[61-64]**<br><br>Patient symptoms and functioning, cost effectiveness

Note. *Curran et al: "one of these aims/research questions might take precedence, for example in a case where the test of an implementation intervention/strategy is exploratory".[34] †EPDS=Edinburgh Postnatal Depression Scale. ‡NMSS=Non-Motor Symptoms Scale. §AIM=Acceptability of Intervention Measure. ǀIAM=Intervention Appropriateness Measure. ¶FIM=Feasibility of Intervention Measure. **NoMaD=Implementation measure based on Normalization Process Theory.

Bold type = information relating to the implementation effectiveness evaluation. Normal type = information relating to the clinical effectiveness evaluation, discussed in more detail in a separate protocols[32,33]

For peer review only - http://bmjopen.bmj.com/site/about/guidelines.xhtml

Table 2. Overview of the structure and delivery of Melodies for Mums and PD-Ballet interventions

Programme structure and delivery	Melodies for Mums	PD-Ballet
Arts partner	Breathe Arts Health Research	English National Ballet
Targeted health condition	Postnatal depression	Parkinson's disease
Setting	Community children's centres OR online (depending on COVID-19 guidelines)	Community dance studios OR online (depending on COVID-19 guidelines)
Delivery structure	A weekly 1-hour group session over 10 weeks	A weekly 2-hour 15-minute 12 group session over 12 weeks
N of intervention cycles	10 cycles, with a morning and afternoon cycle held across 5 separate10-weekly periods	3 cycles (stratified by motor advancement – mild/moderate/severe)
N of participants per cohort	12-15	30-35
Total *N* of participants in the intervention	270 mums (plus their respective babies)	107 individuals with Parkinson's*
Follow-ups (as related to the delivery of sessions)	No follow-up	No follow-up
Artists per session	2 (1 artist, 1 assistant)	4 (2 associate dance artists / 2 associate musicians), plus 1 *artist-in-training*
Comparator intervention (sample size)	†Usual treatment (*N*=130)	Usual treatment (*N*=53)

Note. N=number. *The three cohorts differ in the severity of Parkinson's symptoms: mild, moderate, and severe ** The 2 days training is required for each stage of motor advancement so the trainer would need to attend three 2-day training sessions if they running programs for all 3 groups of motor advancement (mild/moderate/severe).

Table 3. Data collection plan for the implementation effectiveness evaluation of Melodies for Mums and PD-Ballet interventions

For peer review only - http://bmjopen.bmj.com/site/about/guidelines.xhtml

**Outcome**	**Definition**	**Form of measurement**	**Timepoint(s)**	**Stakeholder group**
**Implementation effectiveness**				
Acceptability	Extent to which M4M/PD-Ballet are perceived to be agreeable and acceptable for management of PND and PD, respectively.<br><br>Extent to which training in the delivery of M4M/PD-Ballet (i.e., SHAPER-Implement's established implementation strategy) is considered acceptable to arts leads/artists	AIM survey<br><br>Interview	During intervention, post intervention and 3-6 months post-intervention follow-up	Participants, and wider stakeholder groups
Appropriateness	Extent to which M4M/PD-Ballet are perceived to be fit and relevant for management of PND and PD, respectively.	IAM survey<br><br>Interview	During intervention, post intervention and 3-6 months post-intervention follow-up	Participants, and wider stakeholder groups
Feasibility	Extent to which M4M/PD-Ballet can be successfully used or carried out to reduce incidents of PND and PD.	FIM survey<br><br>Interview	Baseline, post intervention and 3-6 months post-intervention follow-up	Participants, and wider stakeholder groups
Fidelity of delivery	Extent to which M4M/PD-Ballet sessions were delivered according to protocol.	Interview	Post intervention	Deliverers
Fidelity of receipt	Extent to which M4M/PD-Ballet are received as intended.	Interview	Post intervention	Participants
Adoption	Intention to adopt and use the knowledge and skills learned in M4M/PD-Ballet in everyday management of PND and PD.	Interview	Post intervention	Participants, and wider stakeholder groups

For peer review only - http://bmjopen.bmj.com/site/about/guidelines.xhtml

Sustainability	Facilitators and barriers to sustained use of M4M/PD-Ballet.	Interview and the NoMad survey	Post-intervention	Participants, and wider stakeholder groups
Uptake	The proportion and representativeness of individuals willing to participate in M4M/PD-Ballet.	Trial records	Baseline	Provided by trial manager
Adherence	The number of individuals attending each M4M/PD-Ballet session and the number of dropouts	Trial records	Throughout intervention period	Provided by trial manager
Unintended consequences	Positive or negative consequences that are not anticipated at the time of implementation of M4M/PD-Ballet.	Interview	Post intervention	Participants, and wider stakeholder groups
Implementation strategies*	Strategies used to deliver and implement M4M/PD-Ballet.	Interview	Post intervention	Wider stakeholder groups
**Implementation costs and cost effectiveness**				
Implementation costs	Costs associated with prospective implementation of M4M/PD-Ballet.	Costing proforma	Post intervention	Participants, and wider stakeholder groups
Cost-benefit analysis	Costs associated with M4M/PD-Ballet versus the benefits in terms of the outcomes.	EQ-5D 3L	Post intervention	Participants
**Clinical effectiveness****				
	The impact of M4M/PD-Ballet on the clinical outcomes of interest.	EPDS survey<br><br>NMSS survey	Baseline, post intervention and 3-6 months post-intervention follow-up	Participants

Note. PD=Parkinson's Disease. PND=Post-natal Depression. AIM=Acceptability of Intervention Measure. IAM=Intervention Appropriateness Measure. FIM=Feasibility of Intervention Measure. EPDS=Edinburgh Postnatal Depression Scale (Melodies for Mums). NMSS=Non-Motor Symptoms Scale (Dance for Parkinson's). NoMAD=Implementation measure based on Normalization Process Theory.

For peer review only - http://bmjopen.bmj.com/site/about/guidelines.xhtml

- As part of SHAPER-Implement we will be assessing the acceptability and benefit of 'training arts leads/artists' in the delivery of M4M/PD-ballet (i.e. our established implementation strategy) but we will also be examining other implementation strategies that may be of use in the wider-scale implementation of M4M/PD-Ballet.

** For further information on the clinical effectiveness evaluations, please refer to the corresponding clinical protocols for M4M[32] and PD-Ballet[33].

For peer review only - http://bmjopen.bmj.com/site/about/guidelines.xhtml

SPIRIT
STANDARD PROTOCOL ITEMS: RECOMMENDATIONS FOR INTERVENTIONAL TRIALS

SPIRIT 2013 Checklist: Recommended items to address in a clinical trial protocol and related documents*

Section/item	Item No	Description	Addressed on page number
**Administrative information**			
Title	1	Descriptive title identifying the study design, population, interventions, and, if applicable, trial acronym	__1___________
Trial registration	2a	Trial identifier and registry name. If not yet registered, name of intended registry	__2/3_________
	2b	All items from the World Health Organization Trial Registration Data Set	__NA______
Protocol version	3	Date and version identifier	__NA________
Funding	4	Sources and types of financial, material, and other support	_____19______
Roles and responsibilities	5a	Names, affiliations, and roles of protocol contributors	___1&20_______
	5b	Name and contact information for the trial sponsor	___3 & 18______
	5c	Role of study sponsor and funders, if any, in study design; collection, management, analysis, and interpretation of data; writing of the report; and the decision to submit the report for publication, including whether they will have ultimate authority over any of these activities	_________NA____
	5d	Composition, roles, and responsibilities of the coordinating centre, steering committee, endpoint adjudication committee, data management team, and other individuals or groups overseeing the trial, if applicable (see Item 21a for data monitoring committee)	_____NA_______

For peer review only

Introduction

Background and rationale	6a	Description of research question and justification for undertaking the trial, including summary of relevant studies (published and unpublished) examining benefits and harms for each intervention	__4-5_______
	6b	Explanation for choice of comparators	__5, 7-9________
Objectives	7	Specific objectives or hypotheses	____4-5______
Trial design	8	Description of trial design including type of trial (eg, parallel group, crossover, factorial, single group), allocation ratio, and framework (eg, superiority, equivalence, noninferiority, exploratory)	____7-9_______

Methods: Participants, interventions, and outcomes

Study setting	9	Description of study settings (eg, community clinic, academic hospital) and list of countries where data will be collected. Reference to where list of study sites can be obtained	_____6_______
Eligibility criteria	10	Inclusion and exclusion criteria for participants. If applicable, eligibility criteria for study centres and individuals who will perform the interventions (eg, surgeons, psychotherapists)	_______10-11__
9Interventions	11a	Interventions for each group with sufficient detail to allow replication, including how and when they will be administered	_______7-9____
	11b	Criteria for discontinuing or modifying allocated interventions for a given trial participant (eg, drug dose change in response to harms, participant request, or improving/worsening disease)	_______9_____
	11c	Strategies to improve adherence to intervention protocols, and any procedures for monitoring adherence (eg, drug tablet return, laboratory tests)	_______11-12__
	11d	Relevant concomitant care and interventions that are permitted or prohibited during the trial	___NA_______
Outcomes	12	Primary, secondary, and other outcomes, including the specific measurement variable (eg, systolic blood pressure), analysis metric (eg, change from baseline, final value, time to event), method of aggregation (eg, median, proportion), and time point for each outcome. Explanation of the clinical relevance of chosen efficacy and harm outcomes is strongly recommended	12-14, 25-26, 28-30
Participant timeline	13	Time schedule of enrolment, interventions (including any run-ins and washouts), assessments, and visits for participants. A schematic diagram is highly recommended (see Figure)	_____12______

For peer review only - http://bmjopen.bmj.com/site/about/guidelines.xhtml

Sample size	14	Estimated number of participants needed to achieve study objectives and how it was determined, including clinical and statistical assumptions supporting any sample size calculations	______10-11____
Recruitment	15	Strategies for achieving adequate participant enrolment to reach target sample size	_______12_____

Methods: Assignment of interventions (for controlled trials)

Allocation:

Sequence generation	16a	Method of generating the allocation sequence (eg, computer-generated random numbers), and list of any factors for stratification. To reduce predictability of a random sequence, details of any planned restriction (eg, blocking) should be provided in a separate document that is unavailable to those who enrol participants or assign interventions	____5-6______
Allocation concealment mechanism	16b	Mechanism of implementing the allocation sequence (eg, central telephone; sequentially numbered, opaque, sealed envelopes), describing any steps to conceal the sequence until interventions are assigned	__5-6_______
Implementation	16c	Who will generate the allocation sequence, who will enrol participants, and who will assign participants to interventions	___5-6____
Blinding (masking)	17a	Who will be blinded after assignment to interventions (eg, trial participants, care providers, outcome assessors, data analysts), and how	_____NA_____
	17b	If blinded, circumstances under which unblinding is permissible, and procedure for revealing a participant's allocated intervention during the trial	______NA_____

Methods: Data collection, management, and analysis

Data collection methods	18a	Plans for assessment and collection of outcome, baseline, and other trial data, including any related processes to promote data quality (eg, duplicate measurements, training of assessors) and a description of study instruments (eg, questionnaires, laboratory tests) along with their reliability and validity, if known. Reference to where data collection forms can be found, if not in the protocol	12-15, 25-26, 28-3
	18b	Plans to promote participant retention and complete follow-up, including list of any outcome data to be collected for participants who discontinue or deviate from intervention protocols	12-15, 25-26, 28-30

Data management	19	Plans for data entry, coding, security, and storage, including any related processes to promote data quality (eg, double data entry; range checks for data values). Reference to where details of data management procedures can be found, if not in the protocol	___14-15______
Statistical methods	20a	Statistical methods for analysing primary and secondary outcomes. Reference to where other details of the statistical analysis plan can be found, if not in the protocol	___14-15______
	20b	Methods for any additional analyses (eg, subgroup and adjusted analyses)	___14-15_______
	20c	Definition of analysis population relating to protocol non-adherence (eg, as randomised analysis), and any statistical methods to handle missing data (eg, multiple imputation)	__14-15________
**Methods: Monitoring**			
Data monitoring	21a	Composition of data monitoring committee (DMC); summary of its role and reporting structure; statement of whether it is independent from the sponsor and competing interests; and reference to where further details about its charter can be found, if not in the protocol. Alternatively, an explanation of why a DMC is not needed	_______NA______
	21b	Description of any interim analyses and stopping guidelines, including who will have access to these interim results and make the final decision to terminate the trial	______NA______
Harms	22	Plans for collecting, assessing, reporting, and managing solicited and spontaneously reported adverse events and other unintended effects of trial interventions or trial conduct	_______NA_____
Auditing	23	Frequency and procedures for auditing trial conduct, if any, and whether the process will be independent from investigators and the sponsor	______NA______
**Ethics and dissemination**			
Research ethics approval	24	Plans for seeking research ethics committee/institutional review board (REC/IRB) approval	___16, 18______
Protocol amendments	25	Plans for communicating important protocol modifications (eg, changes to eligibility criteria, outcomes, analyses) to relevant parties (eg, investigators, REC/IRBs, trial participants, trial registries, journals, regulators)	______18______

For peer review only - http://bmjopen.bmj.com/site/about/guidelines.xhtml

Consent or assent	26a	Who will obtain informed consent or assent from potential trial participants or authorised surrogates, and how (see Item 32)	______12, 16______
	26b	Additional consent provisions for collection and use of participant data and biological specimens in ancillary studies, if applicable	_______NA____
Confidentiality	27	How personal information about potential and enrolled participants will be collected, shared, and maintained in order to protect confidentiality before, during, and after the trial	____12, 16______
Declaration of interests	28	Financial and other competing interests for principal investigators for the overall trial and each study site	________18____
Access to data	29	Statement of who will have access to the final trial dataset, and disclosure of contractual agreements that limit such access for investigators	______18_____
Ancillary and post-trial care	30	Provisions, if any, for ancillary and post-trial care, and for compensation to those who suffer harm from trial participation	____NA_______
Dissemination policy	31a	Plans for investigators and sponsor to communicate trial results to participants, healthcare professionals, the public, and other relevant groups (eg, via publication, reporting in results databases, or other data sharing arrangements), including any publication restrictions	_____16_______
	31b	Authorship eligibility guidelines and any intended use of professional writers	_______20______
	31c	Plans, if any, for granting public access to the full protocol, participant-level dataset, and statistical code	_____NA______
**Appendices**			
Informed consent materials	32	Model consent form and other related documentation given to participants and authorised surrogates	________NA____
Biological specimens	33	Plans for collection, laboratory evaluation, and storage of biological specimens for genetic or molecular analysis in the current trial and for future use in ancillary studies, if applicable	_______NA_____

*It is strongly recommended that this checklist be read in conjunction with the SPIRIT 2013 Explanation & Elaboration for important clarification on the items. Amendments to the protocol should be tracked and dated. The SPIRIT checklist is copyrighted by the SPIRIT Group under the Creative Commons "Attribution-NonCommercial-NoDerivs 3.0 Unported" license.

For peer review only - http://bmjopen.bmj.com/site/about/guidelines.xhtml

BMJ Open

Study protocol: Randomised controlled hybrid type 2 trial evaluating the scale-up of two arts interventions for Postnatal depression and Parkinson's disease

Journal:	*BMJ Open*
Manuscript ID	bmjopen-2021-055691.R1
Article Type:	Protocol
Date Submitted by the Author:	29-Sep-2021
Complete List of Authors:	Soukup, Tayana ; King's College London, Centre Implementation Science; King's College London<br>Davis, Rachel; Institute of Psychiatry Psychology and Neuroscience Division of Psychology and Systems Sciences<br>Baldellou Lopez, Maria<br>Healey, Andy; King's College London, IOPPN<br>Estevao, Carolina; King's College London, nstitute of Psychiatry, Psychology & Neuroscience<br>Fancourt, Daisy; University College London, Department of Behavioural Science and Health<br>Dazzan, Paola; King's College London<br>Pariante, Carmine; King's College London, Psychological Medicine<br>Dye, Hannah; The Clarence Centre<br>Osborn, Tim; The Clarence Centre<br>Bind, Rebecca; King's College London, Department of Psychological Medicine<br>Sawyer, Kristi; King's College London, Department of Psychological Medicine<br>Rebecchini, Lavinia; King's College London, Department of Psychological Medicine<br>Hazelgrove, Katie; King's College London, Department of Psychological Medicine<br>Burton, Alexandra; University College London, Division of Psychiatry<br>Manoharan, Manonmani; South London and Maudsley NHS Foundation Trust, lewisham Perinatal team<br>Perkins, Rosie; Royal College of Music, Centre for Performance Science; Imperial College London, Faculty of Medicine<br>Podlewska, Aleksandra<br>Chaudhuri, Ray; King\'s College London<br>Derbyshire-Fox, Fleur<br>Hartley, Alison<br>Woods, Anthony; King's College London, Department of Psychological Medicine<br>Crane, Nikki; King's College London<br>Bakolis, Ioannis; King's College London,<br>Sevdalis, Nick; King's College London,
Primary Subject	Evidence based practice

Heading:	
Secondary Subject Heading:	Health economics, Health policy, Health services research
Keywords:	Maternal medicine < OBSTETRICS, Parkinson-s disease < NEUROLOGY, HEALTH ECONOMICS, HEALTH SERVICES ADMINISTRATION & MANAGEMENT

SCHOLARONE™
Manuscripts

I, the Submitting Author has the right to grant and does grant on behalf of all authors of the Work (as defined in the below author licence), an exclusive licence and/or a non-exclusive licence for contributions from authors who are: i) UK Crown employees; ii) where BMJ has agreed a CC-BY licence shall apply, and/or iii) in accordance with the terms applicable for US Federal Government officers or employees acting as part of their official duties; on a worldwide, perpetual, irrevocable, royalty-free basis to BMJ Publishing Group Ltd ("BMJ") its licensees and where the relevant Journal is co-owned by BMJ to the co-owners of the Journal, to publish the Work in this journal and any other BMJ products and to exploit all rights, as set out in our licence.

The Submitting Author accepts and understands that any supply made under these terms is made by BMJ to the Submitting Author unless you are acting as an employee on behalf of your employer or a postgraduate student of an affiliated institution which is paying any applicable article publishing charge ("APC") for Open Access articles. Where the Submitting Author wishes to make the Work available on an Open Access basis (and intends to pay the relevant APC), the terms of reuse of such Open Access shall be governed by a Creative Commons licence – details of these licences and which Creative Commons licence will apply to this Work are set out in our licence referred to above.

Other than as permitted in any relevant BMJ Author's Self Archiving Policies, I confirm this Work has not been accepted for publication elsewhere, is not being considered for publication elsewhere and does not duplicate material already published. I confirm all authors consent to publication of this Work and authorise the granting of this licence.

For peer review only

Study protocol: Randomised controlled hybrid type 2 trial evaluating the scale-up of two arts interventions for Postnatal depression and Parkinson's disease

Tayana Soukup,[1]⌧* Rachel Davis,[1]⌧* Maria Baldellou Lopez,[1] Andy Healey,[1] Carolina Estevao[3], Daisy Fancourt[5], Paola Dazzan[3], Carmine Pariante[3], Hannah Dye[4], Tim Osborn[4], Rebecca Bind[3], Kristi Sawyer[3], Lavinia Rebecchini[3], Katie Hazelgrove[3], Alexandra Burton[5], Manonmani Manoharan[6], Rosie Perkins[7,8], Aleksandra Podlewska[9], Ray Chaudhuri[9], Fleur Derbyshire-Fox[10], Alison Hartley[10], Anthony Woods[3], Nikki Crane[3], Ioannis Bakolis,[1,2]** Nick Sevdalis[1]**.

*These two authors have contributed equally and share first authorship.

** These two authors have contributed equally and share senior authorship.

Author affiliations

[1] Centre for Implementation Science, King's College London

[2] Department of Biostatistics and Health Informatics, King's College London

[3] Department of Psychological Medicine, King's College London

[4] Breathe Arts Health Research, London.

[5] Department of Behavioural Science and Health, University College London.

[6] South London and Maudsley NHS Foundation Trust, London.

[7] Centre for Performance Science, Royal College of Music, London.

[8] Faculty of Medicine, Imperial College London

[9] Department of Basic and Clinical Neuroscience, King's College London

[10] English National Ballet, London

Full address for correspondence

⌧Dr Rachel Davis, Centre for Implementation Science, Health Service & Population Research Department, Institute of Psychiatry, Psychology and Neuroscience King's College London, 16 De Crespigny Park, London SE5 8AF. Email: rachel.davis@kcl.ac.uk

For peer review only

Abstract

Introduction Research on the benefits of 'arts' interventions to improve individuals' physical, social and psychological wellbeing is growing, but evidence on implementation and scale-up into health and social care systems is lacking. This protocol reports the SHAPER-Implement programme (Scale-up of Health-Arts Programmes Effectiveness-Implementation Research), aimed at studying the impact, implementation and scale-up of: Melodies for Mums (M4M), a singing intervention for postnatal depression; and PD-Ballet a dance intervention for Parkinson's disease. We examine how they could be embedded in clinical pathways to ensure their longer-term sustainability.

Methods and analysis A randomized two-arm effectiveness-implementation hybrid type 2 trial design will be used across M4M/PD-Ballet. We will assess the implementation in both study arms (intervention versus control), and the cost-effectiveness of implementation. The design and measures, informed by literature and previous research by the study team, were refined through stakeholder engagement. Participants (400 in M4M; 160 in PD-Ballet) will be recruited to the intervention or control group (2:1 ratio). Further implementation data will be collected from stakeholders involved in referring to, delivering, or supporting M4M/PD-Ballet (N = 25-30 for each intervention).

A mixed-methods approach (surveys and semi-structured interviews) will be employed. 'Acceptability' (measured by the 'Acceptability Intervention Measure') is the primary implementation endpoint for M4M/PD-Ballet. Relationships between clinical and implementation outcomes, implementation strategies (e.g. training) and outcomes will be explored using generalised linear mixed models. Qualitative data will assess factors affecting the acceptability, feasibility, and appropriateness of M4M/PD-Ballet, implementation strategies and longer-term sustainability. Costs associated with implementation and future scale-up will be estimated.

Ethics and dissemination SHAPER-PND (the M4M trial) and SHAPER-PD (the PD trial) are approved by the West London and GTAC (20/PR/0813) and the HRA and Health and Care Research Wales (REC Reference: 20/WA/0261) Research Ethics Committees. Study findings will be disseminated through scientific peer-reviewed journals and scientific conferences.

Trial registration numbers: Both trials are registered with NIH US National Library of Medicine, ClinicalTrials.gov. The trial registration numbers, URLs of registry records, and dates of registration are:

For peer review only

For peer review only - http://bmjopen.bmj.com/site/about/guidelines.xhtml

- PD-Ballet: URLNCT04719468 https://eur03.safelinks.protection.outlook.com/?url=https%3A%2F%2Fwww.clinicaltrials.gov%2Fct2%2Fshow%2FNCT04719468%3Fterm%3DNCT04719468%26draw%3D2%26rank%3D1&data=04%7C01%7Crachel.davis%40kcl.ac.uk%7C11a7c5142782437919f808d903111449%7C8370cf1416f34c16b83c724071654356%7C0%7C0%7C637544194261659488%7CUnknown%7CTWFpbGZsb3d8eyJWIjoiMC4wLjAwMDAiLCJQIjoiV2luMzIiLCJBTiI6Ik1haWwiLCJXVCI6Mn0%3D%7C1000&sdata=jNjPRC3e9oY6oGCeNQ0zE%2FusMlZqUd2r4bFLTvHRDO0%3D&reserved=0 (date of registration – January 22st 2021)

- Melodies for Mums: NCT04834622 https://clinicaltrials.gov/ct2/show/NCT04834622?term=shaper-pnd&draw=2&rank=1 (date of registration – April 8th 2021)

Key words: implementation-effectiveness; hybrid type 2 trial; Parkinson's disease; Postnatal depression; implementation science; scaling up; sustainability; arts interventions.

Word Count: 4000 words.

Strengths and limitations of the study

- SHAPER-Implement is the largest known study of its kind, comprising multidisciplinary implementation and evaluation teams, with consistent stakeholder engagement embedded throughout
- The study allows large-scale psychometric validation of newly developed implementation measures
- Provides an example of how large-scale hybrid studies can be conducted within community settings using a synergistic methodology with broad applicability
- PD-Ballet and M4M will be trialled in specific geographic areas in England, further assessment of the interventions across England will be required to assess the wider benefit.

For peer review only

For peer review only - http://bmjopen.bmj.com/site/about/guidelines.xhtml

Introduction

The use of arts interventions (i.e. 'creative methods of expression such as drama, music and visual arts'[1]) to improve health and social care outcomes and reduce service utilisation costs is an internationally growing area of research[1-4]. In 2017, the United Kingdom's All-Party Parliamentary Group on Arts, Health and Wellbeing (APPGAHW) published a report on the benefits of arts interventions, alongside ten stakeholder-led recommendations (from patients, health and social care professionals, artists, academics, charities, policy-makers and parliamentarians) on facilitating the implementation and scale-up of 'arts' into health and social care systems nationally[1]. Two years on, the World Health Organisation's scoping review of the global academic literature (2000-2019)[2] identified over 900 publications, including 200 reviews, systematic reviews, meta-analyses and meta-syntheses covering over 3000 studies, and 700 additional studies. Taken the evidence collectively, arts interventions are an effective method to help treat a plethora of physical, social and psychological problems across the lifespan[1-4].

Despite the promising evidence, progress on successfully embedding art interventions in health and social care systems has been slow. Presently, many 'arts' are delivered in small geographic or healthcare pockets, operating at the fringe of the care sector rather than receiving mainstream funding[1, 5]. While lack of sustainable funding, weak partnerships with commissioners and unclear referral pathways partly account for this[1, 5] research is required to establish cost-effective, scalable solutions so that the full benefits to the wider population can be reached.

The current protocol reports the design and evaluation of the 'Scale-up of Health-Arts Programmes Effectiveness-Implementation Research' (SHAPER-Implement) – part of a larger programme (referred to as 'SHAPER'[6]) aimed at investigating ways to implement and deliver arts interventions at scale. We focus on two different health conditions: postnatal depression (PND) and Parkinson's disease (PD). These represent a significant fiscal and public health burden and pose considerable affliction on the individuals affected (and, where applicable, their carers)[7-14]. While pharmacological treatments can be effective for controlling/alleviating symptoms[7, 9], they are fraught with challenges: for PND, it is poor uptake and adherence[15-18], while for PD, it is overemphasis on treating the motor symptoms at the expense of non-motor functioning[19-21].

Melodies for Mums (M4M) (for mums with PD) and PD-Ballet (for individuals with PD) are two approaches to symptom management, that have been piloted with promising results[22-30]. Both have

For peer review only - http://bmjopen.bmj.com/site/about/guidelines.xhtml

already been implemented across certain locations in London, UK, but are not being delivered at scale, thus only reach a fraction of eligible individuals.

We plan to scale-up M4M/PD-Ballet and examine how we can embed them into clinical pathways so that a greater number of individuals can benefit. Our ambition is to be as inclusive as possible, reaching out to individuals who may not be undergoing treatment for their condition (as well as those that are), and ultimately for Clinical Commissioning Groups (e.g. the "payers" in the National Health Service, UK) to commission the interventions so they can be delivered in a sustainable way beyond the end of our research.

The entire SHAPER programme centres on tripartite objectives. Due to the complexity of the study design, this protocol reports the implementation effectiveness evaluation (i.e. the SHAPER-Implement programme) of M4M/PD-Ballet (objectives 2 and 3, described below). The clinical effectiveness evaluation (objective 1) is reported in parallel protocols[31, 32].

Objective 1: to assess the *clinical effectiveness* of M4M/PD-Ballet – described in detail in the clinical protocols[31, 32]

Objective 2: to examine the *implementation effectiveness* of M4M/PD-Ballet, including uptake, adoption, perceived acceptability, appropriateness, feasibility, fidelity, unintended consequences, and sustainability, and the impact of established implementation strategies (e.g., training in the delivery of M4M/PD-Ballet) on implementation effectiveness;

Objective 3: to assess *implementation costs and cost effectiveness*, including costs associated with implementing M4M/PD-Ballet into existing care pathways, health service, partner organisations and commissioning, costs to service users attending M4M/PD-Ballet versus the benefit in terms of quality-adjusted years of life lived (QALYs), and the impact of M4M/PD-Ballet when delivered at scale, on the wider utilisation of health care and other services.

Methods and analysis

Design

SHAPER-Implement is a two-arm effectiveness-implementation hybrid type 2[33] trial of M4M/PD-Ballet. Randomisation will be single-blinded (assessments performed by a blinded rater) and in a 2:1 ratio. M4M participants will receive the singing programme (intervention) or be encouraged to

For peer review only - http://bmjopen.bmj.com/site/about/guidelines.xhtml

attend non-music classes in the community or online (control). PD-Ballet participants will receive a dance programme (dance-based training and a post-session Tea-and-Biscuit social time) or follow the standard treatment per the local pathway (control) and attend the post-training 'Tea-and-Biscuit' gatherings via an online platform.

Table 1 details how M4M/PD-Ballet meet the criteria proposed by Curran et al[33] for a hybrid type 2 trial. Contextual constraints meant it was not feasible to randomise the implementation side of the trial (i.e. allocate participants to different implementation strategies): but we will examine the effectiveness of the implementation strategies used to deploy M4M/PD-Ballet.

Setting

SHAPER-Implement is a multisite, multidisciplinary, community-based study in London, UK. Funded by the Wellcome Trust, it is a collaboration between the Centre for Implementation Science, King's College London; King's Health Partners; the Department for Behavioural Science and Health and the Institute of Mental Health, University College London, and two award-winning arts organisations: Breathe Arts Health Research and English National Ballet (ENB). King's holds a long-established commitment to embedding arts, health and wellbeing in education and research[34, 35] and was the research partner for landmark publications in the area[1].

The health conditions

PND is a serious and the most common perinatal mental health condition, affecting 10-20% of women in pregnancy and after birth[11]. If left untreated the impact on women and their families can be devastating[11, 36, 37] with symptoms including fatigue, anhedonia, insomnia and irritability and thoughts of suicide[7, 8, 37,38]. Therapy (e.g. cognitive-behavioural therapy) or medication can be prescribed to help treat PND but progress can be slow, may involve long wait times for treatment (for therapy) or prolonged use of medication[39-41]- thus other options for symptom management need to be explored.

For peer review only - http://bmjopen.bmj.com/site/about/guidelines.xhtml

PD is one of the world's fastest growing chronic neurodegenerative disorders, with those aged over 70 being particularly vulnerable[14, 42, 43]. It affects over 145,000 people in the UK alone[43] with prevalence rates expected to rise by around 18% between 2018 and 2025 (to over 168,000), and doubling by 2065[10, 43]. Symptoms relate to motor (e.g. tremor, bradykinesia, freezing of gait) and non-motor (e.g. sleep disturbance, drooling, cognitive decline)[9, 10] functioning. Currently, there is no cure for PD – and the disease has a progressive course[9, 10]. Pharmacological treatments can alleviate symptoms and improve quality of life but are largely aimed at addressing motor functioning, leaving the non-motor symptoms often unrecognised or under-treated[10, 44, 45]. Alternative non-pharmacotherapies are necessary to slow down disease progression[10, 44, 45].

The interventions

Drawing from Curran et al's 'hybrid' framework[33] (see Table 1) through an intensive four-month stakeholder engagement with artists, researchers, clinicians, and commissioners, we considered five elements critical to assessing the relevance of the interventions:

- **Suitability** – M4M/PD-Ballet address a need within the health sector – there are growing patient populations with the conditions and lack of effective current service provision.
- **Quality and face validity** – M4M/PD-Ballet are 'high-quality' interventions with carefully designed and tailored activities, developed in partnership between centres of academic excellence and renowned arts organisations.
- **Inclusivity** – M4M/PD-Ballet are 'all-inclusive'. They have good uptake, not only with those already engaged in the arts. They also appear to 'reach-out' to individuals that are disengaged in other forms of treatment for PND/PD (e.g., pharmacological approaches).
- **Effectiveness** – there is evidence to show M4M/PD-Ballet improves symptoms and quality of life and can achieve better adherence than pharmacological approaches[23-26, 28-30].
- **Scalability** – M4M/PD-Ballet are not overly complex, thus have the potential to be scaled-up, embedded in clinical pathways and commissioned by the health sector[6].

Insert Table 1 here

For peer review only - http://bmjopen.bmj.com/site/about/guidelines.xhtml
For peer review only

M4M[6, 31] is delivered in partnership with Breathe Arts Health Research (https://breatheahr.org/melodies-for-mums/). A Breathe-trained artist (the workshop leader) and support assistant deliver ten weekly singing sessions to a group of mums with PND and their babies. Classes start with welcome songs, followed by music activities, ranging from short vocal exercises and simple lullabies to longer songs that attendees learn gradually over the weeks. Songs can be relaxing, with mothers encouraged to hug/stroke their babies as they sing, or energetic, with mothers standing and moving with their babies. Instruments (e.g. guitars and ukuleles) are used by the workshop leader, accompanied by simpler instruments for mothers/babies to use. Mothers are encouraged to write their own songs, developing lyrics together relating to their babies or experiences of motherhood[6, 31]. Recordings of the singing sessions are made available to attendees at the end.

M4M has been subject to a three-arm randomised controlled trial (RCT) of 134 mothers and a preliminary process evaluation. Significantly faster improvements in symptoms for mothers with moderate-severe PND (measured by the Edinburgh Postnatal Depression Scale (EPDS)), than mothers in usual care[24] were observed. Levels of depression consistently declined - by week 6, 65% of mothers no longer had an EPDS $\geq$ 13, (i.e., indicating no more than mild depression); increasing to 73% by week 10[24]. Increase in the frequency of mothers singing to their babies outside the classes, perceived mother-infant closeness and a greater decrease in cortisol levels when compared to social play were also reported[23-25]. The process evaluation showed that M4M reached the correct target demographic, was delivered with a high level of fidelity and programme satisfaction was high - 88% of mothers agreed the classes were well tailored, and 100% would recommend M4M to another mother[26, 29, 30]. Several challenges, however, were highlighted by mothers, workshop leaders and the project coordinator (e.g., timing sessions with babies' routines) – whilst this did not hinder the continuation of M4M, it nonetheless suggests that implementation of M4M could be improved[26].

PD-Ballet[6, 32] is based on the ENB's pre-existing 'Dance for Parkinson' (DfP) programme (referred to as PD-Ballet for the SHAPER research) and delivered in partnership with the ENB (https://www.ballet.org.uk/project/dance-for-parkinsons/). Led by an ENB trained DfP Associate Artist and Associate Musician who deliver twelve weekly sessions to individuals with PD (carers/relatives can also attend), sessions comprise live music, dance and vocal exercises. Each session comprises seventy-five minutes of activity, followed by social time and refreshments (up to one hour) so that participants can get to know fellow attendees and form social networks. Content, inspired by the ENB's classical and contemporary works, provides a framework for participants to

For peer review only - http://bmjopen.bmj.com/site/about/guidelines.xhtml

explore narrative, themes, concepts, and music to promote freedom of expression. A performance sequence is developed at the end of PD-Ballet, combining all elements of the programme. Dance material is adapted to be inclusive (catering for differing levels of mobility) so that everyone can participate fully. For PD-Ballet content will be further developed for the three distinct stages of motor advancement (mild/moderate/severe) – something which has not been done before within the existing DfP programme. Prior to the programme, individuals attend an introductory session with their assigned group and are given the opportunity to attend a ballet performance and a behind the-scenes event[6, 32].

Initial testing of PD-Ballet in London, and its regional affiliated hub partners, has proven to be replicable, resulting in it being trialled and established in four other locations in the UK (Oxford, Cardiff, Ipswich and Liverpool). An independent evaluation led by the University of Roehampton (on scaling-up the programme, experiences and benefits of participation across four years and the effects on the body, daily activities, and social participation) reported high levels of perceived value from an emotional, social and artistic perspective[28]. Additionally, PD-Ballet can decrease social isolation and improve quality of life[22], with participants highly motivated and viewing PD-Ballet as an important part of their lives[27]. Equally, while recent systematic reviews and studies on the effects of performing acts modalities (including dance and ballet) reported promising benefits on Parkinson's symptoms ([46, 47])very few RCTs have investigated the benefits of 'dance' in both motor and non-motor symptoms, pointing to the need for this research.

SHAPER-Implement will build on previous research - conducting a full RCT (for PD-Ballet), a follow-up RCT scaled to a larger number of mothers (for M4M), and a process evaluation and examination of the implementation and potential cost-effectiveness of delivery at scale (for both) (see Table 2 for further details on M4M/PD-Ballet).

Insert Table 2 here

Implementation and adaption - COVID-19 pandemic

As a result of the first national lockdown in the United Kingdom on 23rd March 2020 and the guidelines that followed, M4M/PD-Ballet were adapted to be delivered online and preliminary

For peer review only - http://bmjopen.bmj.com/site/about/guidelines.xhtml

research into the feasibility of using an online platform is underway. While the plan moving forward (i.e., when the trials begin) will be to deliver PD-Ballet and M4M face-face, this will be continually reviewed based on the government guidelines on COVID-19 at the time, and the switch to remote delivery of the programmes will be made if necessary.

Theoretical underpinning

The Medical Research Council's (MRC) framework for evaluating complex interventions[48, 49] informed the study design for intervention development, implementation and evaluation processes.

The 'Implementation Science Research Development' (ImpRes) tool[50,] allowed us to operationalise the MRC guidance into the overall design of SHAPER-Implement, along with the research team's knowledge and experience of conducting hybrid trials[51]. We also tailored intervention delivery using the COM-B model[52] by considering factors that could affect individuals' capability, opportunity and motivation to engage with M4M/PD-Ballet.

The Consolidated Framework for Implementation Research (CFIR)[53, 54] will be used to assess factors affecting the implementation M4M/PD-Ballet. The 'Expert Recommendations for Implementing Change' (ERIC) project's compendium of implementation strategies[55, 56] will guide the identification of implementation strategies that could overcome some of the factors affecting implementation.

The Reach Effectiveness Adoption Implementation Maintenance (RE-AIM) framework[57, 58], which comprises domains on 'implementation' and 'effectiveness' and is suited to address challenges of blended effectiveness-implementation designs, guided the selection of implementation measures alongside Proctor et al[44] taxonomy of implementation outcomes.

Participants

For M4M, 400 mothers (with their babies) will be randomised 2:1 to the intervention (N=270) versus control (N=130). Mothers over 18 years of age with symptoms of PND (defined as a diagnosis of major depressive disorder according to the Structured Clinical Interview for DSM-IV

For peer review only - http://bmjopen.bmj.com/site/about/guidelines.xhtml

Disorders and a score of ≥ 10 on the EPDS) and a baby 0-9 months old, will be eligible. Mothers with an EPDS score of < 10 will be excluded and signposted to other support services within the community (e.g. mother-baby groups) but invited to re-screen for future cycles of the intervention (in case their symptoms change).

For PD-Ballet, 160 individuals will be recruited: 2:1 to the intervention (N=107) versus control (N=53). Participants will be 18 years old or over with a diagnosis of idiopathic PD according to the UK PD Brain Bank criteria and Hoehn Yarhr stages I-V. Individuals with a diagnosis of other parkinsonism or an indication of dementia through a score of ≤21 on the Montreal Cognitive Assessment (MoCA) will be excluded.

Individuals (for both M4M/PD-Ballet) will also be excluded if they are unable to: understand English; give informed consent; access and attend the singing/dance sessions online (due to COVID-19) through an internet-connected device (e.g. mobile phone, computer), or face-to-face (government guidelines permitting).

Individuals who decline participation or withdraw from M4M/PD-Ballet will be asked (providing they are willing) to provide their reason(s), so that barriers to uptake (from 'decliners') or continued participation (from 'withdrawers') can be identified and addressed.

SHAPER-Implement will also collect data on stakeholders' attitudes and experiences of M4M/PD-Ballet (N=25-30, respectively) so that challenges relating to intervention delivery can be reviewed and overcome.

A description of power and sample size calculations for M4M/PD-Ballet can be found in the corresponding clinical protocols[31, 32].

Patient and public involvement

We are engaging with members of the public and patients throughout SHAPER-Implement and at every stage of the research process. We have formed advisory groups comprising individuals who have already completed the PD-Ballet and M4M programme. To date, these individuals have provided feedback on (but not limited to) our study design, data collection measures, recruitment strategies and methods for disseminating findings.

For peer review only - http://bmjopen.bmj.com/site/about/guidelines.xhtml

Wider stakeholder engagement

In addition to patients/the public, engagement with other stakeholders is also critical to the success and scalability of M4M/PD-Ballet. We will hold regular meetings with: 'deliverers' – artists/staff involved in delivering M4M/PD-Ballet; 'referrers' – those who refer individuals to M4M/PD-Ballet, (e.g. general practitioners or neurologists (PD-Ballet); psychiatrists, health visitors and midwives (M4M) and; 'supporters' – additional staff who support the running of M4M/PD-Ballet. To-date, examples of involvement include contributing to the overall study design; critically reviewing methods of assessment for relevance and clarity; and exploring and facilitating referral pathways for recruitment.

Developing theory of change

We will conduct stakeholder workshops to develop theory of change (ToC) and logic models. The ToC element will focus on summarising M4M/PD-Ballet at a strategic level, capturing possible pathways for the change process to achieve our outcomes. The logic models will focus on the programme specifics, assessing the change process at the level of implementing M4M/PD-Ballet. We will advance the traditional logic model by drawing from the recently developed Implementation Research Logic Model[59]. We will examine inputs (e.g. time), activities (e.g. recruitment), outputs (e.g. publications), outcomes (e.g. symptoms), implementation determinants (identified in the CFIR[53, 54]), implementation strategies, (drawing from the ERIC project[55, 56]) and mechanisms of action resulting from the implementation outcomes (informed by the implementation outcome taxonomy[60] and RE-AIM)[57, 58].

Recruitment

Recruitment for M4M will be through: 1) signposting via health and social care professionals, including midwives and health visitors; 2) healthcare referrals, including General Practitioners, clinical psychologists, psychiatrists and self-referral; 3) weighing clinics and other community and

For peer review only - http://bmjopen.bmj.com/site/about/guidelines.xhtml

clinical centres for postnatal mothers, and; 4) social media groups and online forums aimed at new mothers (for an example of the consent form, please refer to Suppl. file 1)

Recruitment for PD-Ballet will be through: 1) the Movement Disorders Outpatient Clinic at King's College Hospital; 2) London South Parkinson's Excellence Network (PEN) using the London South PEN patient section of the website; 3) London South and National Clinical Research Network (CRN) websites and the EUROPAR website https://parkinsons-london.co.uk/europar/; 4) the Study Hub website (Parkinson's UK) and; 5) the ENB.

Stakeholders will be recruited through Breathe's (for M4M) and ENB's (for PD-Ballet) network of artists, staff and others involved in M4M/PD-Ballet.

The start and end dates for the trials are September 2021–December 2023 (for M4M) and February 2022-December 2022 (for PD-Ballet). Follow-up data will be collected at 36 weeks so the full data collection for the trials will end in May 2024 (for M4M) and May 2023 (for PD-Ballet). It should be noted that the more than one cycle of each programme will run for each trial, and that these cycles may overlap (i.e., occur simultaneously) to varying degrees for M4M/PD-Ballet – hence the slightly different durations of the each of the trials.

Measures

Measures will be standardised across M4M/PD-Ballet, piloted and further refined (where applicable) through a co-design process with stakeholders to ensure their suitability.

Data on the acceptability[61] of M4M/PD-Ballet (primary implementation outcome measure) and training in the delivery of M4M/PD-Ballet (SHAPER-Implement's established implementation strategy), and data on the feasibility[61] and appropriateness[61] will be collected at 3 timepoints.

Implementation costs will be assessed using a previously developed costing proforma[62, 63] on the time and financial resource associated with implementing M4M/PD-Ballet within existing care pathways. To explore commissioning costs, feedback from provider organisations will be sought (those responsible for providing the interventions) to establish a range of plausible unit prices that the NHS/partner organisations would be required to pay. To establish the impact of M4M/PD-Ballet on the wider utilisation of services (i.e. other health and service costs), service utilisation

For peer review only - http://bmjopen.bmj.com/site/about/guidelines.xhtml

questionnaires on the frequency of contact with other NHS/non-NHS services over a 3-month retrospective period will be completed. Reported service use will be costed using published unit cost estimates for health care and other services. The EQ5D 3L[64] will be used to evaluate quality of life outcomes and quality-adjusted life year outcomes in the short-term.

To explore sustainment of M4M/PD-Ballet, the 'Normalisation Measure Development' questionnaire (NOMAD)[65] will be administered post-intervention.

Enrolment onto M4M/PD-Ballet will assess uptake and participation data and attrition rates will be used to assess adherence.

Sociodemographic data on participants and stakeholders will be collected (see Table 3 for further details on measures).

Insert Table 3 here

Qualitative measures

Semi-structured interviews partly informed by the study team's methods of assessment for previous hybrid trials[51, 63] will complement the quantitative findings. Interviews will occur post-intervention and examine:

- Implementation strategies used to deliver M4M/PD-Ballet alongside strategies that may be important to consider by the local centres wishing to implement M4M/PD-Ballet after completion of SHAPER-Implement.
- Factors affecting the perceived acceptability, appropriateness, and feasibility of M4M/PD-Ballet.
- Fidelity of delivery – whether M4M/PD-Ballet is delivered in accordance with protocol (e.g., number and length of sessions, format and content covered).
- Fidelity of receipt - how the content of M4M/PD-Ballet is received by participants, including their experiences of engagement, comprehension of the content delivered, and application of acquired knowledge and skills to daily life.
- Factors affecting the sustained use of the knowledge and skills acquired from M4M/PD-Ballet for the long-term management of PND/PD (from participants) and the

For peer review only - http://bmjopen.bmj.com/site/about/guidelines.xhtml

implementation of M4M/PD-Ballet into local services after the completion of SHAPER-Implement (from stakeholders).

- Unintended consequences (positive and negative) of M4M/PD-Ballet
- Stakeholders' willingness for continued involvement in M4M/PD-Ballet (i.e. intention to adopt) and participants' intentions to use the knowledge and skills gained from M4M/PD-Ballet and recommend M4M/PD-Ballet to others.

Data Analysis

Quantitative implementation outcomes

Parametric and nonparametric tests will be employed to compare survey responses between the two arms of the trial for M4M/PD-Ballet and the established implementation strategy (i.e. training arts leads/artists). Linear, logistic and Poisson regression models (depending on the distribution of the outcome) will explore the relationship between implementation (AIM, IAM and FIM[61]) and clinical trial primary (EPDS[66] for M4M and MDS-MNS[67] for PD-Ballet) and secondary outcome. Potential moderators (e.g. age of the age of the baby for M4M) of the effect of implementation (AIM, IAM and FIM) on clinical trial primary (EPDS[66] for M4M and MDS-MNS[67] for PD-Ballet) and secondary outcome will be explored with the inclusion of an interaction term. Mediation analysis using Structural Equation Models under the causal framework will be employed to understand the potential pathways in which implementation outcomes and the implementation strategy of 'training', impact on the effectiveness of M4M/PD-Ballet[68, 69]. All analyses will be conducted in STATA 16.0.

Psychometric assessment of the reliability, validity, and factorial structure of the implementation survey scales

The implementation outcomes scales are relatively new, hence require psychometric assessment. Cronbach's coefficient alpha will evaluate the reliability (internal consistency) of the IAM, FIM and AIM scale-items (values range from 0-1 with internal consistency deemed acceptable when Cronbach's alpha[70] is ≥ 0.70[71]). A confirmatory factor analysis (CFA) model will be fitted, using the weighted least square estimator with a mean-and-variance-adjusted chi-squared method to handle ordered categorical items[72]. To evaluate overall model fit, the comparative fit index (CFI)[73],

For peer review only - http://bmjopen.bmj.com/site/about/guidelines.xhtml

the Tucker Lewis index (TLI)[74] and the root mean square error of approximation (RMSEA)[75] will be calculated. A CFI and TFI value of > 0.90 indicates adequate fit to the data[76]. A value of RMSEA <0.05 indicates close fit, values between 0.05-0.08 suggest adequate model fit, and values >0.10 suggest poor model fit[76]. Psychometric analysis will be conducted using STATA 16 and Mplus 7.4[77].

Health economic outcomes

Using a combination of empirical and modelling methods, we will bring together evidence on clinical endpoints, quality-of life, implementation and intervention costs, and epidemiological data on prevalence and service utilisation to gauge the cost-effectiveness of delivering M4M/PD-Ballet at scale. We will build in evidence-based assumptions regarding the longer-term health and service resource impacts beyond the observable trial period. The cost-effectiveness of scalability under differing scenarios will be assessed with reference to existing thresholds, inclusive of those currently used by the National Institute for Health and Care Excellence (NICE) for assessing programme cost-effectiveness. Uncertainty in estimates will be explored through probabilistic and one-way sensitivity analysis. Cost-effectiveness will be evaluated from an NHS perspective, with a focus on exploring the incidence of incremental resource impacts associated with utilisation of NHS funded resources.

Qualitative implementation outcomes

A systematic classification process of coding themes/patterns in the data will be used with the main themes predetermined deductively by the Proctor et al[60] implementation outcomes framework and RE-AIM[78]. Sub-themes will be shaped inductively with the data until saturation is achieved. CFIR[79, 80] will be used to analyse barriers/facilitators (anticipated and actual) to the implementation and sustainment of M4M/PD-Ballet. NVivo 12 will be used for data analysis.

Ethics and dissemination

For peer review only - http://bmjopen.bmj.com/site/about/guidelines.xhtml

M4M and PD-Ballet have been reviewed and approved by the West London and GTAC Research Ethics Committee (Reference: 20/PR/0813) and the HRA and Health and Care Research Wales Research Ethics Committee (Reference: 20/WA/0261). Informed consent will be sought from all research participants. The results will be disseminated in academic journals and conferences as well as other channels (including to patients and the public in the centres and healthcare settings where the programmes are being delivered and through social media).

For peer review only

List of abbreviations

AIM = Acceptability Intervention Measure

APPGAHW = All-Party Parliamentary Group on Arts, Health and Wellbeing

CFA = Confirmatory item Factor Analysis

CFI = Comparative Fit Index

CFIR = Consolidated Framework for Implementation Research

CRN = Clinical Research Network

PD-BALLET = Dance for Parkinson's

DSM-IV = Diagnostic and Statistical Manual of Mental Disorders, Fourth Edition

For peer review only - http://bmjopen.bmj.com/site/about/guidelines.xhtml

ENB = English National Ballet

EPDS = Edinburgh Postnatal Depression Scale

ERIC = Expert Recommendations for Implementing Change

FIM = Feasibility Intervention Measure

GP = General Practitioner

IAM = Intervention Appropriateness Measure

ImpRes = Implementation Science Research Development

M4M = Melodies for Mums

MoCA = Montreal Cognitive Assessment

MRC = Medical Research Council

NHS = National Health Service

NICE = National Institute for Health and Care Excellence

NMSS = Non-Motor Symptoms Scale

NoMAD = Implementation measure based on Normalization Process Theory

PD = Parkinson's Diseases

PEN = Parkinson's Excellence Network

PND = Postnatal Depression

QALYs = Quality-adjusted years of life lived

RE-AIM = Reach Effectiveness Adoption Implementation Maintenance

RMSEA = Root mean square error of approximation

SCID = Structured Clinical Interview for DSM-IV

SHAPER = Scale-up of Health-Arts Programmes Effectiveness-implementation Research

TLI = Tucker Lewis index

ToC = Theory of change

Declarations

For peer review only - http://bmjopen.bmj.com/site/about/guidelines.xhtml

Ethics approval and consent to participate

SHAPER-PND (the trial on the M4M program for individuals with PND) has been reviewed and given favourable opinion by the West London and GTAC Research Ethics Committee (REC Reference: 20/PR/0813). SHAPER-PD (the trial on the PD-BALLET program for individuals with PD) has been reviewed and given favourable opinion by the HRA and Health and Care Research Wales Research Ethics Committee (REC Reference: 20/WA/0261). Informed consent will be sought from all research participants for this study. The study will be conducted in accordance with the Good Clinical Practice and recommendations for physicians involved in research on human subjects adopted by the 18th World Medical Assembly, Helsinki 1964 and later revisions.

Consent for publication

Not applicable.

Availability of data and material

Not applicable, this manuscript does not contain any data.

Competing interests

NS is the Director of London Safety & Training Solution ltd, which offers training in patient safety, implementation solutions and human factors to healthcare organisations and the pharmaceutical industry. TS received funding from Cancer Alliances and Health Education England for training multidisciplinary teams in assessment and quality improvement methods in the United Kingdom. TS received consultancy fees from Roche Diagnostics in relation to implementation of innovations for multidisciplinary teams and their meetings in the United States of America. DF is a non-executive board director for Breathe Arts Health Research. But she receives no financial compensation for her involvement. CP has received research funding from Johnson & Johnson for research on depression and inflammation, and by a Wellcome Trust strategy award to the Neuroimmunology of Mood Disorders and Alzheimer's Disease (NIMA) Consortium (104025), which is also funded by Janssen, GlaxoSmithKline, Lundbeck and Pfizer. The work presented in this paper is unrelated to this funding. The remaining authors have no conflicts of interest to declare.

Funding

For peer review only

These trials included in SHAPER-Implement are part of the SHAPER programme, a Scaling-up Health-Arts Programme to scale up arts interventions. The programme is funded by the Wellcome Trust (award reference 219425/Z/19/Z).

IB, AH, and NS are funded by and RD is supported by the National Institute for Health Research (NIHR) via the 'Applied Research Collaboration South London' (NIHR ARC South London) at King's College Hospital National Health Service (NHS) Foundation Trust, London, UK. AH and NS are members of King's Improvement Science, which offers co-funding to the NIHR ARC South London and is funded by King's Health Partners (Guy's and St Thomas' NHS Foundation Trust, King's College Hospital NHS Foundation Trust, King's College London and South London and Maudsley NHS Foundation Trust), and Guy's and St Thomas' Foundation. IB (is further funded) and CP are supported by the NIHR Biomedical Research Centre at South London and Maudsley NHS Foundation Trust and CP is a NIHR Senior Investigator. MLB is further funded by Diabetes UK. RP is supported by HEartS, a project funded by the UK's Arts and Humanities Research Council to investigate the health, economic and social impact of the arts (Grant ref. AH/P005888/1).

The funding agreements ensured the authors' independence in designing the study, interpreting the data, writing, and publishing the report. The views expressed are those of the authors and not necessarily reflect those of the NHS, the Wellcome Trust, the NIHR, the charities or the Department of Health and Social Care.

Authors' contributions

RD, TS, MBL, AH, CE, DF, PD, CP, HD, TO, RB, KS, LR, KH, AB, MM, RP, AP, RC, FDF, AH, AW, NC, IB, NS (i.e. all authors provided input on the design of the study and its conceptualisation) RD, TS, NS, AH, CE, IB, MLB were responsible for its drafting and edits. All authors provided feedback on all aspects of the content of the manuscript and its methodology. All authors read and approved the final version of the manuscript.

Acknowledgements We would like to thank the representatives of the people with Parkinson's disease and postnatal depression, as well as the artists and health care professionals involved, for their time and commitment to the design of this study protocol.

For peer review only - http://bmjopen.bmj.com/site/about/guidelines.xhtml

References

1. All-Party Parliamentary Group on Arts. Health and wellbeing inquiry report. Creative health: the arts for health and wellbeing. All-Party Parliamentary Group on Arts; 2017.

2. Fancourt D, Finn S. What is the evidence on the role of the arts in improving health and well-being? A scoping review. WHO Regional Office for Europe; 2019.

3. Stuckey HL, Nobel J. The connection between art, healing, and public health: a review of current literature. *Am J Public Health.* 2010;100(2):254-63.

4. Boyce M, Bungay H, Munn-Giddings C, Wilson C. The impact of the arts in healthcare on patients and service users: A critical review. *Health Soc Care Community.* 2018;26(4):458-73.

5. Eccles MP, Mittman BS. Welcome to Implementation Science. *Implement Sci.* 2006;1(1).

6. Estevao C, Fancourt D, Dazzan P, Chaudhuri K, Sevdalis N, Woods A; et al. Scaling-up Health-Arts Programmes: the largest study in the world bringing mental health and arts into a national health service. *J B Psych Bulletin.* [in press].

7. Boath E, Henshaw C. The treatment of postnatal depression: A comprehensive literature review. *J Reprod Infant Psychol.* 2001;19(3):215-48.

8. Morrell CJ, Warner R, Slade P, Dixon S, Walters S, Paley G, et al. Psychological interventions for postnatal depression: cluster randomised trial and economic evaluation. The PoNDER trial. *Health Tech Asses* 2009;13(30):1-153.

9. National Institute for Health Care Excellence. Parkinson's disease in adults: National Institute for Health and Care Excellence; 2017.

10. UK Parkinson's. UK Parkinson's audit - Transforming care. UK Parkinson's; 2017.

11. Bauer A, Parsonage M, Knapp M, Iemmi V, Adelaja B. The costs of perinatal mental health problems. Centre for Mental Health and London School of Economics; 2014.

12. Parkinson's UK. The cost of Parkinson's: the financial impact of living with the condition. Parkinson's UK; 2017.

13. Soundy A, Stubbs B, Roskell C. The Experience of Parkinson's Disease: A Systematic Review and Meta-Ethnography. *Sci World J.* 2014;2014:613592.

14. Yang W, Hamilton JL, Kopil C, Beck JC, Tanner CM, Albin RL, et al. Current and projected future economic burden of Parkinson's disease in the U.S. *Parkinson's Dis.* 2020;6(1):15.

For peer review only - http://bmjopen.bmj.com/site/about/guidelines.xhtml

15. Bledsoe SE, Grote NK. Treating Depression During Pregnancy and the Postpartum: A Preliminary Meta-Analysis. *Res Soc Work Pract.* 2006;16(2):109-20.

16. Cooper PJ, Murray L, Wilson A, Romaniuk H. Controlled trial of the short- and long-term effect of psychological treatment of post-partum depression. I. Impact on maternal mood. *Br J Psychiatry.* 2003;182:412-9.

17. Milgrom J, Negri LM, Gemmill AW, McNeil M, Martin PR. A randomized controlled trial of psychological interventions for postnatal depression. *Br J Clin Psychol.* 2005;44(Pt 4):529-42.

18. Cooper PJ, Murray, L., & Halligan, S. L. Treatment of postpartum depression. Winnicott Research Unit, University of Reading, UK: Centre of Excellence for Early Childhood Development; 2010.

19. Booth FW, Roberts CK, Laye MJ. Lack of exercise is a major cause of chronic diseases. *Compr Physiol.* 2012;2(2):1143-211.

20. Chaudhuri KR, Titova N. Societal burden and persisting unmet needs of Parkinson's disease. *Eur Neurol Rev.* 2019;14(1):28-35.

21. Martinez-Martin P, Rodriguez-Blazquez C, Kurtis MM, Chaudhuri KR. The impact of non-motor symptoms on health-related quality of life of patients with Parkinson's disease. *Mov Disord.* 2011;26(3):399-406.

22. Bognar S, DeFaria AM, O'Dwyer C, Pankiw E, Simic Bogler J, Teixeira S, et al. More than just dancing: experiences of people with Parkinson's disease in a therapeutic dance program. *Disabill Rehabil.* 2017;39(11):1073-8.

23. Fancourt D, Perkins R. Associations between singing to babies and symptoms of postnatal depression, wellbeing, self-esteem and mother-infant bond. *Public Health.* 2017;145:149-52.

24. Fancourt D, Perkins R. Effect of singing interventions on symptoms of postnatal depression: three-arm randomised controlled trial. *Br J Psychiatry.* 2018;212(2):119-21.

25. Fancourt D, Perkins R. The effects of mother–infant singing on emotional closeness, affect, anxiety, and stress hormones. *Music Sci.* 2018;1.

26. Fancourt D, Perkins R. Creative interventions for symptoms of postnatal depression: A process evaluation of implementation. *Arts Health.* 2019;11(1):38-53.

27. Houston S, McGill A. A mixed-methods study into ballet for people living with Parkinson's. *Arts & health.* 2013;5(2):103-19.

28. Cultural Commissioning Programme Case Study. English National Ballet Dance for Parkinson's: Harnessing the power of dance to improve Health & Wellbeing. 2015.

For peer review only - http://bmjopen.bmj.com/site/about/guidelines.xhtml

29. Fancourt D, Perkins R. Does attending community music interventions lead to changes in wider musical behaviours? The effect of mother–infant singing classes on musical behaviours amongst mothers with symptoms of postnatal depression. *Psychol Music.* 2017;47(1):132-43.

30. Perkins R, Yorke S, Fancourt D. How group singing facilitates recovery from the symptoms of postnatal depression: a comparative qualitative study. *BMC Psychol.* 2018;6(1):41.

31. Estevao C, Bind R, Fancourt D, Burton A, Sawyer K, Hazelgrove K, et al. The SHAPER-PND trial: clinical effectiveness protocol of a community singing intervention for postnatal depression *BMJ Open* [in press].

32. Podlewska A, Davis R, Soukup T, Sevdalis N, Baldellou Lopez M, Bakolis I, et al. Design of the PD-Ballet study: a randomised controlled single-blind hybrid type 2 clinical trial evaluating the effects of ballet dancing on motor and non-motor symptoms in Parkinson's disease. [in preparation].

33. Curran GM, Bauer M, Mittman B, Pyne JM, Stetler C. Effectiveness-implementation hybrid designs: combining elements of clinical effectiveness and implementation research to enhance public health impact. *Med Care.* 2012;50(3):217-26.

34. King's Cultural Community. Arts, health & wellbeing: King's College London [Available from: https://www.kcl.ac.uk/cultural/arts-health-wellbeing].

35. King's Cultural Community. Arts-enhanced health education [Available from: https://www.kcl.ac.uk/cultural/arts-health-wellbeing/education].

36. Grigoriadis S, Wilton AS, Kurdyak PA, Rhodes AE, VonderPorten EH, Levitt A, et al. Perinatal suicide in Ontario, Canada: a 15-year population-based study. *Cmaj.* 2017;189(34):1085-92.

37. Slomian J, Honvo G, Emonts P, Reginster J-Y, Bruyère O. Consequences of maternal postpartum depression: A systematic review of maternal and infant outcomes. *Journal of Women's health* 2019;15.

38. Pearlstein T, Howard M, Salisbury A, Zlotnick C. Postpartum depression. *Am J Obstet Gynecol.* 2009;200(4):357-64.

39. Alison C, Scott S. Pharmacologic and Hormonal Treatments for Postpartum Depression. *Psychiatr Ann.* 2005;35(5):568-76.

40. Pearlstein T, Howard M, Salisbury M, Zlotnick C. Postpartum depression. *Am J Obstet Gynecol.* 2009;200(4):357-64.

41. Becki H. NHS England's Access and Waiting Times Programme Perinatal Mental Health. 2015. p. http://www.swscn.org.uk/wp/wp-content/uploads/2015/12/-07-31-presentation-NHSE-perinatal-programme.pdf.

For peer review only - http://bmjopen.bmj.com/site/about/guidelines.xhtml

42. Yang G, Schmiel L, Zhou M, Cintina I, Hogan P. Economic Burden and Future Impact of Parkinson's Disease

Final Report. The Lewin Group; 2019.

43. Parkinson's UK. The incidence and prevalence of Parkinson's in the UK. Parkinson's UK; 2018.

44. Kelberman MA, Vazey EM. New Pharmacological Approaches to Treating Non-Motor Symptoms of Parkinson's Disease. *Curr Pharmacol Rep.* 2016;2(6):253-61.

45. Zahoor I, Shafi A, Haq E. Pharmacological Treatment of Parkinson's Disease. Parkinson's Disease: Pathogenesis and Clinical Aspects. Brisbane Codon Publications; 2018.

46. Barnish MS, Barran SM. A systematic review of active group-based dance, singing, music therapy and theatrical interventions for quality of life, functional communication, speech, motor function and cognitive status in people with Parkinson's disease. *BMC Neurology.* 2020;20(1):371.

47. McGill A, Houston S, Lee RYW. Effects of a ballet-based dance intervention on gait variability and balance confidence of people with Parkinson's. *Arts & Health.* 2019;11(2):133-46.

48. Craig P, Dieppe P, Macintyre S, Michie S, Nazareth I, Petticrew M. Developing and evaluating complex interventions: the new Medical Research Council guidance. *BMJ.* 2008;337:a1655.

49. Craig P, Dieppe P, Macintyre S, Michie S, Nazareth I, Petticrew M. Developing and evaluating complex interventions: the new Medical Research Council guidance. *Int J Nurs Stud.* 2013;50(5):587-92.

50. Hull L, Goulding L, Khadjesari Z, Davis R, Healey A, Bakolis I, et al. Designing high-quality implementation research: development, application, feasibility and preliminary evaluation of the implementation science research development (ImpRes) tool and guide. *Implement Sci.* 2019;14(1):80.

51. Soukup T, Hull L, Smith EL, Healey A, Bakolis I, Amiel SA, et al. Effectiveness-implementation hybrid type 2 trial evaluating two psychoeducational programmes for severe hypoglycaemia in type 1 diabetes: implementation study protocol. *BMJ Open.* 2019;9(11):303-70.

52. Michie S, van Stralen MM, West R. The behaviour change wheel: a new method for characterising and designing behaviour change interventions. *Implement Sci.* 2011;6:42-.

53. Consolidated Framework for Implementation Research. 2021 [Available from: https://cfirguide.org/].

54. Damschroder LJ, Aron DC, Keith RE, Kirsh SR, Alexander JA, Lowery JC. Fostering implementation of health services research findings into practice: a consolidated framework for advancing implementation science. *Implement Sci.* 2009;4(1):50.

For peer review only - http://bmjopen.bmj.com/site/about/guidelines.xhtml

55. Powell BJ, Waltz TJ, Chinman MJ, Damschroder LJ, Smith JL, Matthieu MM, et al. A refined compilation of implementation strategies: results from the Expert Recommendations for Implementing Change (ERIC) project. *Implement Sci.* 2015;10(1):21.

56. Waltz TJ, Powell BJ, Matthieu MM, Damschroder LJ, Chinman MJ, Smith JL, et al. Use of concept mapping to characterize relationships among implementation strategies and assess their feasibility and importance: results from the Expert Recommendations for Implementing Change (ERIC) study. *Implement Sci.* 2015;10(1):109.

57. RE-AIM 2021 [Available from: https://www.re-aim.org/].

58. Glasgow RE, Vogt TM, Boles SM. Evaluating the public health impact of health promotion interventions: the RE-AIM framework. *Am J Public Health.* 1999;89(9):1322-7.

59. Smith JD, Li DH, Rafferty MR. The Implementation Research Logic Model: a method for planning, executing, reporting, and synthesizing implementation projects. *Implement Sci.* 2020;15(1):84.

60. Proctor E, Silmere H, Raghavan R, Hovmand P, Aarons G, Bunger A, et al. Outcomes for implementation research: conceptual distinctions, measurement challenges, and research agenda. *Adm Policy Ment Health.* 2011;38(2):65-76.

61. Weiner BJ, Lewis CC, Stanick C, Powell BJ, Dorsey CN, Clary AS, et al. Psychometric assessment of three newly developed implementation outcome measures. *Implement Sci.* 2017;12(1):108.

62. Soukup T HL, Smith EL, Rogers H, Healey A, Ioannis B, Amiel S, Sevdalis N, PWD Group. The role, value and challenges of stakeholder involvement in implementation research. *BMJ Q&S.* [in press].

63. Soukup T, Hull L, Healey A, Bakolis I, Smith E, Amiel S, et al. Interview topic guides exploring implementation outcomes across stakeholder groups (Version 2) Zenodo; 2020 [Available from: http://doi.org/10.5281/zenodo.3865707].

64. Herdman M, Gudex C, Lloyd A, Janssen M, Kind P, Parkin D, et al. Development and preliminary testing of the new five-level version of EQ-5D (EQ-5D-5L). *Qual Life Res.* 2011;20(10):1727-36.

65. Finch TG, M; May, CR; Mair, FS; Murray, E; Treweek, S; Steen, IN; McColl, EM; Dickinson, C; Rapley, T. NoMad: Implementation measure based on Normalization Process Theory. 2015 [Available from: http://www.normalizationprocess.org].

66. Cox JL, Holden JM, Sagovsky R. Detection of Postnatal Depression: Development of the 10-item Edinburgh Postnatal Depression Scale. *Br J Psychiatry.* 1987;150(6):782-6.

For peer review only - http://bmjopen.bmj.com/site/about/guidelines.xhtml

67. Martinez-Martin P, Chaudhuri KR, Rojo-Abuin JM, Rodriguez-Blazquez C, Alvarez-Sanchez M, Arakaki T, et al. Assessing the non-motor symptoms of Parkinson's disease: MDS-UPDRS and NMS Scale. *Eur J Neurol.* 2015;22(1):37-43.

68. De Stavola BL, Daniel RM, Ploubidis GB, Micali N. Mediation Analysis With Intermediate Confounding: Structural Equation Modeling Viewed Through the Causal Inference Lens. *American Journal of Epidemiology.* 2015;181(1):64-80.

69. R. K. Principles and practice of structural equation modeling. 3rd ed. New York: Guilford press; 2011.

70. Cronbach LJ. Coefficient alpha and the internal structure of tests. *Psychometrika.* 1951;16(3):297-334.

71. DeVellis R. Scale development: Theory and applications: Sage publications; 2016.

72. Muthén B, du Toit S, Spisic D. Robust inference using weighted least squares and quadratic estimating equations in latent variable modeling with categorical and continuous outcomes. *Psychometrika.* 1997;75(1):40-5.

73. Bentler PM. Comparative fit indexes in structural models. *Psychol Bull.* 1990;107(2):238-46.

74. Tucker LR, Lewis C. A reliability coefficient for maximum likelihood factor analysis. *Psychometrika.* 1973;38(1):1-10.

75. Steiger JH. Statistically-Based Tests for the Number of Common Factors. 1980.

76. Hu Lt, Bentler PM. Cutoff criteria for fit indexes in covariance structure analysis: Conventional criteria versus new alternatives. *Struct Equ Modeling.* 1999;6(1):1-55.

77. Muthén LK, Muthén BO. Mplus User's Guide. 5th ed. Los Angeles, CA: Muthén & Muthén; 1998-2007.

78. Harden S, Smith M, Ory M, Smith-Ray R, Estabrooks P, Glasgow R. Re-aim in clinical, community, and corporate settings: perspectives, strategies, and recommendations to enhance public health impact. *Front Public Health.* 2018.

79. Damschroder LJ, Hagedorn HJ. A guiding framework and approach for implementation research in substance use disorders treatment. *Psychol Addict Behav.* 2011;25(2):194-205.

80. Kirk MA, Kelley C, Yankey N, Birken SA, Abadie B, Damschroder L. A systematic review of the use of the Consolidated Framework for Implementation Research. *Implement Sci.* 2016;11(1):72.

For peer review only

For peer review only - http://bmjopen.bmj.com/site/about/guidelines.xhtml

For peer review only

For peer review only - http://bmjopen.bmj.com/site/about/guidelines.xhtml

Table 1. Overview of the Curran et al[33] criteria for hybrid trial type 2 design and how they apply to M4M and PD-Ballet hybrid evaluations

Study Characteristics for Hybrid Trial Type 2, as per Curran et al[33] criteria		Trialled Arts Interventions	
		**Melodies for Mums**	**PD-Ballet**
Research aims	**Determine feasibility and potential utility of an implementation intervention/strategy**<br><br>*Determine effectiveness of a clinical intervention	**Determine feasibility and potential utility of M4M to facilitate future implementation and scale up**<br><br>Determine clinical effectiveness of M4M	**Determine feasibility and potential utility of PD-Ballet to facilitate future implementation and scale up**<br><br>Determine clinical effectiveness of PD-Ballet
Research questions	**Does the implementation method show promise (either alone or in comparison with another method) in facilitating implementation of a clinical treatment?**<br><br>*Will a clinical treatment work in these settings/ for these patients?	**Will the implementation method show promise (alone) in facilitating M4M in people with PND?**<br><br>What is the clinical impact of M4M?	**Will the implementation method show promise (alone) in facilitating PD-Ballet in people with PD?**<br><br>What is the clinical impact of PD-Ballet?

For peer review only - http://bmjopen.bmj.com/site/about/guidelines.xhtml

Unit of randomization	**Provider, clinical unit, facility or system, as per type although may be nonrandomized, e.g., case study**<br><br>Patient or clinical unit, as per type 1	**Providers, as per type 3 (non-randomised)**<br><br>Patients, as per type 1 (randomised)	**Providers, as per type 3 (non-randomised)**<br><br>Patients, as per type 1 (randomised)
Comparison conditions	**Provider, clinical unit, facility, system: implementation as usual, or competing implementation strategy although may be nonrandomized, e.g., case study**<br>Placebo, treatment as usual, or competing treatment, as per type 1	**Facility, as per hybrid type 3**<br><br>Treatment as usual, as per hybrid type 1	**Facility, as per hybrid type 3**<br><br>Treatment as usual, as per hybrid type 1
Sampling frames	**Providers/clinics/facility/systems: consider "optimal" cases**<br><br>Patient: limited restrictions, but some inclusion/exclusion criteria	**Providers/facility: artists, psychiatrists/neurologists, referrers, commissioners, support staff; community centres**<br><br>Patient: inclusion/exclusion criteria used	**Providers/facility: artists, psychiatrists/neurologists, referrers, commissioners, support staff; dance centres**<br><br>Patient: inclusion/exclusion criteria used

For peer review only - http://bmjopen.bmj.com/site/about/guidelines.xhtml

Evaluation methods	**Mixed method; quantitative, qualitative; formative and summative**<br><br>Quantitative, summative	**Quantitative surveys (§AIM,[61] ǁIAM[61], ¶FIM,[61] **NoMaD,[65] costing proforma) and interviews[63]**<br><br>Quantitative surveys (†EPDS[66]; EQ-5D 3L; [64])	**Quantitative surveys (§AIM,[61] ǁIAM[61], ¶FIM,[61] **NoMaD,[65] costing proforma) and interviews[63]**<br><br>Quantitative surveys (†EPDS[66]; EQ-5D 3L; [64])
Measures	**Adoption of clinical treatment and fidelity to it, as well as related factors**<br><br>Patient symptoms and functioning, possibly cost effectiveness	**Adoption of M4M, fidelity of its delivery and receipt, acceptability, appropriateness, feasibility, sustainability, reach, unintended consequences, contextual factors, implementation strategies, implementation costs**<br><br>Patient symptoms and functioning, cost effectiveness	**Adoption of PD-Ballet, fidelity of its delivery and receipt, acceptability, appropriateness, feasibility, sustainability, reach, unintended consequences, contextual factors, implementation strategies, implementation costs**<br><br>Patient symptoms and functioning, cost effectiveness

Note. *Curran et al: "one of these aims/research questions might take precedence, for example in a case where the test of an implementation intervention/strategy is exploratory".[33] †EPDS=Edinburgh Postnatal Depression Scale. ‡NMSS=Non-Motor Symptoms Scale. §AIM=Acceptability of Intervention Measure. ǁIAM=Intervention Appropriateness Measure. ¶FIM=Feasibility of Intervention Measure. **NoMaD=Implementation measure based on Normalization Process Theory.

Bold type = information relating to the implementation effectiveness evaluation. Normal type = information relating to the clinical effectiveness evaluation, discussed in more detail in a separate protocols[31,32]

Table 2. Overview of the structure and delivery of Melodies for Mums and PD-Ballet interventions

For peer review only - http://bmjopen.bmj.com/site/about/guidelines.xhtml

Programme structure and delivery	Melodies for Mums	PD-Ballet
Arts partner	Breathe Arts Health Research	English National Ballet
Targeted health condition	Postnatal depression	Parkinson's disease
Setting	Community children's centres OR online (depending on COVID-19 guidelines)	Community dance studios OR online (depending on COVID-19 guidelines)
Delivery structure	A weekly 1-hour group session over 10 weeks	A weekly 2-hour 15-minute 12 group session over 12 weeks
N of intervention cycles	10 cycles, with a morning and afternoon cycle held across 5 separate10-weekly periods	3 cycles (stratified by motor advancement – mild/moderate/severe)
N of participants per cohort	12-15	30-35
Total *N* of participants in the intervention	270 mums (plus their respective babies)	107 individuals with Parkinson's*
Follow-ups (as related to the delivery of sessions)	No follow-up	No follow-up
Artists per session	2 (1 artist, 1 assistant)	4 (2 associate dance artists / 2 associate musicians), plus 1 *artist-in-training*
Total *N* of participants in the control	*N*=130 mums	*N*=53

Note. N=number. *The three cohorts differ in the severity of Parkinson's symptoms: mild, moderate, and severe ** The 2 days training is required for each stage of motor advancement so the trainer would need to attend three 2-day training sessions if they running programs for all 3 groups of motor advancement (mild/moderate/severe).

- All those involved in M4M and PD-Ballet will receive training in a standardised format beforehand the delivery of the programmes.

Table 3. Data collection plan for the implementation effectiveness evaluation of Melodies for Mums and PD-Ballet interventions

**Outcome**	**Definition**	**Form of measurement**	**Timepoint(s)**	**Stakeholder group**
**Implementation effectiveness**				
Acceptability	Extent to which M4M/PD-Ballet are perceived to be agreeable and acceptable for management of PND and PD, respectively.<br><br>Extent to which training in the delivery of M4M/PD-Ballet (i.e., SHAPER-Implement's established implementation strategy) is considered acceptable to arts leads/artists	AIM survey<br><br>Interview	During intervention, post intervention and 3-6 months post-intervention follow-up	Participants, and wider stakeholder groups
Appropriateness	Extent to which M4M/PD-Ballet are perceived to be fit and relevant for management of PND and PD, respectively.	IAM survey<br><br>Interview	During intervention, post intervention and 3-6 months post-intervention follow-up	Participants, and wider stakeholder groups
Feasibility	Extent to which M4M/PD-Ballet can be successfully used or carried out to help manage and improve PND and PD-related effects.	FIM survey<br><br>Interview	During intervention, post intervention and 3-6 months post-intervention follow-up	Participants, and wider stakeholder groups
Fidelity of delivery	Extent to which M4M/PD-Ballet sessions were delivered according to protocol.	Interview	Post intervention	Deliverers
Fidelity of receipt	Extent to which M4M/PD-Ballet are received as intended.	Interview	Post intervention	Participants
Adoption	Intention to adopt and use the knowledge and skills learned in M4M/PD-Ballet in everyday management of PND and PD.	Interview	Post intervention	Participants, and wider stakeholder groups

For peer review only - http://bmjopen.bmj.com/site/about/guidelines.xhtml

Sustainability	Facilitators and barriers to sustained use of M4M/PD-Ballet.	Interview and the NoMad survey	Post-intervention and follow up	Participants, and wider stakeholder groups
Uptake	The proportion and representativeness of individuals willing to participate in M4M/PD-Ballet.	Trial records	Baseline	Provided by trial manager
Adherence	The number of individuals attending each M4M/PD-Ballet session and the number of dropouts	Trial records	Throughout intervention period	Provided by trial manager
Unintended consequences	Positive or negative consequences that are not anticipated at the time of implementation of M4M/PD-Ballet.	Interview	Post intervention	Participants, and wider stakeholder groups
Implementation strategies*	Strategies used to deliver and implement M4M/PD-Ballet.	Interview	Post intervention	Wider stakeholder groups
**Implementation costs and cost effectiveness**				
Implementation costs	Costs associated with prospective implementation of M4M/PD-Ballet.	Costing proforma	Post intervention	Participants, and wider stakeholder groups
Cost-benefit analysis	Costs associated with M4M/PD-Ballet versus the benefits in terms of the outcomes.	EQ-5D 3L	During and post intervention	Participants
**Clinical effectiveness****				
	The impact of M4M/PD-Ballet on the clinical outcomes of interest.	EPDS survey<br>NMSS survey	Baseline, post intervention and 3-6 months post-intervention follow-up	Participants

Note. PD=Parkinson's Disease. PND=Post-natal Depression. AIM=Acceptability of Intervention Measure. IAM=Intervention Appropriateness Measure. FIM=Feasibility of Intervention Measure. EPDS=Edinburgh Postnatal Depression Scale (Melodies for Mums). NMSS=Non-Motor Symptoms Scale (Dance for Parkinson's). NoMAD=Implementation measure based on Normalization Process Theory.

For peer review only - http://bmjopen.bmj.com/site/about/guidelines.xhtml

- As part of SHAPER-Implement we will be assessing the acceptability and benefit of ‘training arts leads/artists’ in the delivery of M4M/PD-ballet (i.e. our established implementation strategy) but we will also be examining other implementation strategies that may be of use in the wider-scale implementation of M4M/PD-Ballet.

** For further information on the clinical effectiveness evaluations, please refer to the corresponding clinical protocols for M4M[31] and PD-Ballet[32].

For peer review only

For peer review only - http://bmjopen.bmj.com/site/about/guidelines.xhtml

King's College London

NHS South London and Maudsley NHS Foundation Trust

CONSENT FORM FOR PARTICIPANTS IN RESEARCH STUDIES

Please complete this form after you have read the Information Sheet and/or listened to an explanation about the research.

<u>Title of Study:</u> SHAPER-PND: Community singing interventions for postnatal depression: a hybrid type II effectiveness-implementation trial

Thank you for considering taking part in this research. One of the researchers from the research team will explain the project to you before you agree to take part. If you have any questions arising from the Information Sheet or explanation already given to you, please ask the researcher before you decide whether you should join in. You will be given a copy of this Consent Form to keep and refer to
at any time.

Your information may be subject to review by responsible individuals from the research team for screening, monitoring and audit purposes.

Confidentiality will be maintained and you will not be identified in any research outputs.

	Yes	No
**I confirm that I understand that by ticking/initialling a 'Yes' box, I am consenting to being involved in this element of the study. I confirm that I understand that by ticking/initialling a 'No' box, I DO NOT consent to being involved in this element of the study.**	☐	☐
1. I confirm that I have read and understood the information sheet dated [30-Jul-2021, version 2.1] for the above study. I have had the opportunity to consider the information and asked questions which have been answered to my satisfaction.	☐	☐
2. I consent voluntarily for myself and for my baby to be a participant in this study and understand that I can withdraw from the study at any time, without having to give a reason.	☐	☐
3. I consent to the processing of my personal information for the purposes explained to me in the Information Sheet. I understand that such information will be handled confidentially, in accordance with the terms of the General Data Protection Regulation.	☐	☐
4. I agree that the research team may use my data for future research within and outside the EU and understand that any such use of identifiable data would be reviewed and approved by a research ethics committee. In such cases, as with this project, data will not be identifiable in any report.	☐	☐
5. I agree to be contacted in the future by King's College London researchers who would like to invite me to participate in follow up studies to this project, or in future studies of a similar nature.	☐	☐

For peer review only - http://bmjopen.bmj.com/site/about/guidelines.xhtml

6. I agree to provide details of my GP and understand that if any safeguarding issues arise, my GP might be contacted to discuss the best way to support me and my baby. ☐ ☐

7. I agree to record short videos being taken of play interaction in a comfortable setting between my baby and me. This recording aims to look at how me and my baby interact with each other and the information obtained can be used for our research. ☐ ☐

8. I agree to my views being shared in focus groups and subsequent interviews to be recorded (in audio and/or video format) for data collection. The researchers may use a transcription service to transcribe the recordings into a different format. I understand that my identity will stay anonymous and I will not be identifiable in the published data or materials. (optional) ☐ ☐

9. I agree to provide biological samples (saliva) throughout the study in accordance to the study protocol. (optional) ☐ ☐

10. I agree to provide biological samples (hair) throughout the study in accordance to the study protocol. (optional) ☐ ☐

11. My biological samples may be used for future studies and my data will remain anonymous. (optional) ☐ ☐

12. I agree to provide biological samples (saliva only) of my baby in accordance to the study protocol. (optional) ☐ ☐

13. The biological samples of my baby may be used for future studies and the data will remain anonymous. (optional) ☐ ☐

14. I agree to be contacted by implementation science researchers to provide my views on the study. I understand that my views may be video and audio recorded and published but I will not be identifiable in any of the research outputs. (optional) ☐ ☐

15. I do not wish to participate in the study, but I agree to be contacted by implementation science researchers for a brief interview, to explain why. (optional) ☐ ☐

Note that you must consent to points 1-7 in order to be eligible for the study. Points 8-14 are not mandatory but are still an integral part of the study. If you do not wish to participate in the study but consent to being contacted to tell us why (optional), just tick point 15.

Participant:

Your Name	Date	Signature

Researcher:

Name of Researcher	Date	Signature

For peer review only - http://bmjopen.bmj.com/site/about/guidelines.xhtml

STANDARD PROTOCOL ITEMS: RECOMMENDATIONS FOR INTERVENTIONAL TRIALS

SPIRIT 2013 Checklist: Recommended items to address in a clinical trial protocol and related documents*

Section/item	Item No	Description	Addressed on page number
**Administrative information**			
Title	1	Descriptive title identifying the study design, population, interventions, and, if applicable, trial acronym	__1___________
Trial registration	2a	Trial identifier and registry name. If not yet registered, name of intended registry	__2/3_________
	2b	All items from the World Health Organization Trial Registration Data Set	__NA______
Protocol version	3	Date and version identifier	__NA________
Funding	4	Sources and types of financial, material, and other support	____19______
Roles and responsibilities	5a	Names, affiliations, and roles of protocol contributors	___1&20_______
	5b	Name and contact information for the trial sponsor	___3 & 18______
	5c	Role of study sponsor and funders, if any, in study design; collection, management, analysis, and interpretation of data; writing of the report; and the decision to submit the report for publication, including whether they will have ultimate authority over any of these activities	________NA____
	5d	Composition, roles, and responsibilities of the coordinating centre, steering committee, endpoint adjudication committee, data management team, and other individuals or groups overseeing the trial, if applicable (see Item 21a for data monitoring committee)	____NA_______

For peer review only

Introduction

Background and rationale	6a	Description of research question and justification for undertaking the trial, including summary of relevant studies (published and unpublished) examining benefits and harms for each intervention	__4-5_______
	6b	Explanation for choice of comparators	__5, 7-9________
Objectives	7	Specific objectives or hypotheses	____4-5______
Trial design	8	Description of trial design including type of trial (eg, parallel group, crossover, factorial, single group), allocation ratio, and framework (eg, superiority, equivalence, noninferiority, exploratory)	____7-9_______

Methods: Participants, interventions, and outcomes

Study setting	9	Description of study settings (eg, community clinic, academic hospital) and list of countries where data will be collected. Reference to where list of study sites can be obtained	_____6_______
Eligibility criteria	10	Inclusion and exclusion criteria for participants. If applicable, eligibility criteria for study centres and individuals who will perform the interventions (eg, surgeons, psychotherapists)	_______10-11__
9Interventions	11a	Interventions for each group with sufficient detail to allow replication, including how and when they will be administered	_______7-9____
	11b	Criteria for discontinuing or modifying allocated interventions for a given trial participant (eg, drug dose change in response to harms, participant request, or improving/worsening disease)	_______9_____
	11c	Strategies to improve adherence to intervention protocols, and any procedures for monitoring adherence (eg, drug tablet return, laboratory tests)	_______11-12__
	11d	Relevant concomitant care and interventions that are permitted or prohibited during the trial	___NA_______
Outcomes	12	Primary, secondary, and other outcomes, including the specific measurement variable (eg, systolic blood pressure), analysis metric (eg, change from baseline, final value, time to event), method of aggregation (eg, median, proportion), and time point for each outcome. Explanation of the clinical relevance of chosen efficacy and harm outcomes is strongly recommended	12-14, 25-26, 28-30
Participant timeline	13	Time schedule of enrolment, interventions (including any run-ins and washouts), assessments, and visits for participants. A schematic diagram is highly recommended (see Figure)	_____12______

For peer review only - http://bmjopen.bmj.com/site/about/guidelines.xhtml

Sample size	14	Estimated number of participants needed to achieve study objectives and how it was determined, including clinical and statistical assumptions supporting any sample size calculations	______10-11____
Recruitment	15	Strategies for achieving adequate participant enrolment to reach target sample size	_______12_____

Methods: Assignment of interventions (for controlled trials)

Allocation:

Sequence generation	16a	Method of generating the allocation sequence (eg, computer-generated random numbers), and list of any factors for stratification. To reduce predictability of a random sequence, details of any planned restriction (eg, blocking) should be provided in a separate document that is unavailable to those who enrol participants or assign interventions	____5-6______
Allocation concealment mechanism	16b	Mechanism of implementing the allocation sequence (eg, central telephone; sequentially numbered, opaque, sealed envelopes), describing any steps to conceal the sequence until interventions are assigned	__5-6_______
Implementation	16c	Who will generate the allocation sequence, who will enrol participants, and who will assign participants to interventions	___5-6____
Blinding (masking)	17a	Who will be blinded after assignment to interventions (eg, trial participants, care providers, outcome assessors, data analysts), and how	_____NA_____
	17b	If blinded, circumstances under which unblinding is permissible, and procedure for revealing a participant's allocated intervention during the trial	______NA_____

Methods: Data collection, management, and analysis

Data collection methods	18a	Plans for assessment and collection of outcome, baseline, and other trial data, including any related processes to promote data quality (eg, duplicate measurements, training of assessors) and a description of study instruments (eg, questionnaires, laboratory tests) along with their reliability and validity, if known. Reference to where data collection forms can be found, if not in the protocol	12-15, 25-26, 28-3
	18b	Plans to promote participant retention and complete follow-up, including list of any outcome data to be collected for participants who discontinue or deviate from intervention protocols	12-15, 25-26, 28-30

Data management	19	Plans for data entry, coding, security, and storage, including any related processes to promote data quality (eg, double data entry; range checks for data values). Reference to where details of data management procedures can be found, if not in the protocol	___14-15______
Statistical methods	20a	Statistical methods for analysing primary and secondary outcomes. Reference to where other details of the statistical analysis plan can be found, if not in the protocol	___14-15______
	20b	Methods for any additional analyses (eg, subgroup and adjusted analyses)	___14-15_______
	20c	Definition of analysis population relating to protocol non-adherence (eg, as randomised analysis), and any statistical methods to handle missing data (eg, multiple imputation)	__14-15________
**Methods: Monitoring**			
Data monitoring	21a	Composition of data monitoring committee (DMC); summary of its role and reporting structure; statement of whether it is independent from the sponsor and competing interests; and reference to where further details about its charter can be found, if not in the protocol. Alternatively, an explanation of why a DMC is not needed	_______NA______
	21b	Description of any interim analyses and stopping guidelines, including who will have access to these interim results and make the final decision to terminate the trial	_____NA______
Harms	22	Plans for collecting, assessing, reporting, and managing solicited and spontaneously reported adverse events and other unintended effects of trial interventions or trial conduct	_______NA_____
Auditing	23	Frequency and procedures for auditing trial conduct, if any, and whether the process will be independent from investigators and the sponsor	_____NA______
**Ethics and dissemination**			
Research ethics approval	24	Plans for seeking research ethics committee/institutional review board (REC/IRB) approval	___16, 18______
Protocol amendments	25	Plans for communicating important protocol modifications (eg, changes to eligibility criteria, outcomes, analyses) to relevant parties (eg, investigators, REC/IRBs, trial participants, trial registries, journals, regulators)	_____18______

Consent or assent	26a	Who will obtain informed consent or assent from potential trial participants or authorised surrogates, and how (see Item 32)	_____12, 16_____
	26b	Additional consent provisions for collection and use of participant data and biological specimens in ancillary studies, if applicable	_______NA____
Confidentiality	27	How personal information about potential and enrolled participants will be collected, shared, and maintained in order to protect confidentiality before, during, and after the trial	____12, 16______
Declaration of interests	28	Financial and other competing interests for principal investigators for the overall trial and each study site	________18____
Access to data	29	Statement of who will have access to the final trial dataset, and disclosure of contractual agreements that limit such access for investigators	_____18_____
Ancillary and post-trial care	30	Provisions, if any, for ancillary and post-trial care, and for compensation to those who suffer harm from trial participation	____NA_______
Dissemination policy	31a	Plans for investigators and sponsor to communicate trial results to participants, healthcare professionals, the public, and other relevant groups (eg, via publication, reporting in results databases, or other data sharing arrangements), including any publication restrictions	_____16_______
	31b	Authorship eligibility guidelines and any intended use of professional writers	_______20______
	31c	Plans, if any, for granting public access to the full protocol, participant-level dataset, and statistical code	_____NA______
**Appendices**			
Informed consent materials	32	Model consent form and other related documentation given to participants and authorised surrogates	________NA____
Biological specimens	33	Plans for collection, laboratory evaluation, and storage of biological specimens for genetic or molecular analysis in the current trial and for future use in ancillary studies, if applicable	_______NA_____

*It is strongly recommended that this checklist be read in conjunction with the SPIRIT 2013 Explanation & Elaboration for important clarification on the items. Amendments to the protocol should be tracked and dated. The SPIRIT checklist is copyrighted by the SPIRIT Group under the Creative Commons "Attribution-NonCommercial-NoDerivs 3.0 Unported" license.

For peer review only - http://bmjopen.bmj.com/site/about/guidelines.xhtml
