# Supplementary material for: Study protocol: randomised controlled hybrid type 2 trial evaluating the scale-up of two arts interventions for postnatal depression and Parkinson’s disease
Source: BMJ Open. 2022 Feb 1;12(2):e055691. doi: 10.1136/bmjopen-2021-055691 (PMC8808453; doi:10.1136/bmjopen-2021-055691)
Supplement: Reviewer comments [file bmjopen-2021-055691.reviewer_comments.pdf]

PEER REVIEW HISTORY

BMJ Open publishes all reviews undertaken for accepted manuscripts. Reviewers are asked to complete a checklist review form (**http://bmjopen.bmj.com/site/about/resources/checklist.pdf**) and are provided with free text boxes to elaborate on their assessment. These free text comments are reproduced below.

ARTICLE DETAILS

**TITLE (PROVISIONAL)**	Study protocol: Randomised controlled hybrid type 2 trial evaluating the scale-up of two arts interventions for Postnatal depression and Parkinson's disease
**AUTHORS**	Soukup, Tayana; Davis, Rachel; Baldellou Lopez, Maria; Healey, Andy; Estevao, Carolina; Fancourt, Daisy; Dazzan, Paola; Pariante, Carmine; Dye, Hannah; Osborn, Tim; Bind, Rebecca; Sawyer, Kristi; Rebecchini, Lavinia; Hazelgrove, Katie; Burton, Alexandra; Manoharan, Manonmani; Perkins, Rosie; Podlewska, Aleksandra; Chaudhuri, Ray; Derbyshire-Fox, Fleur; Hartley, Alison; Woods, Anthony; Crane, Nikki; Bakolis, Ioannis; Sevdalis, Nick

VERSION 1 – REVIEW

**REVIEWER**	Barnish, Max<br>University of Exeter, Exeter Medical School
**REVIEW RETURNED**	06-Aug-2021

**GENERAL COMMENTS**	I consider this to be an interesting area of research and an interesting study protocol, which is of generally high quality. I have some suggestions for improving the clarity of the write-up and also for improving the analysis. While I consider it appropriate to present these two trials together for the purposes of a protocol, it is likely to be more appropriate to present the study results separately, given the fact that the performing arts interventions for the two client groups are fundamentally different in nature.<br><br>1. Control groups are often a major issue in performing arts studies, as shown in the systematic review by Barnish and Barran (2020). While at this stage of study development, it is unlikely to be possible to improve the design of the control groups, it is important to note them as a limitation and to gain as much information as possible to inform the interpretation of the analysis of differences between intervention and control. In M4M, you say (p.5) that control participants will be 'encouraged' to attend non-music classes in the community. However, it will be important for interpretation to find out at the final data collection point whether or not control participants did participate in such classes, and if so what they were. If the classes were dance-based and involved music, but participants did not consider them 'music classes' this could confound the effect and reduce the observed benefit of M4M. With PD-Ballet, the control group is usual care. This is a common control group, but can be particularly problematic to define and interpret (Barnish and Barran, 2020). It will be important to seek information from clinical stakeholders in the study locations to find out - and indeed this can vary locally - what the 'usual' care control participants receive is likely to comprise.

	Otherwise, you are in effect comparing against a black box which makes interpretation very difficult.<br>2. Considering Table 2, it would be useful to clarify whether all clients in the same arm will receive sessions from leaders with equivalent professional background and level of expertise. The Barnish and Barran (2020) review showed that not only have previous PD performing arts studies used leaders with very different disciplinary backgrounds and levels of experience, which makes comparison of findings between studies very difficult, that in some studies different participants within the same arm were also taught by non-comparable leaders. This compromised the internal validity of these studies markedly. Therefore, it would be worthwhile in Table 2 or a footnote to it, to clarify this point in relation to your study.<br>3. It would be useful to clarify that and indeed how you intend to disseminate the findings to patient and clinical professional audiences - as this will be important in terms of encouraging patient uptake in community arts programmes, increasing clinician referral to existing services (arts groups both in the community and run through medical channels), and stimulating routine commissioning of additional services. Academic channels cannot be relied on to reach allied health audiences.<br>4. Please clarify the intended study dates and what has been done so far - my impression is that you are in the planning stage, but this is not made clear. We need to know when the study is intended to launch and when it is likely to end.<br>5. In terms of generalisability, it could be useful in the discussion to reflect on the challenges of generalising from London (it appears, if I have understood correctly - it could be made clearer in the manuscript - that the study is being run entirely in London with only London-based patients). The challenges of generalising from London to the wider UK could include social, economic, cultural, ethnic and urban vs rural factors.<br>6. In the M4M analysis, it would seem important to stratify (at least in a supplementary analysis) by age of baby, since 0 to 9 months is quite a broad spectrum and participant experiences and indeed what can be done in the sessions may differ quite markedly.<br>7. The key systematic review in recent years on performing arts and Parkinson's disease (Barnish and Barran, 2020) has not been cited. While this is my review, the reason I am mentioning it is not because it is mine, but that objectively it is the largest review to date of this field and the only review to cover a range of performing arts modalities, and explore a range of methodological challenges. Therefore, I think it is fair to say that without it, the references cannot be seen as up-to-date and appropriate. I provide the reference at the end of the review. You should also check that no key review in the field of performing arts and maternal health has been missed.<br>8. The authors claim that 'There has been no published research on ballet as an<br>intervention for PD, pointing to the need for this research' (p.9). This statement is incorrect. However, I do not believe that this in any way invalidates the rationale for the study. However, you do need to be aware of and cite previous research. The Barnish and Barran (2020) review profiles studies to date in the performing arts for PD. You need to bear in mind however that there could have been further studies since its publication, and I consider this a matter for the authors to check. Appendix 2 of Barnish and Barran (2020) profiles the interventions that have been studied so far. While not pure ballet, Batson (2014) used an intervention of

	improvisational dance seated at the ballet Barre and ambulating. This is clearly relevant, and should have been cited. Indeed, a lot of work on performing arts in PD adapts the art forms to meet the needs of the client group, resulting in adapted art forms. You should also cite studies using Dance for Parkinson's disease (Bearss, 2017; Clifford, 2017;Heiberger, 2011; Kalyani, 2019 and several others - all listed in Appendix 2 of Barnish and Barran, 2020, with full citations available in Appendix 8 of the same paper), which is an adapted PD-specific dance form that brings together different dance forms, and does borrow from ballet. Additionally, there is a prior study considering pure ballet on its own (McGill, 2019), which is a key study you need to consider in terms of situating your work.<br>9. Covid-19 related information appears out of date and does not reflect the latest 19 July 2021 situation in England. While there are no Covid restrictions legally applicable in England (which your manuscript might imply there are), it is fair to see the country has not yet returned fully to normality. Both client groups you are studying may indeed be seen as vulnerable. It is however important to empower patients and not decide for them what they are willing to do, it can be a very individual matter - hence the difficulty in classing someone as vulnerable. It is also going to be important to see whether the community groups you anticipate as a control arm have resumed and in what mode of delivery. It is possible that the resumption of normal service provision, especially in services provided through the health service, may lag considerably behind the removal of legal restrictions. In the manuscript, it is stated that online delivery may be used. It would be interesting to hear how piloting of online delivery is going both from a technical perspective and from a potential participant engagement perspective. My expertise in the performing arts literature is predominantly in adult neurological conditions such as Parkinson's disease, but at least within this remit I am not aware of any studies that have used online delivery. If the mode of delivery of intervention and control is not matched, this could be problematic. I am generally unconvinced that online delivery rather than a postponement would be the way to go if you do not feel you can proceed in person at present (although at the time of writing this review there is no legal reason why it could not proceed in person). The Barnish and Barran (2020) review focused on group based performing arts interventions for the very reason that the group dynamic is likely to be a key driver of benefit. In your manuscript you also state that "PD-Ballet can decrease social isolation and improve quality of life" (p.9). I would agree with you, but would suggest that these benefits are unlikely to be realised in a remote online setting. However, it would in a way be interesting from a mechanistic perspective to see whether benefits are upheld when the social component is removed or substantially altered. I would caution though that it is unlikely they would be and therefore the trial could turn out being wasted. There is at least anecdotal evidence I am aware of that when community arts groups such as choirs went online in 2020 user engagement dropped a lot. For M4M, the loss of social bonds at this challenging time of life and the loss of communal singing - reduced to singing alone on 'mute' to the conductor's backing tape - could undermine the intervention to the point of futility. Although it seems the ethics board has approved it, I would personally question the ethics of having an online-only study for Parkinson's, given the age profile and equity concerns (also potential generalisability issues if more affluent or educated senior citizens are more likely to be proficient on video

	conferencing software). Postponement until the study can be run in a way that would do it justice would seem by far the best option, if even after 19 July 2021, you do not feel you can run it in person in the near future (patient groups would be a key barometer of opinion as to whether this would be feasible). After all we are unlikely to be stuck in an online virtual world forever! If some participants do receive online intervention and some in-person, the analysis would need to be stratified by mode of delivery, at least as a supplementary analysis, as this could make quite a difference to effectiveness.<br><br>Key omitted references<br>Barnish MS, Barran SM. A systematic review of active group-based dance, singing, music therapy and theatrical interventions for quality of life, functional communication, speech, motor function and cognitive status in people with Parkinson's disease. BMC Neurol. 2020 Oct 10;20(1):371. doi: 10.1186/s12883-020-01938-3.<br><br>McGill, A., Houston, S., & Lee, R.Y.W. (2019). Effects of a ballet-based dance intervention on gait variability and balance confidence of people with Parkinson's. Arts and Health, 11(2): 133-146

**REVIEWER**	Bungay, Hilary<br>Anglia Ruskin University, Allied and Public Health
**REVIEW RETURNED**	10-Aug-2021

**GENERAL COMMENTS**	Thank you for submitting this protocol for review it is an interesting and well written protocol. I have a few minor points for consideration.<br>1. There are 25 authors listed as contributors – I do question if this is appropriate and whether the majority of these could be acknowledged for their contribution to the development of the study conception and design, rather than given authorship of this paper.<br>2. The dates of the study are not included in the protocol.<br>3. Limitations – the fact that this study appears to be London centric, and this is a potential limitation should be acknowledged, as implementation in other areas of the UK and internationally may face additional challenges.<br>4. Page 4 line 17 – remove 'clinically' or give a specific example in relation to physical and psychological problems.<br>5. P4 line 27 – the sentence starting 'While lacking…..' is unclear and depending on how it is read could seem contradictory.<br>6. P4 line 50 should be (for mums with PND), recommend removal of 'alternative (or conjunctive)' from this sentence.<br>7. Page 5 line 5 would 'purchasers' rather than 'payers' be more appropriate?<br>8. Page 5 line 53 this is inconsistent with Table 2 'Comparator method; which states 'standard treatment'. In addition, standard treatment would be a more appropriate comparator as recommending other similar activities could introduce confounding factors.<br>9. Table 1: These are not strictly Art Therapies – recommend re-labelling. Also there are errors in referencing under Evaluation methods – should this be reference 59 – rather then 61? Similarly, in the Measures are the interview references correct. Recommend references are checked carefully in this table

	10. P11 – as this is a protocol paper it would be useful to include the power calculations and sample sizes. This could be as a supplementary file if the word count is a limiting factor.<br>11. Page 12 – lines 3-6 developing a Theory of Change is usually undertaken prior to deciding on the measures/methods to use to determine whether the predicted outcomes have been achieved.

VERSION 1 – AUTHOR RESPONSE

Reviewer: 1. Dr. Max Barnish, University of Exeter

1. I consider this to be an interesting area of research and an interesting study protocol, which is of generally high quality. I have some suggestions for improving the clarity of the write-up and also for improving the analysis. While I consider it appropriate to present these two trials together for the purposes of a protocol, it is likely to be more appropriate to present the study results separately, given the fact that the performing arts interventions for the two client groups are fundamentally different in nature.

- Thank you very much for this feedback, we are delighted to hear you enjoyed reading the study. We completely agree with your point about writing up the results for each of the trials separately, and this is what we plan to do

2. Control groups are often a major issue in performing arts studies, as shown in the systematic review by Barnish and Barran (2020). While at this stage of study development, it is unlikely to be possible to improve the design of the control groups, it is important to note them as a limitation and to gain as much information as possible to inform the interpretation of the analysis of differences between intervention and control. In M4M, you say (p.5) that control participants will be 'encouraged' to attend non-music classes in the community. However, it will be important for interpretation to find out at the final data collection point whether or not control participants did participate in such classes, and if so what they were. If the classes were dance-based and involved music, but participants did not consider them 'music classes' this could confound the effect and reduce the observed benefit of M4M. With PD-Ballet, the control group is usual care. This is a common control group, but can be particularly problematic to define and interpret (Barnish and Barran, 2020). It will be important to seek information from clinical stakeholders in the study locations to find out - and indeed this can vary locally - what the 'usual' care control participants receive is likely to comprise. Otherwise, you are in effect comparing against a black box which makes interpretation very difficult.

- Thank you very much for this comment. We completely agree with this point and the importance of establishing exactly ‘what’ the control did, compared to the intervention group, because this (as you highlight) will affect the interpretation of the findings. We plan to discuss this issue in depth in the write up of the trials. Given this is a protocol paper we have no data on this yet. In addition, given that a discussion is not required in this paper because it is a protocol, we believe it is beyond the scope to discuss this here, but we will spend considerable time discussing these issues in the write-up of each of the trials.

3. Considering Table 2, it would be useful to clarify whether all clients in the same arm will receive sessions from leaders with equivalent professional background and level of expertise. The Barnish and Barran (2020) review showed that not only have previous PD performing arts studies used leaders with very different disciplinary backgrounds and levels of experience, which makes comparison of findings between studies very difficult, that in some studies different participants within the same arm were also taught by non-comparable leaders. This compromised the internal validity of these studies markedly. Therefore, it would be worthwhile in Table 2 or a footnote to it, to clarify this

point in relation to your study.

- Thank you for this comment. We agree that this will be an important point to discuss in the publications that we write up once the trials are complete. As part of this we will present the findings on which trainers delivered which sessions (and what their level of experience is). At this point we do not know for sure exactly who will be delivering each session because this could depend on availability (e.g., due to sickness, holidays) so we want to report on this when we actually ‘know’ – i.e. as part of the main findings in the trial write-ups.
- We should point out thought that all trainers will receive training in a standardised way before delivering PD-Ballet and M4M – we have included this as a footnote in the table. Again, further information on the specifics of the training will be included in the trial write-up.

4. It would be useful to clarify that and indeed how you intend to disseminate the findings to patient and clinical professional audiences - as this will be important in terms of encouraging patient uptake in community arts programmes, increasing clinician referral to existing services (arts groups both in the community and run through medical channels), and stimulating routine commissioning of additional services. Academic channels cannot be relied on to reach allied health audiences.

- A sentence on this has been added in the ‘ethics and dissemination’ section

5. Please clarify the intended study dates and what has been done so far - my impression is that you are in the planning stage, but this is not made clear. We need to know when the study is intended to launch and when it is likely to end.

- The study start and end dates have been added in the Methods section

6. In terms of generalisability, it could be useful in the discussion to reflect on the challenges of generalising from London (it appears, if I have understood correctly - it could be made clearer in the manuscript - that the study is being run entirely in London with only London-based patients). The challenges of generalising from London to the wider UK could include social, economic, cultural, ethnic and urban vs rural factors.

- Thank you for this – given this is a protocol paper, it doesn’t have a discussion (this isn’t a requirement of BMJ Open). We have also been asked by the editors to remove the conclusion section because we cannot base the conclusion on results. We very much agree however, with the importance of reflecting on the generalisability of findings and will certainly include information on this when we write up the trials.

7. In the M4M analysis, it would seem important to stratify (at least in a supplementary analysis) by age of baby, since 0 to 9 months is quite a broad spectrum and participant experiences and indeed what can be done in the sessions may differ quite markedly.

- Information on this has now been included in the analysis section.

8. The key systematic review in recent years on performing arts and Parkinson's disease (Barnish and Barran, 2020) has not been cited. While this is my review, the reason I am mentioning it is not because it is mine, but that objectively it is the largest review to date of this field and the only review to cover a range of performing arts modalities, and explore a range of methodological challenges. Therefore, I think it is fair to say that without it, the references cannot be seen as up-to-date and appropriate. I provide the reference at the end of the review. You should also check that no key review in the field of performing arts and maternal health has been missed.

- Thank you for this comment. We have now added this reference to the paper and agree it should be included. We have not been through all the other references in the review because this is a protocol paper that discusses two hybrid trials for differing populations. Therefore, while it is important for us to include relevant literature, it is beyond the scope of this paper and the aims of it (and indeed not possible due to word restrictions and us covering literature for two different evidence bases) to present a comprehensive account of all the literature to date because it is not a literature or systematic review.

9. The authors claim that 'There has been no published research on ballet as an intervention for PD, pointing to the need for this research' (p.9). This statement is incorrect. However, I do not believe that this in any way invalidates the rationale for the study. However, you do need to be aware of and cite previous research. The Barnish and Barran (2020) review profiles studies to date in the performing arts for PD. You need to bear in mind however that there could have been further studies since its publication, and I consider this a matter for the authors to check. Appendix 2 of Barnish and Barran (2020) profiles the interventions that have been studied so far. While not pure ballet, Batson (2014) used an intervention of improvisational dance seated at the ballet Barre and ambulating. This is clearly relevant, and should have been cited. Indeed, a lot of work on performing arts in PD adapts the art forms to meet the needs of the client group, resulting in adapted art forms. You should also cite studies using Dance for Parkinson's disease (Bearss, 2017; Clifford, 2017;Heiberger, 2011; Kalyani, 2019 and several others - all listed in Appendix 2 of Barnish and Barran, 2020, with full citations available in Appendix 8 of the same paper), which is an adapted PD-specific dance form that brings together different dance forms, and does borrow from ballet. Additionally, there is a prior study considering pure ballet on its own (McGill, 2019), which is a key study you need to consider in terms of situating your work.

- Thank you for this comment, as mentioned above it is beyond the scope of this protocol paper – which focuses on two arts interventions for two different conditions – to discuss all the literature of relevance. In the main write-ups of the trials, which will comprise two separate papers focusing on the results of each of the trials, we will discuss (and it will be possible to discuss) the literature in more detail. We have however cited your recent systematic review (see also our comment above on this), because we agree this is an important study to cite.

-

10. Covid-19 related information appears out of date and does not reflect the latest 19 July 2021 situation in England. While there are no Covid restrictions legally applicable in England (which your manuscript might imply there are), it is fair to see the country has not yet returned fully to normality. Both client groups you are studying may indeed be seen as vulnerable. It is however important to empower patients and not decide for them what they are willing to do, it can be a very individual matter - hence the difficulty in classing someone as vulnerable. It is also going to be important to see whether the community groups you anticipate as a control arm have resumed and in what mode of delivery. It is possible that the resumption of normal service provision, especially in services provided through the health service, may lag considerably behind the removal of legal restrictions. In the manuscript, it is stated that online delivery may be used. It would be interesting to hear how piloting of online delivery is going both from a technical perspective and from a potential participant engagement perspective. My expertise in the performing arts literature is predominantly in adult neurological conditions such as Parkinson's disease, but at least within this remit I am not aware of any studies that have used online delivery. If the mode of delivery of intervention and control is not matched, this could be problematic. I am generally unconvinced that online delivery rather than a postponement would be the way to go if you do not feel you can proceed in person at present (although at the time of writing this review there is no legal reason why it could not proceed in person). The Barnish and Barran (2020) review focused on group based performing arts interventions for the very reason that the group dynamic is likely to be a key driver of benefit. In your manuscript you also state that "PD-Ballet can decrease social isolation and improve quality of life" (p.9). I would agree with you, but

would suggest that these benefits are unlikely to be realised in a remote online setting. However, it would in a way be interesting from a mechanistic perspective to see whether benefits are upheld when the social component is removed or substantially altered. I would caution though that it is unlikely they would be and therefore the trial could turn out being wasted. There is at least anecdotal evidence I am aware of that when community arts groups such as choirs went online in 2020 user engagement dropped a lot. For M4M, the loss of social bonds at this challenging time of life and the loss of communal singing - reduced to singing alone on 'mute' to the conductor's backing tape - could undermine the intervention to the point of futility. Although it seems the ethics board has approved it, I would personally question the ethics of having an online-only study for Parkinson's, given the age profile and equity concerns (also potential generalisability issues if more affluent or educated senior citizens are more likely to be proficient on video conferencing software). Postponement until the study can be run in a way that would do it justice would seem by far the best option, if even after 19 July 2021, you do not feel you can run it in person in the near future (patient groups would be a key barometer of opinion as to whether this would be feasible). After all we are unlikely to be stuck in an online virtual world forever! If some participants do receive online intervention and some in-person, the analysis would need to be stratified by mode of delivery, at least as a supplementary analysis, as this could make quite a difference to effectiveness.

- Re: comment on COVID - In the section on ‘COVID’ we stated that because of the first national lockdown in March 2020 we started developing a way of delivering the interventions online, but we did not mean by this that the guidelines haven’t changed since then. After reading the text again we can see this could be a little unclear, so we have revised the text accordingly to avoid ambiguity.
- Re: vulnerable patients and empowering patients – we agree with the points raised – these will be examined as part of the trial and reported in the papers on the write-ups of the trials
- Re: online delivery – the feasibility of this is currently being assessed. We do not, as yet, have all on the data on this and it is beyond the scope of this protocol paper to report on this, but we are in the process of writing a paper on the feasibility of delivering PD-Ballet and M4M online. We agree (as you highlight) that it will be interesting to see to what extent social isolation is impacted when the interventions are delivered online and whether individuals still feel connected to others in the same way as in-person delivery. While we do not have the full dataset on the online feasibility study yet, initial results appear promising. There will of course be some individuals that prefer F-F delivery, but there may also be others that cannot attend in person due, for example, due to mobility issues, so providing them with a different way of accessing PD-Ballet (that they otherwise would not have been able to access through another mode), we believe will be extremely beneficial – because we will be reaching out to those that would other be disengaged. As part of this programme of research, one of the key aims is also to look at how we scale the arts interventions so that a larger number of individuals can benefit. Online delivery would be one way to do this, and while it was not our original intention to this (it was a result of COVID-19 that we had to adapt the interventions), this does now mean that we have designed the programme so it can be delivered in both ways (F-F and online) and so moving forward it may be the case that both approaches are offered and tailored so that individuals can pick the mode of delivery they prefer. We will examine this and the pros and cons of each approach in the papers focused on the write-ups of the trials and supporting research that will be undertaken alongside them.

11. Key omitted references

Barnish MS, Barran SM. A systematic review of active group-based dance, singing, music therapy and theatrical interventions for quality of life, functional communication, speech, motor function and cognitive status in people with Parkinson's disease. BMC Neurol. 2020 Oct 10;20(1):371. doi: 10.1186/s12883-020-01938-3.

McGill, A., Houston, S., & Lee, R.Y.W. (2019). Effects of a ballet-based dance intervention on gait variability and balance confidence of people with Parkinson's. Arts and Health, 11(2): 133-146

- The above references have been added

Reviewer: 2. Dr. Hilary Bungay

Thank you for submitting this protocol for review it is an interesting and well written protocol. I have a few minor points for consideration.

1. There are 25 authors listed as contributors – I do question if this is appropriate and whether the majority of these could be acknowledged for their contribution to the development of the study conception and design, rather than given authorship of this paper.

- Thank you for this comment, we understand why the reviewer has raised this because 25 authors would normally be a very high number to have on a paper. However, this paper focuses on two separate arts interventions for two different health conditions and is the world's largest study on scaling up arts interventions in healthcare. As such it involves a very large multidisciplinary team, all of whom play a key role in the success of this evaluation and have contributed to each step of the research process from co-designing the measures and methodology to being involved in data collection, write up and dissemination of findings. We therefore strongly feel to omit anyone from the author list would be doing them a disservice for all the hard work that they have contributed and will continue to contribute throughout this programme of research. We believe this research is an excellent example of a truly multidisciplinary endeavour where key stakeholders are involved in all elements of the research process.

2. The dates of the study are not included in the protocol.

- The dates have now been included

3. Limitations – the fact that this study appears to be London centric, and this is a potential limitation should be acknowledged, as implementation in other areas of the UK and internationally may face additional challenges.

- Thank you for this comment – this is a protocol paper and so we do not to include a discussion, but we will include a 'limitations' section in the discussion of the papers that focus on the write-up of each of trials' results. Within this section we will discuss the generalisability of the findings.

4. Page 4 line 17 – remove 'clinically' or give a specific example in relation to physical and psychological problems.

- This has been removed

5. P4 line 27 – the sentence starting 'While lacking…..' is unclear and depending on how it is read could seem contradictory.

- This sentence has been edited to make it clearer

6. P4 line 50 should be (for mums with PND), recommend removal of 'alternative (or conjunctive)'

from this sentence.

- These words have been removed

7. Page 5 line 5 would ‘purchasers’ rather than ‘payers’ be more appropriate?

- We have left the term ‘payers’ because we feel this is a better fit with the text, but thank you for this comment

8. Page 5 line 53 this is inconsistent with Table 2 ‘Comparator method; which states ‘standard treatment’. In addition, standard treatment would be a more appropriate comparator as recommending other similar activities could introduce confounding factors.

- The text in Table 2 has also been edited so this is clearer
- The rationale for recommending other similar activities was because, for M4Ms we wanted to dissect the effects of singing from social interaction for postnatal depression. By having mothers and babies go to other non-singing activities, we’re using a comparator that includes the social component but not the singing component that distinguishes M4M. In the same way, for PD-Ballet, we wanted to ensure that we are assessing the effects of the dance programme as opposed to the whole idea of the social interaction as we are really interested in the multimodality of dance and its effect on the non-motor symptoms of PD, which is something that has had very little coverage before.

9. Table 1: These are not strictly Art Therapies – recommend re-labelling. Also there are errors in referencing under Evaluation methods – should this be reference 59 – rather then 61? Similarly, in the Measures are the interview references correct. Recommend references are checked carefully in this table

- We have relabelled these as ‘arts interventions’
- The references have been checked

10. P11 – as this is a protocol paper it would be useful to include the power calculations and sample sizes. This could be as a supplementary file if the word count is a limiting factor.

- The power calculations are included in the parallel clinical effectiveness protocol papers for PD-Ballet and M4M and the references for these have been cited in the paper and listed in the reference list. A sentence is included in this paper on this too (‘A description of power and sample size calculations for M4M/PD-Ballet can be found in the corresponding clinical protocols31, 32’).

11. Page 12 – lines 3-6 developing a Theory of Change is usually undertaken prior to deciding on the measures/methods to use to determine whether the predicted outcomes have been achieved.

- When we first set out on developing this work, we used logic models to help us formulate appropriate methods and measures. However, we also wanted to use logic models and theory of change to help us understand what we need to do (e.g. in terms of implementation strategies) in order to be able to fully scale-up the arts interventions. In order to do this, we need to elicit feedback from those that have already been through the PD-Ballet and M4M programmes and those involved in delivering the programmes so that we can understand what some of the barriers are to access, continued involvement, implementation and longer-term sustainability. In this way our work on logic models and theory of change is more of continuous process throughout as our evidence and understanding of the programmes evolves

VERSION 2 – REVIEW

**REVIEWER**	Barnish, Max<br>University of Exeter, Exeter Medical School
**REVIEW RETURNED**	30-Sep-2021

**GENERAL COMMENTS**	I believe that the authors have taken into consideration all the comments offered, made improvements to the present manuscript, and noted suggestions that could inform future manuscripts.
